# Integrative -omics and HLA-ligandomics analysis to identify novel drug targets for ccRCC immunotherapy

**DOI:** 10.1186/s13073-020-00731-8

**Published:** 2020-03-30

**Authors:** Anna Reustle, Moreno Di Marco, Carolin Meyerhoff, Annika Nelde, Juliane S. Walz, Stefan Winter, Siahei Kandabarau, Florian Büttner, Mathias Haag, Linus Backert, Daniel J. Kowalewski, Steffen Rausch, Jörg Hennenlotter, Viktoria Stühler, Marcus Scharpf, Falko Fend, Arnulf Stenzl, Hans-Georg Rammensee, Jens Bedke, Stefan Stevanović, Matthias Schwab, Elke Schaeffeler

**Affiliations:** 1 Dr. Margarete Fischer-Bosch Institute of Clinical Pharmacology, Stuttgart, Germany; 2 University of Tuebingen, Tuebingen, Germany; 3 Department of Immunology, Institute for Cell Biology, University of Tuebingen, Tuebingen, Germany; 4 Clinical Collaboration Unit Translational Immunology, German Cancer Consortium (DKTK), University Hospital Tuebingen, Tuebingen, Germany; 5 German Cancer Consortium (DKTK), Partner Site Tuebingen, Tuebingen, Germany; 6 iFIT Cluster of Excellence (EXC 2180) “Image-Guided and Functionally Instructed Tumor Therapies”, University of Tuebingen, Tuebingen, Germany; 7 Department of Urology, University Hospital Tuebingen, Tuebingen, Germany; 8 Institute of Pathology and Neuropathology, University Hospital Tuebingen, Tuebingen, Germany; 9 Departments of Clinical Pharmacology, Pharmacy and Biochemistry, University of Tuebingen, Tuebingen, Germany

**Keywords:** ccRCC, Renal cell carcinoma, Ligandomics, HLA peptidome, Immunotherapy, Peptide vaccine, Cancer vaccine, Kidney cancer

## Abstract

**Background:**

Clear cell renal cell carcinoma (ccRCC) is the dominant subtype of renal cancer. With currently available therapies, cure of advanced and metastatic ccRCC is achieved only in rare cases. Here, we developed a workflow integrating different -*omics* technologies to identify ccRCC-specific HLA-presented peptides as potential drug targets for ccRCC immunotherapy.

**Methods:**

We analyzed HLA-presented peptides by MS-based ligandomics of 55 ccRCC tumors (cohort 1), paired non-tumor renal tissues, and 158 benign tissues from other organs. Pathways enriched in ccRCC compared to its cell type of origin were identified by transcriptome and gene set enrichment analyses in 51 tumor tissues of the same cohort. To retrieve a list of candidate targets with involvement in ccRCC pathogenesis, ccRCC-specific pathway genes were intersected with the source genes of tumor-exclusive peptides. The candidates were validated in an independent cohort from The Cancer Genome Atlas (TCGA KIRC, *n* = 452). DNA methylation (TCGA KIRC, *n* = 273), somatic mutations (TCGA KIRC, *n* = 392), and gene ontology (GO) and correlations with tumor metabolites (cohort 1, *n* = 30) and immune-oncological markers (cohort 1, *n* = 37) were analyzed to characterize regulatory and functional involvements. CD8^+^ T cell priming assays were used to identify immunogenic peptides. The candidate gene *EGLN3* was functionally investigated in cell culture.

**Results:**

A total of 34,226 HLA class I- and 19,325 class II-presented peptides were identified in ccRCC tissue, of which 443 class I and 203 class II peptides were ccRCC-specific and presented in ≥ 3 tumors. One hundred eighty-five of the 499 corresponding source genes were involved in pathways activated by ccRCC tumors. After validation in the independent cohort from TCGA, 113 final candidate genes remained. Candidates were involved in extracellular matrix organization, hypoxic signaling, immune processes, and others. Nine of the 12 peptides assessed by immunogenicity analysis were able to activate naïve CD8^+^ T cells, including peptides derived from *EGLN3*. Functional analysis of *EGLN3* revealed possible tumor-promoting functions.

**Conclusions:**

Integration of HLA ligandomics, transcriptomics, genetic, and epigenetic data leads to the identification of novel functionally relevant therapeutic targets for ccRCC immunotherapy. Validation of the identified targets is recommended to expand the treatment landscape of ccRCC.

## Background

Clear cell renal cell carcinoma (ccRCC) is the most prevalent subtype of kidney cancer, which affects over 400,000 individuals worldwide and causes around 175,000 deaths per year [1]. Especially for patients with advanced and metastatic disease, the prognosis is poor, with only 12% alive 5 years after diagnosis [2]. The reasons for the poor prognosis are the high intrinsic resistance of ccRCC tumors to conventional chemo- and radiotherapies, and the rapid development of resistance during currently applied targeted therapy. To combat therapy resistance, patients are commonly treated in several treatment lines with different targeted agents [3, 4]. Despite the availability of several targeted therapies, they mainly target two cellular pathways, that is the induction of angiogenesis through tyrosine kinases (TK) and the mTOR pathway. Initial response rates to anti-angiogenic TK inhibition (TKI) therapy vary between 30 and 40% [5], whereas around 10–30% of patients are sensitive to mTOR pathway inhibition [6, 7]. In most cases, the tumors recur under therapy or advance to metastatic disease [8]. The limited number of targeted pathways in ccRCC therapy leaves non-responding patients with almost no therapeutic options and therefore very poor prognosis. In 2015, the immune checkpoint inhibitor nivolumab was approved for ccRCC therapy and represented an important step forward in extending the therapeutic options in ccRCC therapy. The promising response rates of 25% [9], and the possibility of long-term effects, highlight the potential of immunotherapy in ccRCC management. Currently, combination regimens of immune checkpoint inhibitors and other agents are clinically investigated [4] and first studies support for instance the combination of atezolizumab and bevacizumab as a first-line treatment option for patients with advanced RCC [10]. Whether immune checkpoint inhibition, alone or in combination, is able to achieve long-term responses in advanced and metastatic ccRCC, similar to the responses observed in malignant melanoma, will emerge in the future.

First studies also indicated that a multi-peptide vaccine was beneficial in metastatic RCC patients when compared with a contemporary control cohort [11]. However, currently, no cancer peptide vaccine is approved for therapy due to missing clinical benefit [12], indicating the need for new approaches to select promising targets. One important requirement for the recognition and killing of cancer cells by specialized cells of the immune system, such as cytotoxic CD8^+^ T lymphocytes (CTL), is the presentation of tumor-specific peptides by major histocompatibility complex (MHC; also human leukocyte antigen (HLA)) molecules on the cell surfaces of the cancer cells. We have previously characterized the landscape of HLA-presented peptides, termed the HLA ligandome, of several cancer entities to identify HLA class I- and class II-restricted peptides [13–17]. In cancer therapy, such peptides are exploited in vaccines or adoptive T cell therapy (ACT) to activate CTLs and prime them for the killing of a patient’s cancer cells [18–20]. Naturally, the selection of appropriate peptides is critical for the induction of efficient CTL activation, recognition of cancer cells, and sustained anti-tumor responses. In contrast to melanoma or lung cancer, ccRCC is a cancer entity with a low mutational load [21], resulting in a low frequency of shared somatically mutated neo-epitopes between patients. Thus, tumor over-represented non-mutated self-peptides might represent an alternative to mutated neo-epitopes as targets in RCC immunotherapy.

**Fig. 1:**
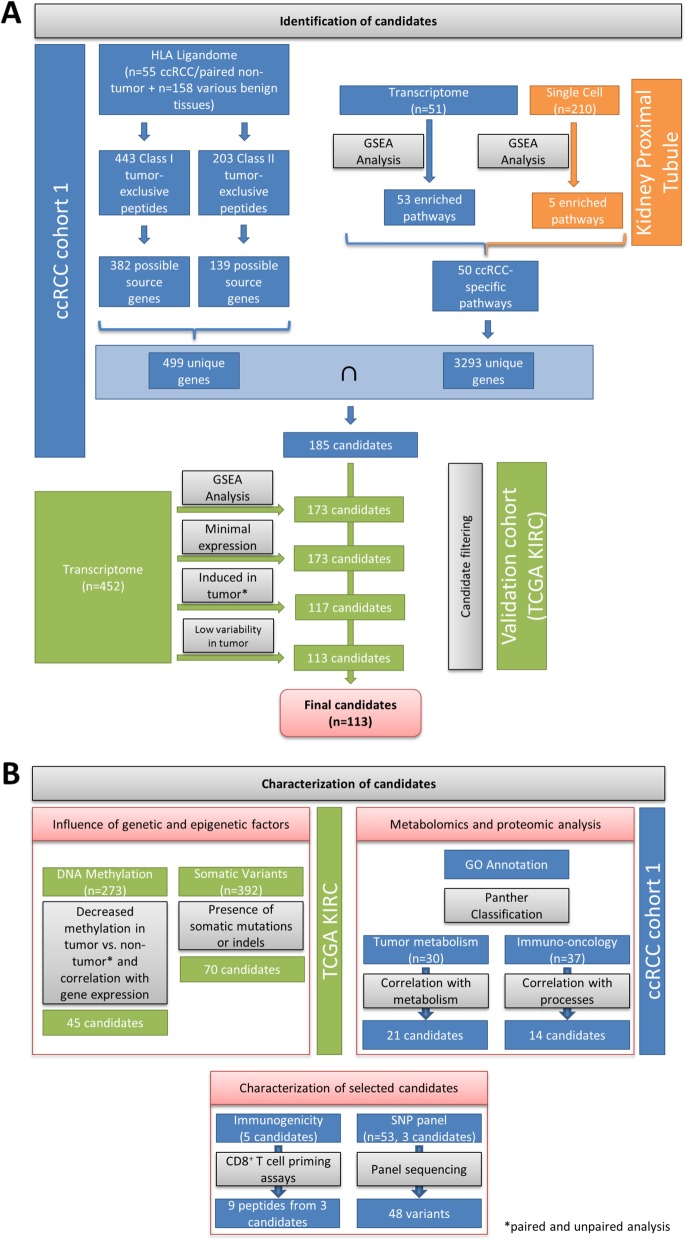
Workflow for candidate gene identification. **a** Genes were selected as candidate therapeutic targets if tumor-exclusive, frequent HLA-presented peptides were detected and if the source genes were involved in ccRCC-enriched pathways in ccRCC cohort 1. The candidate genes were validated and further filtered in a second ccRCC cohort (KIRC) from TCGA, yielding 113 candidate genes. **b** Comprehensive characterization of the 113 candidates by GO annotation, metabolomics, and proteomics analyses. Selected candidates were further tested for their immunogenicity and the presence of single nucleotide polymorphisms (SNPs) in patient cohort 1. The blue, orange, and green colors indicate whether data was generated from ccRCC patient cohort 1, single cell proximal tubule sequencing [22], or from TCGA KIRC cohort, respectively.

To aid the selection of appropriate peptides and gain further insight into ccRCC pathogenesis, we developed a workflow to identify HLA-presented ccRCC-specific peptides, with source genes involved in ccRCC-affected pathways (Fig. 1a). By selection of functionally relevant source genes, we expect to antagonize resistance development due to rapid peptide loss under therapeutic pressure. ccRCC-associated peptides were identified by MS-based HLA ligandome analysis of 55 ccRCC tissues and paired non-tumor kidney tissues, as well as 158 tissues of other healthy organs. In the same patient cohort, gene set enrichment analysis (GSEA) on whole transcriptome data was performed. ccRCC-specific peptide source genes were intersected with genes of functionally enriched pathways to obtain a list of candidate genes. The genes were validated in an independent ccRCC patient cohort from The Cancer Genome Atlas (TCGA) on the level of pathway enrichment, expression level, significant induction in tumors versus non-tumor tissues, and low variability of expression in tumor tissue, yielding 113 final candidate genes. All candidate genes were comprehensively characterized on different levels (Fig. 1b). Therefore, we investigated their potential regulation by DNA methylation and the presence of somatic mutations in tumors. Gene ontology (GO) analysis was performed for functional annotation, and the candidate genes were correlated with tumor metabolites and the expression of a set of onco-immune related proteins to refine their functions. Three of the candidate genes that are known to be frequently mutated in ccRCC were sequenced to identify potential neo-epitopes, which could represent targets for individualized treatment strategies. For selected candidate genes, non-mutated tumor-specific peptides were analyzed for their ability to induce naïve T cells, which is the basis for applicability in tumor peptide vaccines. Finally, the candidate target prolyl hydroxylase 3 (*PHD3*; *EGLN3*) was further investigated in different ccRCC cell culture models to evaluate its eligibility as drug target in ccRCC.

## Methods

### Patient cohorts

Primary ccRCC tumors and paired non-tumor renal tissues (*n* = 55) were collected at the Department of Urology, University Hospital Tuebingen, Tuebingen, Germany. Use of the tissue was approved by the ethics committee of the University of Tuebingen, and informed written consent was provided by each subject prior to surgical resection. Furthermore, data from patients with ccRCC (KIRC cohort, *n* = 452) of The Cancer Genome Atlas (TCGA) project were included [23]. Patient characteristics are summarized in Table 1.

**Table 1:** Patient cohorts and characteristics

	Cohort 1		TCGA	
	No. of patients	%	No. of patients	%
No. of patients	55		452	
Sex				
Male	37	67.3	290	64.2
Female	18	32.7	162	35.8
Age median (range)	70 (32–84)		61 (29–90)	
Stage				
1	24	43.6	221	48.9
2	3	5.5	44	9.7
3	14	25.5	116	25.7
4	14	25.5	69	15.3
NA	–	–	2	0.4
Primary tumor				
1	26	47.3	227	50.2
2	4	7.3	56	12.4
3	24	43.6	164	36.3
4	1	1.8	5	1.1
N				
0	46	83.6	203	44.9
1/2	7	12.7	11	2.4
X	2	3.6	238	52.7
M				
0	42	76.4	377	83.4
1	13	23.6	68	15.0
X	–	–	7	1.5
G				
1	8	14.5	10	2.2
2	38	69.1	188	41.6
3/4	9	16.4	251	55.5
X	–	–	1	0.2
NA	–	–	2	0.4
Median follow-up time [years] (range)	2.9 (0–10.1)		3.5 (0–12.4)	
Overall survival^a^ [years]				
Deceased	28	50.9	145	32.1
Alive	26	47.3	307	67.9

*Abbreviations*: *N* regional lymph nodes, *M* distant metastasis, *G* grading, *NA* not available

^a^Information on overall survival was not available for all patients

### Analysis of HLA ligands by LC-MS/MS and identification of ccRCC-presented peptides

HLA ligandomics was performed by reversed phase liquid chromatography coupled mass spectrometry as previously described [15, 24, 25]. The monoclonal antibodies W6/32, Tü39, and L243 (in-house production at the Department of Immunology, University of Tuebingen, Tuebingen, Germany) were used for immunoaffinity purification of HLA class I and II peptide complexes. Five technical replicates were measured per sample. For annotation, data was processed against the human proteome as available from the Swiss-Prot database (release: September 27, 2013; 20,279 reviewed protein sequences contained) [26] within the Proteome Discoverer (v1.3, Thermo Fisher Scientific) software. The search was not restricted to enzymatic specificity, and oxidized methionine was enabled as dynamic modification. Percolator [27] assisted false discovery rate (FDR) was set at 5%, and results restricted to rank 1 (best match for each spectra) and length of 8–12 amino acids for HLA class I and 9–25 amino acids for class II peptides. NetMHCpan-3.4 [28] (rank < 2 or 500 nM) and SYFPEITHI [29] (***≥*** 60% of maximal score) were used to define the HLA ligands. The average purity, which determines the amount of peptides with HLA binding motifs for the respective HLA allotypes of the patients in comparison to all identified peptides, is 90% (range 78–97%). The uniprot mapping tool [30] was used to assign Entrez Gene IDs to the uniprot IDs. Non-mapped uniprot IDs were manually assigned to their respective Entrez Gene IDs. A list of peptides from the 113 candidate genes identified in this study is provided in Additional file 1: Tab. S1.

### HLA typing

HLA typing of patients from cohort 1 and healthy donors for PBMC isolation was carried out by the Department of Hematology and Oncology, University of Tuebingen, Tuebingen, Germany. An overview of the HLA typing is shown in Additional file 2: Tab. S2.

### Transcriptome analyses

Genome-wide mRNA expression analysis of tumor samples was performed using the Human Transcriptome Array 2.0 (Thermo Fisher Scientific) as previously described [31, 32]. In brief, RNA was purified from fresh-frozen ccRCC tissue using the mirVana™ miRNA Isolation Kit (Life Technologies) and microarrays were processed according to the manufacturer’s procedure (Thermo Fisher Scientific). Quality control and preprocessing using RMA normalization were carried out as previously described [31, 32]. HTA 2.0 transcript cluster IDs were assigned to corresponding Entrez Gene IDs with the Affymetrix annotation file downloaded from NetAffx™ Analysis Center [33]. Entrez Gene IDs with multiple probes were summarized by calculation of the mean expression per sample.

### Preparation of expression data from single cell kidney proximal tubules

Proximal tubule single cell RNA sequencing data was taken from Young et al. [22] and prepared as described with the R packages Seurat (v. 3.0.0) [34, 35], scran (v. 1.8.4) [36], and sva (v. 3.28.0) [37]. The ENSEMBL gene identifiers were matched to their respective Entrez Gene IDs with Genome wide annotation for Human package (org. Hs.eg.db; v. 3.6.0) [38] in R [39]. Entrez Gene IDs with multiple probes were summarized by calculation of the mean expression per sample.

### Processing of RNA-seq KIRC data from TCGA for GSEA

Transcriptome profiling data (“HTSeq – FPKM-UQ”) were downloaded from the Genomic Data Commons Portal [40] on December 9, 2016. For analysis in this study, the data was transformed to log_2_(FPKM) values. The Ensemble Gene IDs were mapped to their respective Entrez Gene IDs using the NCBI Gene record database [41]. Entrez Gene IDs with multiple probes were summarized by calculation of the mean expression per sample.

### GSEA

Gene signatures for gene set enrichment analysis (GSEA) were taken from the Molecular Signatures Database version 6.1 [42]. We included the hallmark gene sets, the KEGG, Biocarta, and Reactome gene sets from the C2 curated gene set collection, the C4 computational gene sets, and the C6 oncogenic gene sets in the analysis (Additional file 2: Tab. S3). In addition, immunological modules were included from a publication of Nath et al. [43]. The Illumina probe identifiers were mapped to the respective Entrez Gene IDs with the Illumina HumanHT-12 v4 annotation file (2017/11/30). We used the Entrez Gene IDs as identifiers in all analyses. The GSEA was computed with the GSVA package [44] using the method of single-sample GSEA (ssgsea) with normalization (v. 1.32.0). Gene sets with an enrichment score ≥ 0.5 in 80% of cohort samples were considered to be enriched. In case of the single cell proximal tubule region dataset, only samples with at least 3000 measured genes were included in the GSEA. Of note, the coverage of gene signatures was lower in the single cell data than in bulk gene expression data. The GSEA was run individually for every sample to include only genes with expression values in the respective sample.

### TCGA DNA methylation data preparation and analysis

Whole genome DNA methylation data and the corresponding probe map from TCGA KIRC cohort were downloaded from UCSC Xena browser [45]. For the investigated genes, associated CpG sites were retrieved from the probe map.

### TCGA somatic mutation data preparation and analysis

Somatic mutations were obtained from Kandoth et al. [46]. The authors prepared a set of strictly filtered somatic variants for selected TCGA cancer entities. For 392 of 452 TCGA ccRCC tumors used in this work, mutation data were available.

### NGS panel sequencing

Sequencing of the candidate genes *MET*, *TSC2*, and *RB1* was performed using a TruSeq Custom Amplicon gene panel. The panel was designed using Design Studio (Illumina) and includes probes to sequence regions of interest in 32 genes which were known to be frequently mutated in RCC samples from TCGA or identified in other RCC studies. High-quality DNA was isolated from fresh-frozen tissue of cohort 1 using the QIAamp DNA kit (Qiagen). Library preparation was performed according to the TruSeq Custom Amplicon Low Input protocol. The final libraries were sequenced on the MiniSeq platform (Illumina) with a median coverage of 1600. Further processing was performed on the MiniSeq using the Base Space Tru Seq Amplicon App for alignment and variant calling. The data analysis software Illumina Variant Studio 3.0 was used for variant annotation, filtering, and classification. Single nucleotide variants (SNVs) and small insertions and deletions (indels) were analyzed for the target genes *MET*, *TSC2*, and *RB1*.

### DNA methylation analyses through MALDI-TOF MS

Bisulfite conversion and subsequent MALDI-TOF MS was used to measure the DNA methylation levels at selected CpG sites in the *EGLN3* gene region of samples from the ICEPHA patient cohort, as previously described [47]. Primer sequences are provided upon request.

### CD8^+^ T cell in vitro priming assays and tetramer staining

To investigate the immunogenicity of tumor-associated peptides, peripheral blood mononuclear cells (PBMCs) were isolated from whole blood of 6 healthy donors using a Ficoll (Merck Millipore) density gradient. CD8^+^ T cells were isolated from HLA-matched PBMC cultures by magnetic cell separation using α-CD8 beads (Miltenyi Biotech) according to the manufacturer’s instructions. For priming, 1 × 10^6^ T cells were incubated with 2 × 10^5^ artificial antigen-presenting cells (aAPCs) coated with HLA-bound peptides (donor-matched HLA:peptide monomers, in-house production) and α-CD28 in the presence of 5 ng/ml IL-12 (PromoCell) as previously described [48]. Priming was repeated weekly for 4 weeks. Two days after each stimulation, 40 U/ml IL-2 and 5 ng/ml IL-7 were added. Monomers with HLA ligands of interest were produced by UV-mediated exchange as previously described [49]. CD8^+^ T cell priming was evaluated by tetramer staining using PE-labeled tetramers, PerCP-labeled α-CD8 monoclonal antibody, and the aqua live/dead stain solution (Thermo Fisher Scientific). Data was acquired on a FACS Canto II analyzer and evaluated using the software FlowJo 10.0.7. A 3-fold larger and distinct tetramer-positive population compared to the negative control was considered positive priming.

### Targeted metabolomics of ccRCC tissues

Targeted metabolomics/lipidomics analysis of tissue samples was performed using liquid chromatography and flow injection analysis mass spectrometry by Biocrates Life Sciences (Innsbruck, Austria) as previously described [50]. In total, 204 metabolites could be quantified (Additional file 2: Tab. S4). For analysis, the absolute metabolite quantifications were glog_2_-transformed [51]. The metabolites were assigned to molecule classes or cellular pathways as indicated in Additional file 2: Tab. S4.

### Olink Proteomics

Targeted proteomics analysis of tumor tissues was performed using the proximity extension assay (PEA) with the immuno-oncology panel by Olink Proteomics (Uppsala, Sweden). The method allowed relative quantification of 92 proteins (Additional file 2: Tab. S5), reported as normalized protein expression (NPX). Proteins were assigned to functional groups according to the company’s specifications. Group assignments are given in Additional file 2: Tab. S5.

### Statistical analyses

All statistical analyses were performed in R Studio (version 1.0.153) [52] with R [39], with additional packages Hmisc (v. 4.2–0) [53] and gplots (v. 3.0.1.1) [54].

### Cell culture methods

#### Cell lines

A498, 786-O, and Caki1 were purchased from CLS Cell Lines Service (Eppelheim, Germany). A498 cells were cultivated in EMEM (Lonza) supplemented with 10% FBS (Merck) and 2 mM l-glutamine (Lonza). 786-O cells were cultivated in RPMI 1640 medium (Lonza) supplemented with 10% FBS and 2 mM l-glutamine. Cell lines were routinely tested for mycoplasma infection using a PCR-based test (Venor® GeM Classic, Minerva Biolabs GmbH), and authentication of cell lines was performed using the PowerPlex® 21 System (Promega) according to the manufacturer’s protocol.

#### siRNA-mediated knockdown of *EGLN3* gene expression

*EGLN3* knockdown was performed using the siGENOME siRNA pool 112399 (GE Healthcare) together with the DharmaFECT 1 transfection reagent (GE Healthcare). The siRNA pools siGENOME Non-targeting siRNA Pool #1 (Ctr. 1) and ON-TARGETplus Non-targeting Pool (Ctr. 2) were used as negative control siRNA pools. Transfections were carried out as specified in the manufacturer’s protocol. In brief, cells were seeded in culture dishes and transfected the following day at a confluency of ~ 60% with a final concentration of 25 nM siRNA. Assays were performed 48 h after transfection.

#### Untargeted metabolomics analysis

For metabolomics analysis, the 786-O kidney carcinoma cell line was used. Cells were seeded and transfected in T25 cell culture flasks and harvested 72 h after transfection. Two hours before harvest, the cell culture medium was replaced by Opti-MEM serum-free medium (Thermo Fisher Scientific). Cells were washed with PBS, detached with StemPro Accutase (Thermo Fisher Scientific), taken up in ice-cold PBS, and collected by centrifugation for 10 min at 500×*g* and 4 °C. The supernatant was discarded, and the pellets transferred to cryogenic tubes. After another centrifugation step, the cell pellets were flash-frozen in liquid nitrogen. The following steps were performed on ice or at 4 °C, if not stated otherwise. For metabolite extraction, pellets were resuspended in 160 μl methanol to water (1:1). To disrupt cells, cells were sonicated 4 × 30 s and vortexed for 10 min. Subsequently, cells were centrifuged for 10 min at 15000 rpm. The supernatant (aqueous phase) was transferred to new tubes and evaporated with N_2_. The metabolites were reconstituted with acetonitrile to water (95:5) at the day of the measurement. A quality control sample (pool of all samples) was included in the analysis, and samples were randomized prior to the analysis. For a detailed description of the analysis method by LC-QTOF-MS, please refer to Leuthold et al. [55]. MassHunter Profinder software (Agilent Technologies) was used for data analysis and feature extraction. A total of 1522 features were identified in positive ionization mode. An internal library [55] and the METLIN Metabolomics online database [56] were used to assign metabolites to measured features. For analysis of differently abundant metabolites, data was exported and analyzed with R and R studio, with the base package stats (v. 3.4.0) and the additional package beeswarm (v. 0.2.3) [57]. Data was log_2_-transformed. To calculate the significance of differences between test and control groups, Student’s *t* test was used and *p* values were corrected for multiple testing with the Benjamini-Hochberg method.

#### Cell replication

The incorporation of bromdesoxyuridine (BrdU) into DNA was detected to measure cell replication via FACS analysis. Therefore, siRNA transfected cells were incubated with 10 μM BrdU for 1 h and subsequently fixed and stained with the anti-BrdU primary antibody B44 (BD Biosciences) and an Alexa Flour 488 goat anti-mouse secondary antibody (Jackson ImmunoResearch). DNA content was stained with propidium iodide. Analysis was performed with the FACS Calibur and the CellQuest Pro software (BD Biosciences).

#### Apoptosis

FITC-coupled Annexin V (BD Biosciences) was used to detect apoptotic cells by FACS analysis. Propidium iodide was used for DNA staining. FACS analysis was performed with the FACS Calibur and the CellQuest Pro software (BD Biosciences).

#### Seahorse glycolysis

To measure cellular glycolysis, extracellular flux analysis was performed with the Seahorse XF96 Extracellular Flux Analyzer (Agilent Technologies) and the Glycolysis Stress Test kit (Agilent Technologies), according to the manufacturer’s instructions. Cells were seeded and transfected in Seahorse XF96 microplates, to be compatible with the XF instrument. At the day of the assay, the cell culture medium was exchanged to buffer-free Base medium (Agilent Technologies) supplemented with 1 mM l-glutamine and the cell plate equilibrated for 1 h in a non-CO_2_ incubator. During the assay, 10 mM glucose, 1 μM oligomycin, and 50 mM 2-DG were serially added to the cell medium and the response in extracellular acidification rate (ECAR) was measured. After the experiment, the growth area of the cell layer in each well was determined and used for normalization of the ECAR measurements. Analysis was carried out with Wave 2.4 software (Agilent Technologies).

#### Seahorse mitochondrial function

The oxygen consumption rate (OCR) of cells was measured to assess mitochondrial respiration. Therefore, the Seahorse XF96 Extracellular Flux Analyzer and the Mito Stress Test kit were used (Agilent Technologies), following the manufacturer’s instructions. The assay workflow is similar to the Glycolysis Stress Test described above; only the Base medium is supplemented with 2 mM l-glutamine, 10 mM glucose, and 1 mM Na-pyruvate. The compounds that are serially added during the assay are 1 μM oligomcyin, 0.5 μM FCCP, and 0.45 μM rotenone/antimycin A. OCR measurements were again normalized to the cell growth area per well.

#### Spheroid formation assay

To measure the ability of cells to form stable spheroids, cells were seeded in Poly (2-hydroxyethyl methacrylate) coated round-bottom 96-well plates at 500–10,000 cells per well. Images were taken at days 1 and 4 after seeding.

#### Cell viability assay

For assessment of cell viability, 3000–4000 cells were seeded in a 96-well plate in 150 μl standard growth medium. siRNA transfection was carried out the following day. Forty-eight hours after transfection, 15 μl WST-1 reagent (Sigma-Aldrich) was added per well and absorbance at 440 and 620 nm measured 30 min (A498/786-O) or 2 h (Caki1) later. For analysis, the 620-nm absorbance was subtracted from the 440 absorbance.

#### Statistical analysis of data from cell culture experiments

Replicate FACS experiments to measure cell replication and apoptosis were summarized by calculation of the mean and standard deviation of individual experiments. Two-tailed Student’s *t* test was used to assess statistical significance, with a *p* value threshold of 0.05. In the case of the extracellular flux analyses, the means and standard deviations were calculated per experiment which included 2–3 replicate wells per condition. To summarize replicate experiments, mean values of the individual experiments were calculated and the standard deviation was computed using the following formula: $\Delta  =\sqrt{\frac{{\mathrm{sd}}_1^2+{\mathrm{sd}}_2^2+\dots +{\mathrm{sd}}_n^2}{n}}$, where sd is the standard deviation and *n* the number of replicate experiments. Two-tailed Student’s *t* test was used to assess statistical significance, with a threshold of 0.05.

## Results

### Over-represented self-peptides in ccRCC determined by HLA ligandomics

**Fig. 2:**
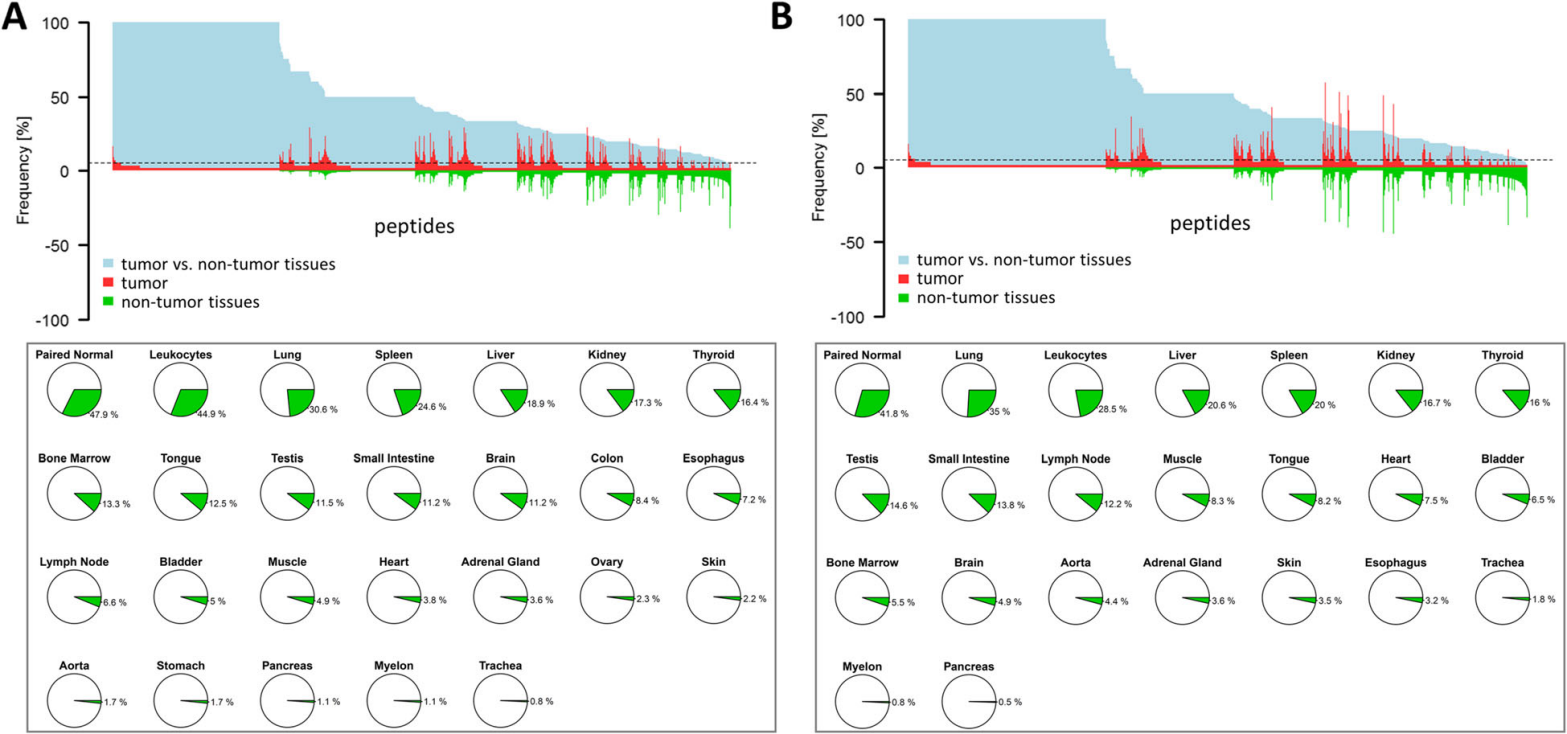
Over-represented HLA class I and II peptides in ccRCC. **a** Upper plot: the *x*-axis shows all HLA class I-presented peptides identified by HLA ligandomics in ccRCC tissues of patient cohort I. The *y*-axis shows the percentage of samples with the respective peptides. Peptides detected in tumor tissue are plotted as positive percentages in red, and peptides detected on non-tumor tissues are plotted in negative percentages in green. The light blue shaded area represents the tumor exclusivity, where 100% indicates ccRCC-specific presentation. Lower plot: percentages of peptides detected in non-tumor tissues, comprising tumor-paired non-tumor kidney tissue (patient cohort I, *n* = 55) and healthy tissue samples from various organs (*n* = 158), related to all peptides detected in proliferation reagentccRCC tissue of cohort I. **b** The same presentation for HLA class II-presented peptides

Since ccRCC is known as a cancer entity with low mutational load [21], we aimed to identify tumor over-represented non-mutated peptides. HLA class I- and II-bound peptides were extracted from 55 and 49 ccRCC tissues, respectively, as well as paired adjacent non-tumor tissues of patient cohort 1 (Table 1), and analyzed by mass spectrometry. Analysis of ccRCC tissue yielded a total of 34,226 unique class I and 19,325 unique class II peptides from 10,524 and 4053 possible source proteins, respectively. The average purity of HLA class I-restricted peptides was 90% (range 78–97%), demonstrating good quality of sample preparations. To identify ccRCC-specific peptides, a library of HLA class I and II ligands from 158 tissues of healthy organs and leukocyte preparations served as a control pool to exclude globally presented peptides (Fig. 2) [58]. The highest overlap of ccRCC HLA class I-presented peptides was observed with tumor adjacent benign kidney tissue from the same patients (47.9%), followed by leukocytes (44.9%) and lung tissue (30.6%) (Fig. 2a, lower panel). ccRCC HLA class II-presented peptides also showed the strongest overlap with tumor-paired adjacent benign kidney tissue (41.8%), lung tissue (35.0%), and leukocytes (28.5%) (Fig. 2b, lower panel). Peptides that were detected in at least three ccRCC tumor tissues and in none of the non-tumor tissues or leukocyte preparations were considered ccRCC-specific. The analysis revealed 443 ccRCC-specific class I and 203 class II peptides, which were mapped to 382 and 139 possible source genes, respectively. The median number of ccRCC-associated HLA class I- and II-presented peptides was 25 (range 1–113) and 9 (range 0–45) peptides, respectively. Of note, the presence of tumor-associated class I and class II peptides did not significantly correlate with patient survival (Additional file 2: Fig. S1), which might be due to the generally tolerogenic and immune suppressive microenvironment in ccRCC [59]. Finally, to receive a list of candidate targets for immunotherapeutic approaches, the source proteins of HLA class I and II peptide targets were combined, yielding 499 proteins, which are encoded by 499 unique genes (Fig. 1a).

### Source genes of ccRCC-presented peptides are involved in pathways that are activated by ccRCC tumors

**Fig. 3:**
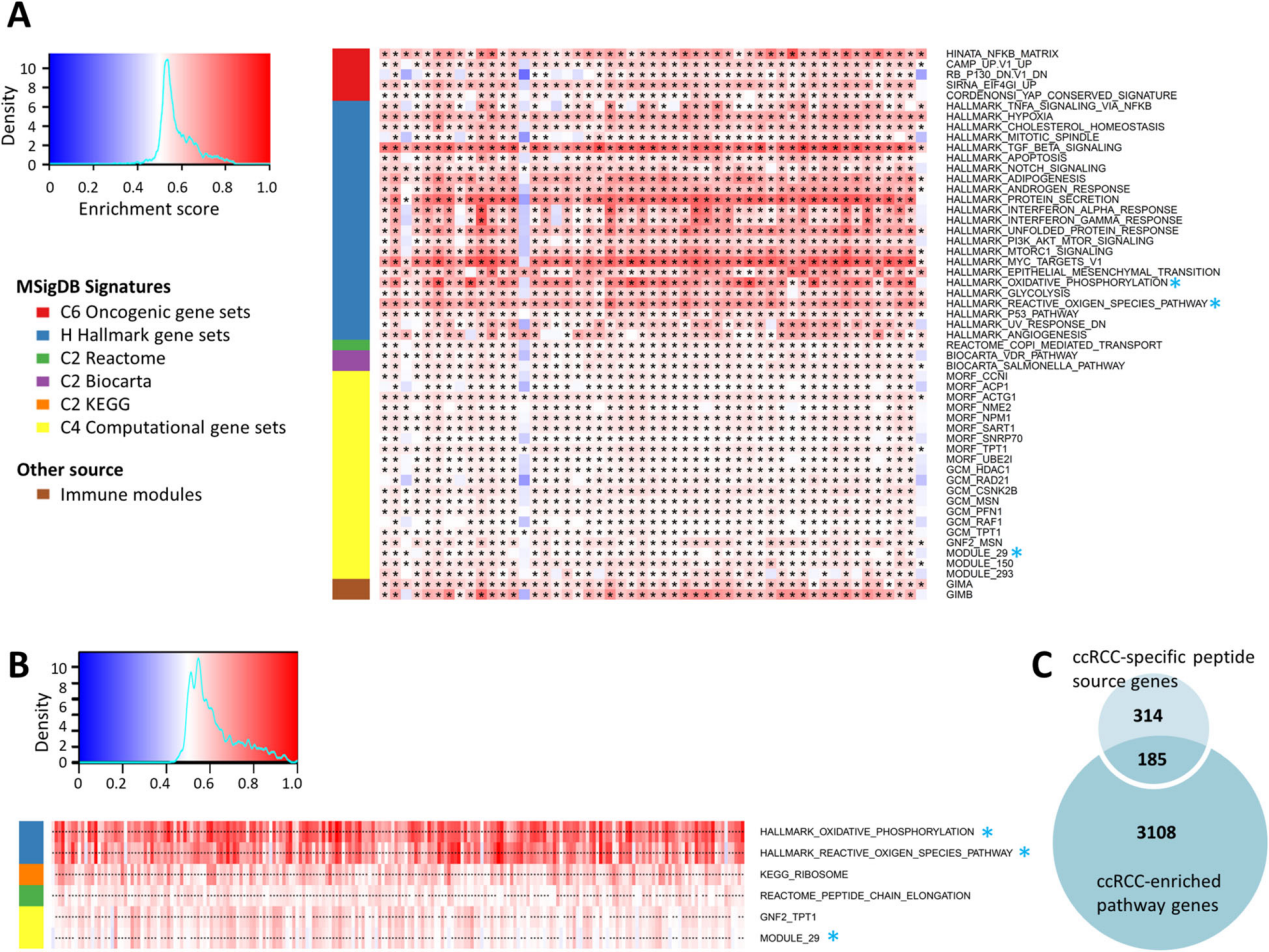
Gene set enrichment analysis (GSEA) reveals ccRCC-enriched pathways. **a** Heatmap of pathway enrichment scores in ccRCC patient cohort 1. Shown are only signatures that were enriched in the cohort 1, defined by an enrichment score of ≥ 0.5 (marked by asterisks) in at least 80% of cohort patients. The signatures that were removed after comparison with the enrichment analyses in the proximal tubule cells are marked by blue asterisks. **b** Heatmap of enrichment in the proximal tubule cells with data taken from Young et al. [22]. Shown are only those signatures that were enriched in the cohort with a score of ≥ 0.5 (marked by asterisks) in at least 80% of cohort samples. Sources of gene signatures can be derived from the color bar to the left of the heatmap with the legend printed in **a**. Signatures that overlapped with enriched signatures of tumor tissues from cohort 1 are marked by blue asterisks. **c** Overlap of source genes of ccRCC-specific peptides and genes from ccRCC-enriched signatures

In order to identify ccRCC-associated pathways and processes, we selected 2182 individual gene signatures from Hallmark, C4 Cancer Module, and C6 Oncogenic Signature collections, and the Biocarta, Kegg, and Reactome pathways from the C2 Curated Gene Set collection of the Molecular Signatures Database (MSigDB) [42], as well as eight immune module signatures published in a study by Nath et al. [43]. The signatures were applied in single-sample gene set enrichment analyses (ssGSEA) of whole transcriptome data from 51 tumor tissues of patient cohort 1 for which HLA-ligandomics data and high-quality RNA was available (Fig. 1). The ssGSEA method allows the calculation of pathway activity scores in individual samples on the basis of gene expression levels [60]. For our purpose, a certain pathway or process was considered to be activated in an individual sample if the ssGSEA enrichment score exceeded 0.5 in that sample. In addition, as we were interested in targets broadly applicable in the population of ccRCC patients, at least 80% of the cohort patients needed to show pathway activation. Applying these criteria, 53 of the 2182 individual gene signatures were considered activated in ccRCC cohort 1 (Fig. 3a). The activated signatures were part of the Hallmark, the C4 Cancer Module, and the C6 Oncogenic Signature collections, as well as the Reactome and Biocarta pathways and the immune modules.

To ensure that the observed activated pathways are a consequence of the malignant transformation of the tumor tissue and not a characteristic of the cell type of tumor origin, the same enrichment analysis was applied to a set of single cell sequencing data from the kidney proximal tubule region of 5 patients (data taken from Young et al. [22]), representing the cell type of origin for ccRCC [23, 61]. We chose single cell proximal tubule gene expression data over bulk normal kidney sequencing as normal kidney tissue is composed of functionally distinct cell types, with widely differing gene expression profiles [62]. Five signatures were enriched in the collection of 210 proximal tubule cells, three of which overlapped with the enriched signatures in cohort 1 (Fig. 3b). Therefore, these three overlapping signatures (blue asterisks in Fig. 3a, b) were excluded, leaving 50 ccRCC-enriched signatures. Of the 3293 genes associated with these signatures, 185 overlapped with the 499 genes from the HLA ligandome analysis (Fig. 3c) and were considered in further analysis steps as potential immunotherapeutic targets associated with ccRCC pathogenesis.

### Validation of targets in an independent ccRCC patient cohort from The Cancer Genome Atlas

**Fig. 4:**
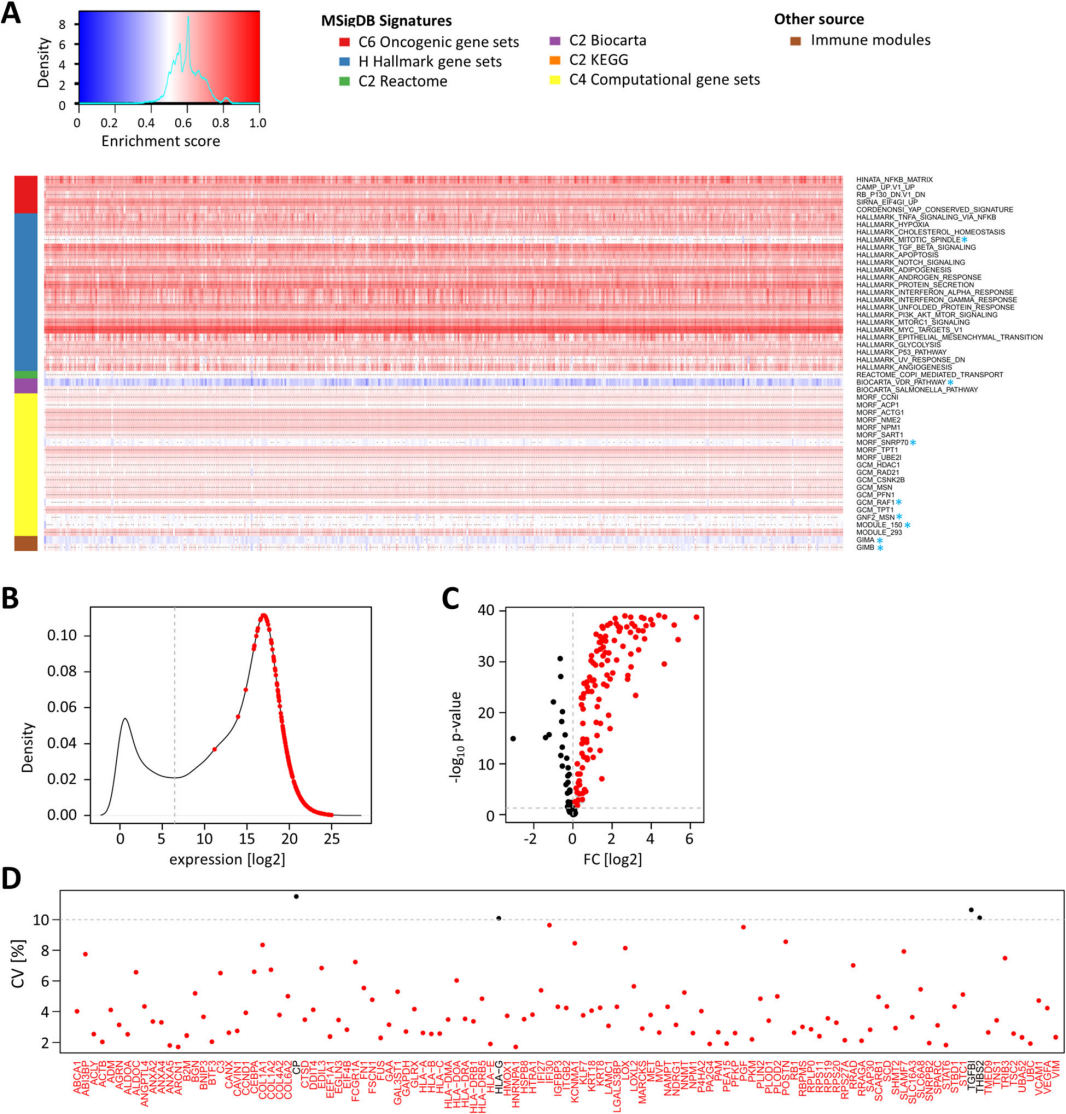
Validation of targets in an independent ccRCC patient cohort from The Cancer Genome Atlas (TCGA). **a** Heatmap of enrichment scores in TCGA patient cohort (KIRC). Shown are 50 signatures that were identified as ccRCC-enriched in analysis of cohort 1 and proximal tubule cells. Signatures that were not enriched by an enrichment score of ≥ 0.5 in at least 80% of TCGA cohort samples are marked by blue asterisks. Genes exclusively included in those signatures were removed in further analyses. **b** Kernel density estimate of mean log_2_ gene expression levels in TCGA ccRCC tumor samples. Mean log_2_ expression levels of the 173 candidate genes are marked in red. The minimal expression threshold was set at the local minimum of the estimated frequency distribution at a log_2_ expression of 6.5 (gray vertical intersected line). All candidates passed the threshold. **c** Volcano plot of gene expression fold changes in tumors compared to non-tumor tissues. Shown are the values of the unpaired analysis. Expression values that passed the set thresholds at FC > 0 and *p* < 0.05 in both unpaired and paired analyses are marked in red. **d** Plotted are the coefficients of variation (CV) in percent. The intersected line marks the set threshold of CV ≤ 10% for candidate selection. Candidates that did not pass the threshold are printed in black

To validate the 185 candidate genes, we used publicly available data from a ccRCC cohort of The Cancer Genome Atlas (TCGA) project, comprising tumor tissues of 452 patients and additional paired non-tumor tissues of 67 patients (Table 1). Analogous to cohort 1, ssGSEA was performed, supporting the enrichment of 42 of the 50 pathways from the analysis of cohort 1 (Fig. 4a), thereby confirming 173 of the candidate genes. All of the 173 genes were expressed above a minimal expression threshold in TCGA patient cohort, defined by the local minimum of the log_2_ FPKM-UQ frequency distribution (Fig. 4b). To select for genes that are induced in ccRCC tumors compared to adjacent non-tumor tissue, the expression differences between tumor and non-tumor tissue were calculated. One hundred seventeen candidates were significantly induced (*p* < 0.05) in tumor tissues in paired and unpaired analyses (Fig. 4c). Because we aimed to identify potential targets that are present in the majority of patients with ccRCC, we finally filtered the candidate gene list for low expression variability in tumors of TCGA patient cohort. Only four of the117 genes showed a coefficient of variation (CV) ≥ 10%. The remaining 113 genes comprised the final candidate gene list (Fig. 4d).

### Characterization of candidate targets: regulation of gene expression and gene function

Tumor cells frequently display an altered epigenetic landscape, with a global impact on gene expression. In ccRCC, DNA hypermethylation is frequently observed in promoter regions of tumor suppressor genes, with impact on tumor stage and grade [63, 64]. We investigated whether the candidates of our analysis workflow are affected by altered DNA methylation in ccRCC tumors, which could explain their upregulation in tumor tissues. Altered methylation at specific sites could offer the opportunity of epigenetic modulation, in addition to immunologic targeting as therapeutic strategy. Therefore, we took again advantage of the publicly available ccRCC dataset from TCGA. For 273 of the cohort tumor samples and 143 of the non-tumor samples, DNA methylation *β*-values were available at the time of data analysis. We calculated the difference of DNA methylation in tumor and non-tumor tissues and determined the correlation with gene expression in tumor tissues. Forty-five genes with significant negative (*p* < 0.05) correlation of DNA methylation and gene expression (Spearman’s correlation coefficient < − 0.3) were considered to potentially be regulated by DNA methylation in tumor tissue (Additional file 2: Tab. S6 and Fig. S2). Since the effect of clinically approved DNA demethylating agents like decitabine on the processing and presentation of antigens has recently been proposed [65, 66], epigenetic therapy might induce the expression of the corresponding immunogenic antigens in ccRCC.

In addition to DNA methylation, somatic mutations in genes might affect their expression, stability, and function, as well as the design of target-specific therapeutics. Therefore, we investigated the frequency of somatic variants including point mutations and small indels in the candidate target genes. From TCGA project, data was available for 392 of the ccRCC samples. Seventy-seven of the candidate genes showed somatic variants in at least one patient (Additional file 2: Tab. S7 and Fig. S3). None of the candidate genes were mutated in more than 8 (2.0%) patients of the cohort, which is in line with the reported low mutational rate in ccRCC tumors [21]. Additionally, only 5 mutations were shared between two or more patients (Additional file 2: Tab. S8). As shown in Additional file 2: Fig. S3, somatic point mutations or indels resulting in protein alterations only occur in single cases. Three of the candidates, namely *MET*, *TSC2*, and *RB1*, that were affected by somatic mutations in TCGA patient cohort are genes found to be frequently mutated in ccRCC, either in literature [67–69] or in own data analyses (unpublished data), and were therefore selected for deep sequencing in patient cohort 1. Taken together, 13, 25, and 1 patients of the 53 sequenced patients displayed non-synonymous coding variants in exonic regions of *MET*, *TSC2*, and *RB1*, respectively (Additional file 2: Tab. S9). Forty-five of the detected variants result in amino acid substitutions, potentially leading to patient and tumor-specific mutated peptides, the so-called neo-epitopes or neo-antigens. Neo-epitopes are potent inducers of T lymphocytes and mediators of anti-tumor immunity [70–73]. Their utilization in cancer therapy is, however, complicated by the fact that most neo-epitopes are patient-specific, requiring a personalized timely discovery and manufacturing pipeline for each individual patient [74]. Shared neo-epitopes have been studied for the therapy of glioblastoma [75], acute myeloid leukemia [76], and chronic myelogenous leukemia [77], representing cancer entities with common somatic mutations. Most of the mutations of *MET*, *TSC2*, and *RB1* that were detected in cohort 1 of this study were private and would therefore not qualify for a broadly applicable “off-the-shelf” cancer vaccine or ACT approach.

### Characterization of candidate targets: functional consequences in ccRCC

**Fig. 5:**
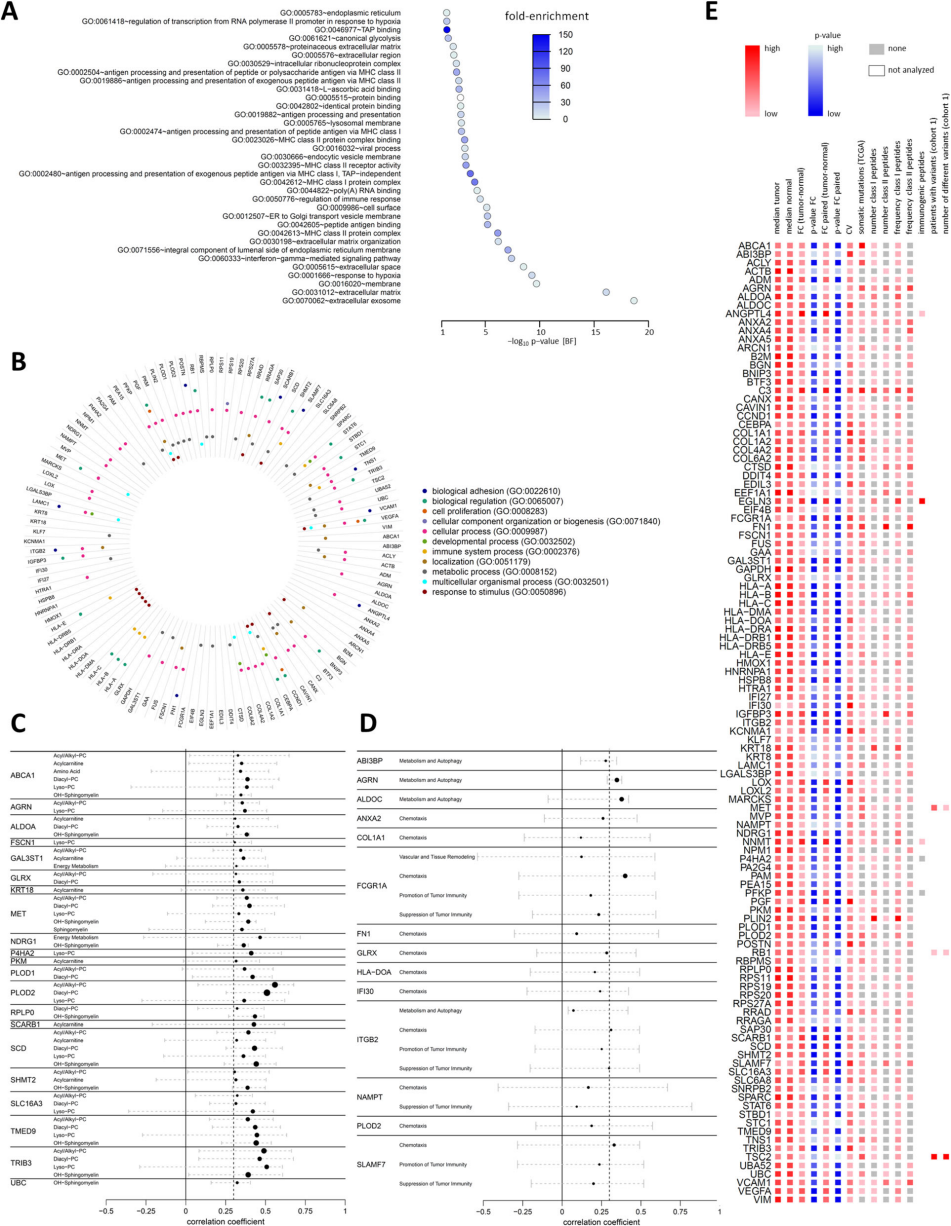
Functional associations of candidate target genes. **a** DAVID GO analysis of the candidate genes. Plotted are the Bonferroni (BF) corrected *p* values of the enrichment. The colors of the dots represent the enrichment fold changes. **b** Circular bar graph of biological functions assigned to the candidate genes by PANTHER GO analysis. **c** Correlations of candidate genes with tumor metabolites. Plotted are the medians and ranges of Pearson’s correlation coefficients of the candidates with the metabolites of the indicated metabolite classes (Additional file 2: Tab. S4). Only significant (*p* < 0.05) correlations with median correlation coefficients of ≥ 0.3 are plotted. **d** Correlation of candidate genes with immuno-oncological processes. An overview of markers descriptive of the processes is given in Additional file 2: Tab. S5. Plotted are the medians and ranges of Pearson’s correlation coefficients if the candidate genes correlated with a coefficient of ≥ 0.3 in at least 25% of samples. **e** Overview of the final 113 candidates. The color range indicates for each of the plotted parameters the respective values. Exact values can be retrieved from Additional file 2: Tab. S10

The selection of candidate genes in this study was based on the presence of gene-derived HLA-presented peptides in ccRCC tumor tissue and on the involvement of these genes in ccRCC-associated pathways and/or processes. To investigate common gene ontology (GO) among the candidate genes, we performed DAVID functional annotation analysis (Fig. 5a) [78–80]. The candidate genes were most significantly enriched in the GO terms “extracellular exosome” and “extracellular matrix.” Significant enrichment was also observed for pathways involved in antigen processing and presentation, immune processes, response to hypoxia, and others. To annotate all 113 candidate genes with their GO-Slim Biological Process terms, we used the GO PANTHER classification system (Fig. 5b) [81, 82].

Since ccRCC is known as a metabolic disease with major alterations in energy and lipid metabolism [50, 83–86], we were further interested in possible metabolic interactions of the candidates. Therefore, tissue metabolites of 30 samples from cohort 1 were assessed by targeted metabolomics using liquid chromatography and flow injection analysis mass spectrometry [50], allowing the absolute quantification of 204 metabolites of eight different molecule classes (acyl/alkylphosphatidylcholines, diacylphosphatidylcholines, lysophosphatidylcholines, acylcarnitines, amino acids, biogenic amines, sphingomyelins, and hydroxysphingomyelins), and 10 metabolites involved in cellular energy metabolism (Additional file 2: Tab. S4). Candidate genes were considered to be correlated with a certain metabolic process or molecule class, if the Pearson correlation coefficients between gene expression and minimum 25% of metabolites of the metabolic group were above 0.3 and significant (*p* < 0.05). In addition, the median correlation coefficient with all metabolites of a group needed to be ≥ 0.3. With the applied criteria, 21 candidates correlated with one up to six different metabolite classes (Fig. 5c). Several of the correlating candidates are known for their role in lipid metabolism, such as the cholesterol and phospholipid transporter ABCA1; the glycolytic enzyme ALDOA, which is also involved in glycogen storage; the sulfotransferase GAL3ST1, the high-density lipoprotein (HDL) scavenger receptor SCARB1; and the stearoyl-CoA desaturase SCD. SCD is an important generator of unsaturated fatty acids, to counteract the cytotoxicity of accumulating saturated fatty acids and improve cell viability, also in ccRCC [87]. The hepatocyte growth factor (HGF) receptor MET, which correlated with different classes of lipid species, is already used as therapeutic target in ccRCC [88].

Finally, we were interested in immune-oncological interactions of candidate genes and therefore investigated the associations of candidate gene expression with the presence of immune-oncological markers. The abundancies of 92 immunological marker proteins in 37 tumor tissues of patient cohort 1 were measured using an immune-oncology panel (Olink Proteomics). The marker proteins were grouped into six biological processes, according to the panel description (Additional file 2: Tab. S5). To determine the associations of candidate gene expression and immune-oncological processes, we performed correlation analyses of candidate gene and marker expression. If a candidate correlated significantly with a Pearson correlation coefficient above 0.3 with more than 25% of process markers, the candidate was considered to be associated with the process. Fourteen of the 113 candidate genes fulfilled the criteria (Fig. 5d). Eleven candidates were associated with chemotaxis, four with the suppression of tumor immunity, four with metabolism and autophagy, three with promotion of tumor immunity, one with vascular and tissue remodeling, and none with apoptosis and cell killing. Note that the same candidate could be associated with more than one biological process.

Taken together, the functional characterization of the candidates with GO annotations, and metabolic and onco-immunological interactions is intended to aid the prioritization of candidate targets and to propose a direction for deeper experimental studies to analyze the molecular functions of candidates and their suitability as drug targets in ccRCC. All gathered information on the candidates of our analysis workflow is summarized in Fig. 5e. A tabular overview of the information can be found in Additional file 2: Tab. S10.

### Peptides of candidate genes activate CD8^+^ T cells in priming assays

**Fig. 6:**
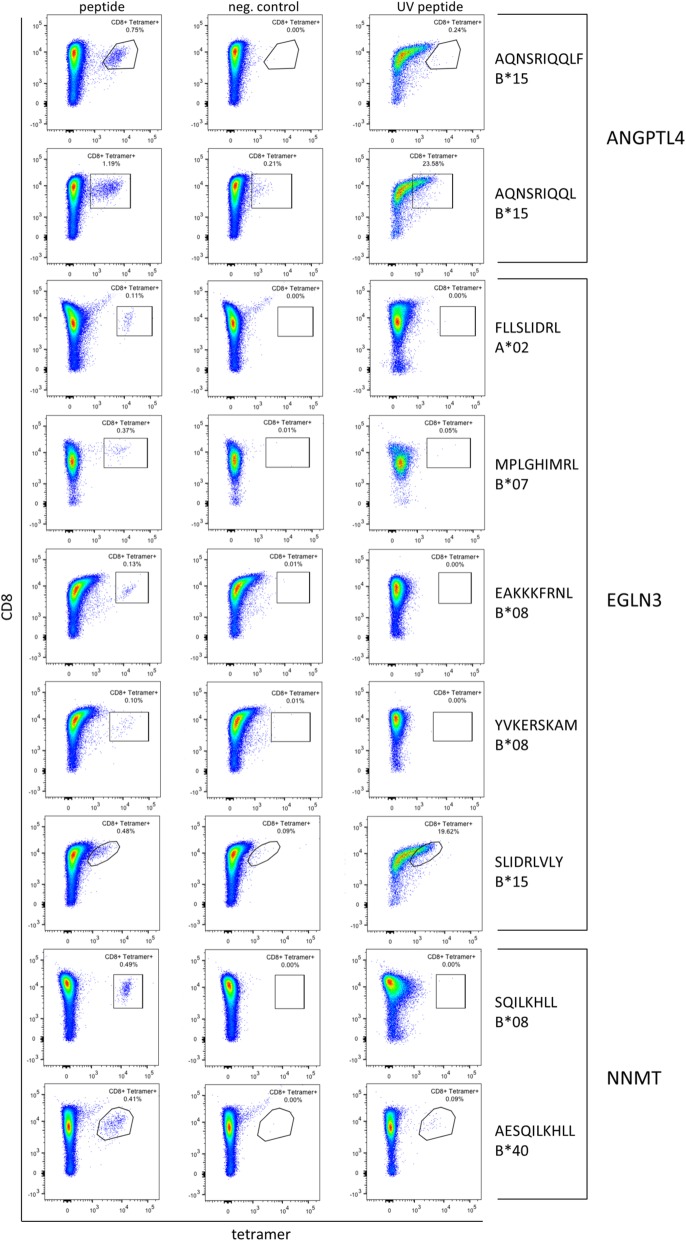
Assessment of immunogenicity of selected candidate peptides. The immunogenicity of 12 peptides from 5 candidate genes was assessed by CD8^+^ T cell priming and tetramer staining assays (Table 2). Left column: tetramer staining of CD8^+^ T cells primed with the indicated ccRCC-specific peptide. Middle column (negative control): ccRCC-specific peptide tetramer staining of CD8^+^ T cells primed with an unrelated, HLA-matched peptide. Right column (UV peptide): tetramer staining of positively primed CD8^+^ T cells with the respective UV-sensitive peptide tetramer

If ccRCC-specific peptides are to be used in cancer vaccines, they need to be able to activate naïve T cells into effector T cells. In the scope of this study, we tested twelve HLA class I peptides from the five candidate genes *EGLN3*, *NNMT*, *ANGPTL4*, *PFKP*, and *P4HA2* for their immunogenic potential in CD8^+^ T cell priming assays (Fig. 6). Thereby the immunogenicity of nine peptides derived from *EGLN3*, *NNMT*, and *ANGPTL4* and restricted to the HLA alleles A*02, B*07, B*08, B*15, and B*40 was proven (Table 2). In combination, the nine immunogenic peptides were detected in 24 patients (43.6%) of patient cohort 1.

**Table 2:** Immunogenicity of candidate gene-derived peptides in CD8^+^ T cell priming assays

Protein	Peptide	HLA restriction^a^	Number of positive tumors (%)	Immunogenic	Positive population
ANGPTL4	AQNSRIQQLF	B*15	4 (7.3)	Yes	0.75%
	AQNSRIQQL	B*15	3 (5.5)	Yes	1.19%
EGLN3	FLLSLIDRL	A*02	9 (16.4)	Yes	0.11%
	MPLGHIMRL	B*07, B*08, B*35, B*51, B*53	8 (14.5)	Yes	0.37%
	EAKKKFRNL	B*08	1 (1.8)	Yes	0.13%
	YVKERSKAM	B*08	1 (1.8)	Yes	0.10%
	SLIDRLVLY	B*15	3 (5.5)	Yes	0.48%
	VQPSYATRY	B*15	3 (5.5)	No	–
NNMT	SQILKHLL	B*08	3 (5.5)	Yes	0.49%
	AESQILKHLL	B*40, B*44	4 (7.3)	Yes	0.41%
P4HA2	AEKELVQSL	B*40, B*41, B*44	5 (9.1)	No	–
PFKP	RSFAGNLNTY	B*15	4 (7.3)	No	–

^a^HLA restrictions for which the immunogenicity of the respective peptide was tested are marked in bold

### Functional analyses of EGLN3 as target for ccRCC therapy

**Fig. 7:**
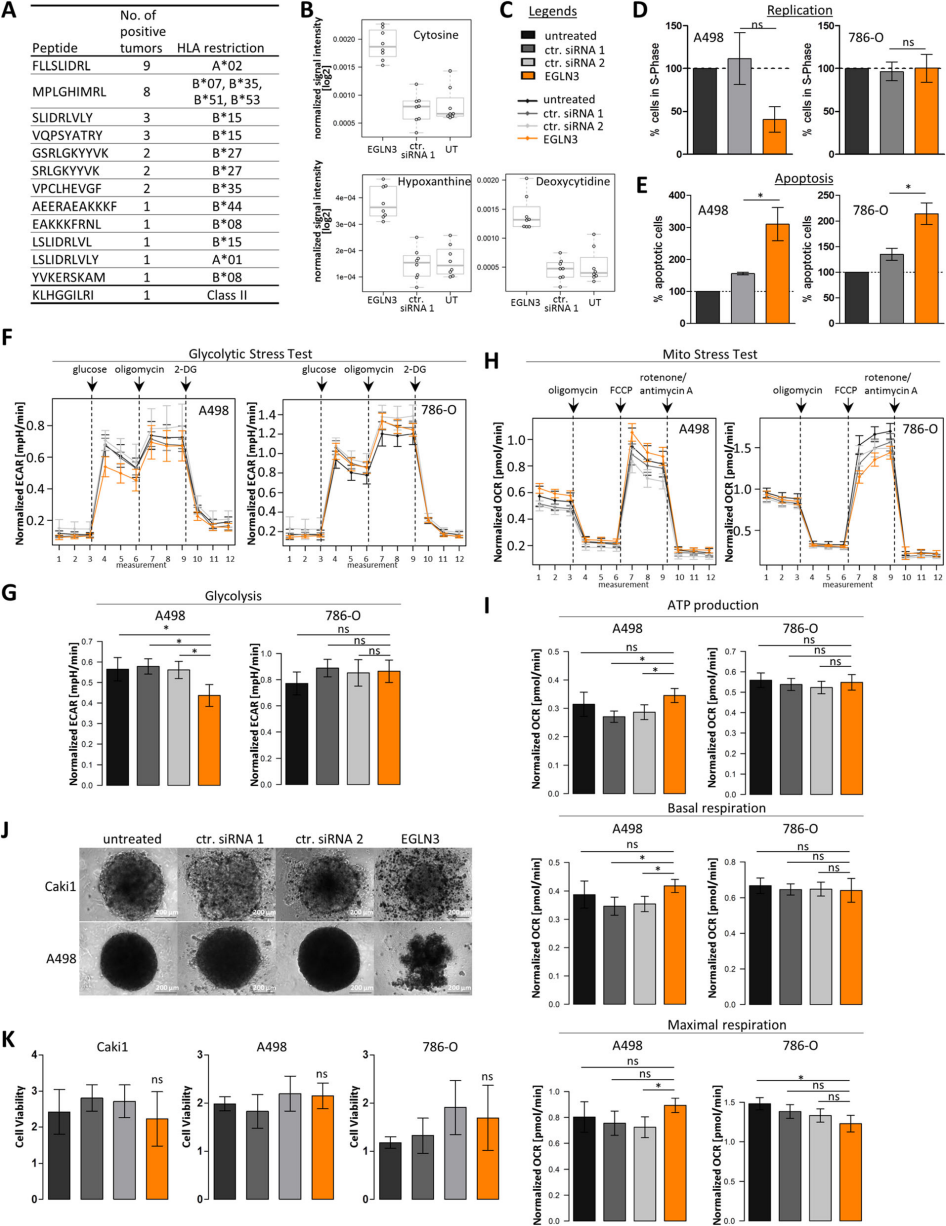
Functional investigation of the candidate target gene *EGLN3*. **a** Tumor-exclusive *EGLN3*-derived peptides detected by HLA ligandomics in patient cohort 1. The number of positive tumors and the HLA restriction of the respective peptides are given. **b** Cellular metabolites regulated by *EGLN3* knockdown (EGLN3) in the 786-O kidney carcinoma cell line (ctr. siRNA 1, cells transfected with the non-targeting siRNA pool 1; UT, untreated cells). Metabolites were identified by untargeted metabolomics analysis. **c** Legend for the graphs in the figure. Untreated, untreated cells; ctr. siRNA 1/2, cells transfected with two different non-targeting siRNA pools; EGLN3, *EGLN3* knockdown cells. **d** Percentage of cells in S-phase. The A498 and 786-O cell lines were used in the experiments. The effect of *EGLN3* knockdown was non-significant (*p* ≥ 0.05). **e** Percentage of apoptotic A498 and 786-O cells. The asterisks mark significant effects with *p* < 0.05. **f** Profiles of extracellular acidification rate (ECAR) in A498 and 786-O cells treated with glucose, oligomycin, and 2-DG (Glycolytic Stress Test, Agilent Technologies). The *x*-axis shows the measurement cycle. **g** Effect on glycolysis in A498 and 786-O cells. The asterisks mark significant differences (*p* < 0.05), whereas ns indicates non-significant differences. **h** Profiles of oxygen consumption in A498 and 786-O cells treated with oligomycin, FCCP, and rotenone/antimycin A (Mito Stress Test, Agilent Technologies). **i** Effects on ATP production, basal respiration, and maximal respiration. The asterisks mark significant differences (*p* < 0.05), whereas ns indicates non-significant differences. **j** Brightfield images of spheroids formed by *EGLN3* knockdown and control cells of the Caki1 and A498 cell lines. **k** Cell viability of Caki1, A498, and 786-O cells assessed by the WST-1 proliferation reagent

The hypoxia inducible factor (HIF) prolyl hydroxylase 3 (*PHD3*; *EGLN3*) was one of the most promising candidate targets of our analysis approach. In total, 13 ccRCC-specific peptides derived from EGLN3 were identified (Fig. 7a), five of which were proven to be immunogenic (Table 2). Interestingly, the presence of EGLN3-derived peptides was associated with higher infiltration of those tumors by CD8^+^ T cells (*p* = 0.061), as determined by GSEA with a signature developed by Rooney et al. [89]. At the same time, however, the tumors were infiltrated by higher numbers of regulatory T cells (*p* = 0.076), indicating active immune suppression (Additional file 2: Fig. S4). Other immune cells, including B cells, macrophages, and natural killer (NK) cells, did not correlate with the presence of EGLN3-derived peptides (Additional file 2: Fig. S4). Supporting the notion of activated immune suppression is the positive correlation of EGLN3-derived peptide presentation and PD1 RNA expression in the tumor tissue (*p* = 0.020). Of note, we assume that PD1 is expressed by tumor-infiltrating lymphocytes within the tissue sample and not by the cancer cells themselves, as previously demonstrated by Giraldo et al. [90]. Concerning regulation, *EGLN3* expression was found to be potentially regulated by DNA methylation in TCGA patient cohort (Additional file 2: Tab. S6), which could be confirmed in patient cohort 1 using MALDI-TOF MS-based quantification of DNA methylation (Additional file 2: Fig. S5). Somatic mutations of *EGLN3* were not detected in TCGA cohort (Fig. 5e). *EGLN3* was not associated with any of the metabolite classes or onco-immunological processes; however, the “cellular response to hypoxia and stress” and the “regulation of HIFα” were functions annotated to *EGLN3* by PANTHER GO complete analysis (data not shown). Analysis of cellular metabolites by untargeted metabolomics furthermore revealed differentially regulated metabolites in *EGLN3* knockdown and control cells. Very prominently, cytosine and deoxycytidine were present in increased abundancies in *EGLN3*-depleted cells (Fig. 7b), in addition to other features representing nucleotide/nucleoside structures and the purine derivate hypoxanthine. Abundance of creatine and pantothenic acid on the other hand was decreased in *EGLN3* knockdown cells. All features that were detected by metabolomics analysis are plotted in Additional file 2: Fig. S6. To assess whether *EGLN3* could represent a drug target in ccRCC, we investigated its function in ccRCC cell culture models. *EGLN3* knockdown decreased cell replication in A498 cells (Fig. 7d) and increased apoptosis in A498 and 786-O cells (Fig. 7e), pointing towards a pro-proliferative and anti-apoptotic function of *EGLN3* in these cell lines. In addition, *EGLN3* knockdown impaired glycolysis in A498 cells, which could be a reason for the decreased cell replication (Fig. 7f, g). Mitochondrial function was also affected by *EGLN3* knockdown; however, effects differed in A498 and 786-O cells. Whereas mitochondrial respiration and ATP production were increased in A498 cells with *EGLN3* depletion, respiration in 786-O cells was decreased (Fig. 7h, i). An interesting phenotype of *EGLN3* knockdown cells was their impaired ability to form stable spheroids, in contrast to untreated or non-targeting siRNA transfected control cells (Fig. 7j). *EGLN3* knockdown did not affect cell viability (Fig. 7k).

## Discussion

### Workflow for the identification of drug targets in ccRCC using multi-omics analyses

Despite targeted therapies, ccRCC is still a cancer with very poor survival rates when tumors have reached advanced or metastatic stages. Currently available treatment options rarely achieve durable responses, resulting in a 5-year survival rate of only 12% for patients with advanced/metastatic disease [2], and further treatment strategies are warranted. Although the mutational load in ccRCC tumors is low, ccRCC is an immunogenic cancer with high numbers of cytolytic tumor-infiltrating lymphocytes (TILs) [59, 89, 91, 92], making it a tumor entity susceptible to immunotherapeutic intervention. Here, we have developed a workflow that integrates tumor immunogenicity with tumor-associated pathways in order to identify target structures for cancer vaccines or ACT therapy. Our approach of comparative HLA ligandome profiling with our unique large dataset of benign tissue samples and adjacent non-tumor tissues and especially the combination with other *-omics* data might enable the selection of suitable targets for peptide-based immunotherapy. HLA ligandomics in tumors and non-tumor tissues was used to detect ccRCC-specific peptides presented to the immune system on the surface of tumor cells. Today, HLA ligandomics is the only method for direct measuring of the HLA peptidome of a cell or tissue, as reliable in silico methods to predict peptide presentation from, for example, transcriptome or proteome data are missing [93, 94]. A recent study demonstrates even feasibility of immunopeptidomics using colorectal cancer organoids, thereby enabling the identification of only cancer cell-specific peptides without impairment of peptides derived from intratumoural stroma or immune cells [95]. The application of similar technologies using organoids derived from renal cell carcinoma in the future may further refine characterization of ccRCC-specific peptides.

However, we have to emphasize that the sensitivity of shotgun mass spectrometry, even in the context of massive technical developments in the last decades [96], is for sure limited since the HLA ligandome is a highly dynamic and complex assembly of peptides. Nevertheless, mass spectrometry-based immunopeptidomics is currently the only unbiased methodology to identify the entirety of naturally processed and presented HLA peptides in primary tissue samples [97]. Importantly, tumor-exclusive peptides may be effective in the treatment of metastatic disease, since peptide profiles have been shown to be preserved between primary tumor tissues and metastatic sites [98]. For ccRCC, the vaccine IMA901, containing 10 ccRCC-associated peptides, has been developed for therapy and undergone successful phase I and II clinical trials, before it failed to show a therapeutic benefit in combination with sunitinib, compared to treatment with sunitinib alone in the consecutive phase III study [99, 100]. Another vaccine containing ccRCC-associated peptides has undergone a phase I/II clinical investigation as adjuvant treatment for patients with advanced disease, showing beneficial effects for overall patient survival [11]. In the here presented study, the mere presence of tumor-exclusive peptides did not correlate with overall survival of patients, which is not surprising considering the immune suppressive microenvironment of established ccRCC tumors [59]. In a recent study, we could show that spontaneous pre-existing T-cell responses against novel defined tumor-associated antigens are correlated with overall patient survival in chronic lymphocytic leukemia patients indicating that not only the presentation of tumor-associated antigens but rather the recognition of these antigens by T cells is important for patient survival [17]. Local and systemic immune suppression might be the reason why cancer vaccination trials in the past did not achieve the expected clinical benefits for patients, although antigen-reactive T cells were detected in some cases [101–103]. In clinical applications, combination of peptides with immune-activating agents, such as checkpoint inhibitors, might therefore be necessary to fully unleash the potential of tumor-associated antigens as target structures.

In our workflow design, we followed the rationale that peptides represent best tumor targets if their source proteins/genes are also involved in tumor pathogenesis. We would expect such peptides to be widely presented throughout the tumor tissue, and delayed resistance development due to peptide loss. Therefore, we performed GSEA based on transcriptome data with selected signature collections from the Molecular Signatures Database (MSigDB) and additional immunologic signatures from a recent publication [43], to identify ccRCC-enriched molecular pathways. Single cell sequencing data of the kidney proximal tubule cells, representing the origin of ccRCC tumors, served as an additional control to exclude pathways that are associated with the region of tumor origin in the nephron, rather than with tumor development and progression. Expression data from proximal tubules were selected because we previously showed that tumor aggressiveness in ccRCC is correlated with the level of divergence from its cell of origin within the nephron region [32, 62]. The combined analysis of ccRCC-associated peptides and pathways in the same tumor tissues yielded 185 potential target proteins/genes, of which 113 could be confirmed by additional data analysis of an independent patient cohort from TCGA project. Taken together, our candidate list represents an unbiased pre-selection of genes with target structures on ccRCC tumor cells, in the form of HLA-bound peptides, and with potential involvement in tumor pathophysiology.

### The drug target list—characterization of candidates and targeting

First, before candidate gene-derived peptides can be used in peptide vaccines, their immunogenicity (meaning their ability to activate patient immune cells) needs to be assessed. In the scope of this study, we tested the immunogenicity of twelve selected peptides from our candidates in T cell priming assays. We selected such peptides for immunogenicity screenings that were presented on ≥ 3 patients and are restricted to HLA-A*02, HLA-B*07, HLA-B*08, HLA-B*15, HLA-B*40, and HLA-B*44. Nine of the tested peptides were able to induce CD8^+^ T cells and, hence, represent promising vaccine candidates. If a candidate gene does not generate immunogenic peptides, or if immunologic targeting is not feasible for any reasons, direct targeting of the candidate genes/proteins could represent an alternative therapeutic option. In fact, selective inhibitors of, for example, the candidates *NNMT* [104–106] and *SLC16A3* [107] are available. The candidate VEGFA is the target structure of bevacizumab, a monoclonal antibody that is already approved for ccRCC therapy [108, 109].

To investigate the potential of the candidates as drug targets, we integrated further -omics data (e.g., genomic, metabolomic, and proteomic data) (see Fig. 5 and Additional file 2: Tab. S10). For instance, in addition to peptide vaccines or direct targeting, epigenetic modulation of the candidates could provide another therapeutic option. We identified decreased DNA methylation in the gene regions of several candidate targets as potential mechanism of upregulation in tumors. In ccRCC, DNA methylation has been shown to be greatly altered [63], with DNA methylation patterns being preserved in metastases [31]. Thus, in the future, site-specific DNA methylation [110, 111] could represent a strategy to inhibit the expression of affected candidates and could be beneficial also for patients with metastatic disease. In addition, demethylating agents like decitabine are used in clinical practice, e.g., for treatment of myelodysplastic syndromes. These agents are known to induce the expression of tumor suppressor genes and/or induce global hypomethylation in tumor cells. We could already show that treatment of RCC cells with the hypomethylating agent decitabine resulted in an upregulation of important drug transporters thereby influencing cisplatin treatment effect [31]. In addition, the immune-modulatory effect of demethylating agents is increasingly recognized. Several in vitro and in vivo studies indicate that treatment with demethylating agents leads to an upregulation of immunogenic molecules such as cancer testis antigens [112, 113] and neo-antigens [66] in tumor cells. Therefore, demethylation induced by demethylating agents in RCC might result in restoration or induction of HLA-presented antigen targets. As shown in Additional file 2: Fig. S7, our selected candidates are generally induced in the tumor tissue compared to normal kidney tissue, but inter-individual variability in expression is still observed, and subsequently, combination therapies with demethylating agents might induce stable expression of vaccine candidates. As DNA demethylating agents have been associated with increased antigen processing and presentation [65], a combination therapy of epigenetic drugs with peptide vaccination might overcome the limitations of both as single therapies.

Moreover, somatic mutations in candidate genes could be exploited in individualized treatment plans within the concept of personalized medicine. Neo-antigens arising from somatic mutations in tumor tissues are considered promising targets in cancer therapy, since they are not expressed on any normal tissue, avoiding immune-tolerance as well as off-target side effects. Although personalized peptide vaccines containing neo-epitopes are currently tested in patients, with promising results in melanoma [114] and glioblastoma [115, 116], the identification of tumor-presented neo-epitopes is still a challenge [117, 118]. Moreover, most somatic mutations are present only in subpopulations of the bulk tumor tissue, contributing to the observed intratumor heterogeneity in ccRCC [119–121], complicating vaccination against mutated neo-antigens in ccRCCs. Therefore, our study focused on the identification of non-mutated self-peptides presented by at least 3 patients. Analyses of somatic variation within the identified candidate genes indicated that somatic point mutations or small indel mutations occur only in single cases. Thus, alteration by somatic mutations can be excluded as relevant confounder for the candidate-derived peptide presentation.

### Functional relevance of candidates in ccRCC

Several of our candidate genes are known to be closely connected with ccRCC pathogenesis. Among them is the angiogenic mediator *VEGFA*, the lactate transporter *SLC16A3/MCT4*, or the hypoxia-induced genes *ANGPTL4*, *PLIN2*, and *EGLN3*. To provide functional information for all candidates of our workflow, we performed GO analysis. Significant enrichment was observed for terms related to extracellular matrix, antigen processing and presentation, immune processes, and response to hypoxia. In contrast to a previous study by Klatt et al. [122] where network analysis with ccRCC HLA ligand source proteins was performed, our candidate list was pre-filtered for tumor exclusivity. Nevertheless, hypoxic signaling and extracellular matrix organization were top hits in both studies, underlining the importance of those processes in ccRCC carcinogenesis. In addition, to reveal potentially novel functions of the candidates, we analyzed the relationships between candidate gene expression, tumor metabolism, and the presence of onco-immunological markers in representative matched tumor samples of cohort 1 for which enough tissue material was available after ligandomics, transcriptomics, and genetic analyses. Especially, the candidates *ABCA1*, *MET*, and *SCD* were entangled with tumor metabolism, while *FCGR1A* and *ITGB2* showed the widest interactions with immunological processes. GO annotation and metabolic and immune-oncological interactions of candidates were not used for further candidate filtering but rather intended to provide additional information which may guide candidate selection and design of in-depth analysis of their function and suitability as drug target in ccRCC. In this study, we focused on the functional analysis of the hypoxia-regulated gene *EGLN3*. Based on our ligandomics analysis, 13 EGLN3-derived peptides were ccRCC-specifically presented, two of which were among the most frequently presented peptides. Five out of six peptides included in the T cell priming assays were determined to be immunogenic and represent promising candidates to be included in a peptide vaccine for ccRCC patients. The presence of EGLN3-derived peptides was furthermore associated with higher expression of PD1, and CD8^+^ and regulatory T cell infiltration, indicating interactions with the immune system. Thus, we decided to further investigate the function of *EGLN3* in ccRCC and performed experiments in different ccRCC cell culture models. Untargeted metabolomics analysis in 786-O kidney carcinoma cells revealed an impact of *EGLN3* depletion on the abundance of different cellular nucleotide components and nucleotide-related structures, such as cytosine and deoxycytidine. The accumulation of the nucleotide-related molecules in *EGLN3* knockdown cells could be the consequence of increased synthesis/salvage, decreased usage, or increased DNA or RNA degradation. In further analyses, we showed that *EGLN3* acts pro-proliferative and anti-apoptotic, and hence, could contribute to ccRCC malignant transformation and progression. Both decreased proliferation and enhanced apoptotic signaling in *EGLN3*-depleted cells might result in the accumulation of nucleotide-related molecules that we observed in the untargeted metabolomics analysis. Furthermore, and in agreement with a recent publication [123], we measured impaired glycolysis in *EGLN3*-depleted cells, pointing to a glycolysis-promoting function of *EGLN3*. At the same time, cell respiration, a direct marker for mitochondrial oxidative phosphorylation, was enhanced in *EGLN3*-depleted cells, most likely to compensate for the loss in glycolytic activity. Finally, we found *EGLN3* depletion to impair cells’ ability to form stable spheroids. The mechanism underlying this phenotype is currently unknown, as is the potential consequence for in vivo tumor development, formation, and progression. So far, various functions have been proposed for *EGLN3* in cancer, ranging from pro-apoptotic [124–126] to growth-promoting [127, 128] and metabolic functions [129–131]. The variety of identified functions, as well as the cell type-dependent effects of *EGLN3* knockdown we observed in our experiments, point towards a complex interaction network of *EGLN3*. It seems possible that the function of *EGLN3* depends on co-expressed factors, which might deviate in the different cell culture models. Thus, one limitation of our study is that other model systems that better resemble the in vivo tumor situation, such as microtumors or cancer organoids, might be more appropriate to understand the function of *EGLN3* in ccRCC and to assess its feasibility as a drug target.

Our study exemplarily highlights that comprehensive integration of different *-omics* technologies, including especially HLA ligandomics, provides not only the basis for the identification of novel immunologic targets and further insight into their regulation, but offers the potential for identification of novel combination therapies.

## Conclusions

The HLA class I- and class II-presented peptides identified in this study represent immunologic targets that could complement ccRCC therapy in the future. Their frequent and tumor-specific presentation, as well as the involvement of their source genes in tumor pathogenesis, renders them eligible as components of cancer vaccines or targets of ACT. In both cases, further validation of their immunogenicity and considerations concerning the appropriate form (long vs short peptides, combined class I and II epitopes) and formulation (e.g., vaccine adjuvants) are, however, mandatory. Since ccRCC is an immunogenic cancer with a low mutational frequency and a high mortality in advanced disease, tumor-specific over-presented self-peptides could increase response rates and benefits for patients, especially in combination with immune checkpoint inhibition.

## Supplementary information

**Additional file 1. **Contains supplementary table Tab. S1: HLA class I and II restricted peptides from the 113 candidate genes identified by HLA ligandomics in patient cohort 1 (*n* = 55).

**Additional file 2.** Contains supplementary tables Tab. S2-S10 and supplementary figures Fig. S1-S7.

## Data Availability

The accession number for genome-wide data generated for primary ccRCC at the European Genome-phenome Archive (EGA) (www.ebi.ac.uk/ega/home), which is hosted by the EBI and the CRG, is EGAS00001001176 [132]. The mass spectrometry proteomics data have been deposited to the ProteomeXchange Consortium via the PRIDE [133] partner repository with the dataset identifier PXD017149 [134]. All other data of this study are available from the corresponding author upon reasonable request.
