# Supplementary material for: Integrative -omics and HLA-ligandomics analysis to identify novel drug targets for ccRCC immunotherapy
Source: Genome Med. 2020 Mar 30;12:32. doi: 10.1186/s13073-020-00731-8 (PMC7106651; doi:10.1186/s13073-020-00731-8)
Supplement: Supplementary file 2 — Additional file 2. Contains supplementary tables Tab. S2-S10 and supplementary figures Fig. S1-S7. [file 13073_2020_731_MOESM2_ESM.pdf]

Additional file 2: Reustle et al.

Supplementary Tables:

Tab. S2: HLA typing of patient cohort 1 and healthy donors for PBMC isolation.
Tab. S3: Gene signatures for gene set enrichment analysis (GSEA).
Tab. S4: Metabolites and metabolite classes included in the targeted metabolomics analysis of cohort 1.
Tab. S5: Immuno-oncological marker proteins and associated biological processes.
Tab. S6: Differentially methylated CpG sites in candidate gene regions.
Tab. S7: Somatic mutations of candidate genes in the TCGA patient cohort.
Tab. S8: Shared somatic variants in the TCGA patient cohort.
Tab. S9: Coding variants in the candidate genes TSC2, RB1, and MET in patient cohort 1.
Tab. S10: Overview of candidate genes.

Supplementary Figures:

Fig. S1: Peptide presentation and overall patient survival in patient cohort 1.
Fig. S2: DNA methylation profiles of differentially methylated genes in the TCGA patient cohort.
Fig. S3: Somatic variants in candidate genes in 392 samples of the TCGA patient cohort.
Fig. S4: EGLN3-derived peptides and tumor immune infiltrate in patient cohort 1.
Fig. S5: DNA methylation of the EGLN3 gene region.
Fig. S6: Differentially regulated metabolites in EGLN3 knockdown 786-O cells.
Fig. S7: Heatmap of gene expression of the 113 candidates in non-tumor and ccRCC tissue of the TCGA patient cohort.

Tab. S2: HLA typing of patient cohort 1 and healthy donors for PBMC isolation. The median numbers of class I and II HLA ligands detected in ccRCC and adjacent non-tumor tissues are also given in the table. In the case of healthy donors, tested HLA alleles are underlined.

	**HLA class I**		**HLA class II**	
ccRCC: median nr. of peptides per patient (range)	1457 (121-4934)		636 (0-4924)	
Adjacent non-tumor tissue: median nr. of peptides per patient (range)	768 (40-2287)		442 (0-2055)	
	**Allele**	**Nr. of patients**	**Allele**	**Nr. of patients**
Cohort 1 (n=55)	A*01	16	Not available[a]	3
	A*02	22	DPB1*02	3
	A*03	12	DPB1*03	4
	A*11	9	DPB1*04	9
	A*23	1	DPB1*06	1
	A*24	11	DPB1*09	1
	A*25	1	DPB1*11	1
	A*26	4	DPB1*13	1
	A*29	4	DPB1*14	1
	A*30	3	DQA1*01	31
	A*31	4	DQA1*02	12
	A*32	2	DQA1*03	5
	A*33	1	DQA1*04	2
	A*66	1	DQA1*05	19
	A*68	6	DQB1*02	21
	B*07	11	DQB1*03	28
	B*08	10	DQB1*04	3
	B*13	3	DQB1*05	19
	B*14	2	DQB1*06	23
	B*15	7	DRB1*01	12
	B*18	5	DRB1*03	11
	B*27	6	DRB1*04	9
	B*35	11	DRB1*07	15
	B*37	2	DRB1*08	3
	B*38	3	DRB1*09	1
	B*39	2	DRB1*11	14
	B*40	6	DRB1*12	1
	B*41	1	DRB1*13	17
	B*44	14	DRB1*14	5
	B*49	1	DRB1*15	8
	B*50	2	DRB1*16	4
	B*51	9	DRB3	1
	B*52	2	DRB3*01	4
	B*53	3	DRB3*02	7
	B*55	2	DRB3*03	4

	B*57	2	DRB4	2
	B*58	2	DRB4*01	4
	C*01	3		
	C*02	11		
	C*03	10		
	C*04	11		
	C*05	6		
	C*06	7		
	C*07	28		
	C*08	2		
	C*12	6		
	C*14	1		
	C*15	5		
	C*16	4		
	C*17	1		
Healthy donors (n=6)	A*01	3		
	A*02	5		
	A*03	2		
	A*29	1		
	B*07	1		
	B*08	2		
	B*15	2		
	B*35	1		
	B*40	1		
	B*41	1		
	B*44	2		
	B*51	2		

[a] HLA class II alleles could not be determined

Tab. S3: Gene signatures for gene set enrichment analysis (GSEA). Shown are only those that were enriched in at least one of the cohorts.

Signature	Collection	Source	Description (MSigDB)	Enriched in cohort	In final set
BCAT_BILD_ET_AL_UP	C6 Oncogenic gene sets	MSigDB	Genes up-regulated in primary epithelial breast cancer cell culture over-expressing activated CTNNB1 [GeneID=1499] gene.	TCGA	no
CAMP_UP.V1_DN	C6 Oncogenic gene sets	MSigDB	Genes down-regulated in primary thyrocyte cultures in response to cAMP signaling pathway activation by thyrotropin (TSH).	TCGA	no
CAMP_UP.V1_UP	C6 Oncogenic gene sets	MSigDB	Genes up-regulated in primary thyrocyte cultures in response to cAMP signaling pathway activation by thyrotropin (TSH).	cohort 1, TCGA	yes
CORDENONSI_YAP_CONSERVED_SIGNATURE	C6 Oncogenic gene sets	MSigDB	YAP conserved signature.	cohort 1, TCGA	yes
CSR_EARLY_UP.V1_UP	C6 Oncogenic gene sets	MSigDB	Genes up-regulated in early serum response of CRL 2091 cells (foreskin fibroblasts).	TCGA	no
E2F1_UP.V1_DN	C6 Oncogenic gene sets	MSigDB	Genes down-regulated in mouse fibroblasts over-expressing E2F1 [GeneID=1869] gene.	TCGA	no
EGFR_UP.V1_UP	C6 Oncogenic gene sets	MSigDB	Genes up-regulated in MCF-7 cells (breast cancer) positive for ESR1 [GeneID=2099] and engineered to express ligand-activatable EGFR [GeneID=1956].	TCGA	no
EIF4E_DN	C6 Oncogenic gene sets	MSigDB	Genes down-regulated in HMEC cells (primary mammary epithelium) upon over-expression of EIF4E [GeneID=1977] gene.	TCGA	no
GLI1_UP.V1_UP	C6 Oncogenic gene sets	MSigDB	Genes up-regulated in RK3E cells (kidney epithelium) over-expressing GLI1 [GeneID=2735].	TCGA	no
HINATA_NFKB_MATRIX	C6 Oncogenic gene sets	MSigDB	Matrix, adhesion or cytoskeleton genes induced by NF-kappaB in primary keratinocytes and fibroblasts.	cohort 1, TCGA	yes
HOXA9_DN.V1_UP	C6 Oncogenic gene sets	MSigDB	Genes up-regulated in MOLM-14 cells (AML) with knockdown of HOXA9 [GeneID=3205] gene by RNAi vs controls.	TCGA	no
RB_DN.V1_DN	C6 Oncogenic gene sets	MSigDB	Genes down-regulated in primary keratinocytes from RB1 [GeneID=5925] skin specific knockout mice.	TCGA	no
RB_P107_DN.V1_DN	C6 Oncogenic gene sets	MSigDB	Genes down-regulated in primary keratinocytes from RB1 and RBL1 [GeneID=5925][GeneID=5933] skin specific knockout mice.	TCGA	no
RB_P130_DN.V1_DN	C6 Oncogenic gene sets	MSigDB	Genes down-regulated in primary keratinocytes from RB1 and RBL2 [GeneID=5925][GeneID=5934] skin specific knockout mice.	cohort 1, TCGA	yes
SIRNA_EIF4GI_UP	C6 Oncogenic gene sets	MSigDB	Genes up-regulated in MCF10A cells vs knockdown of EIF4G1 [GeneID=1981] gene by RNAi.	cohort 1, TCGA	yes

TBK1.DF_DN	C6 Oncogenic gene sets	MSigDB	Genes down-regulated in epithelial lung cancer cell lines upon over-expression of an oncogenic form of KRAS [GeneID=3845] gene and knockdown of TBK1 [GeneID=29110] gene by RNAi.	TCGA	no
TBK1.DF_UP	C6 Oncogenic gene sets	MSigDB	Genes down-regulated in epithelial lung cancer cell lines upon over-expression of an oncogenic form of KRAS [GeneID=3845] gene and knockdown of TBK1 [GeneID=29110] gene by RNAi.	TCGA	no
TBK1.DN.48HRS_DN	C6 Oncogenic gene sets	MSigDB	Genes up-regulated in epithelial lung cancer cell lines upon over-expression of an oncogenic form of KRAS [GeneID=3845] gene and knockdown of TBK1 [GeneID=29110] gene by RNAi.	TCGA	no
TBK1.DN.48HRS_UP	C6 Oncogenic gene sets	MSigDB	Genes up-regulated in epithelial lung cancer cell lines upon over-expression of an oncogenic form of KRAS [GeneID=3845] gene and knockdown of TBK1 [GeneID=29110] gene by RNAi.	TCGA	no
YAP1_UP	C6 Oncogenic gene sets	MSigDB	Genes up-regulated in MCF10A cells (breast cancer) over-expressing YAP1 [GeneID=10413]gene.	TCGA	no
HALLMARK_ADIPOGENESIS	H Hallmark gene sets	MSigDB	Genes up-regulated during adipocyte differentiation (adipogenesis).	cohort 1, TCGA	yes
HALLMARK_ANDROGEN_RESPONSE	H Hallmark gene sets	MSigDB	Genes defining response to androgens.	cohort 1, TCGA	yes
HALLMARK_ANGIOGENESIS	H Hallmark gene sets	MSigDB	Genes up-regulated during formation of blood vessels (angiogenesis).	cohort 1, TCGA	yes
HALLMARK_APOPTOSIS	H Hallmark gene sets	MSigDB	Genes mediating programmed cell death (apoptosis) by activation of caspases.	cohort 1, TCGA	yes
HALLMARK_CHOLESTEROL_HOMEOSTASIS	H Hallmark gene sets	MSigDB	Genes involved in cholesterol homeostasis.	cohort 1, TCGA	yes
HALLMARK_COMPLEMENT	H Hallmark gene sets	MSigDB	Genes encoding components of the complement system, which is part of the innate immune system.	TCGA	no
HALLMARK_DNA_REPAIR	H Hallmark gene sets	MSigDB	Genes involved in DNA repair.	TCGA	no
HALLMARK_EPITHELIAL_MESENCHYMAL_TRANSITION	H Hallmark gene sets	MSigDB	Genes defining epithelial-mesenchymal transition, as in wound healing, fibrosis and metastasis.	cohort 1, TCGA	yes
HALLMARK_FATTY_ACID_METABOLISM	H Hallmark gene sets	MSigDB	Genes encoding proteins involved in metabolism of fatty acids.	TCGA	no
HALLMARK_GLYCOLYSIS	H Hallmark gene sets	MSigDB	Genes encoding proteins involved in glycolysis and gluconeogenesis.	cohort 1, TCGA	yes
HALLMARK_HYPOXIA	H Hallmark gene sets	MSigDB	Genes up-regulated in response to low oxygen levels (hypoxia).	cohort 1, TCGA	yes
HALLMARK_IL2_STAT5_SIGNAL	H Hallmark gene sets	MSigDB	Genes up-regulated by STAT5 in response to IL2 stimulation.	TCGA	no

ING					
HALLMARK_INTERFERON_ALPHA_RESPONSE	H Hallmark gene sets	MSigDB	Genes up-regulated in response to alpha interferon proteins.	cohort 1, TCGA	yes
HALLMARK_INTERFERON_GAMMA_RESPONSE	H Hallmark gene sets	MSigDB	Genes up-regulated in response to IFNG [GeneID=3458].	cohort 1, TCGA	yes
HALLMARK_MITOTIC_SPINDLE	H Hallmark gene sets	MSigDB	Genes important for mitotic spindle assembly.	cohort 1	no
HALLMARK_MTORC1_SIGNALING	H Hallmark gene sets	MSigDB	Genes up-regulated through activation of mTORC1 complex.	cohort 1, TCGA	yes
HALLMARK_MYC_TARGETS_V1	H Hallmark gene sets	MSigDB	A subgroup of genes regulated by MYC - version 1 (v1).	cohort 1, TCGA	yes
HALLMARK_MYC_TARGETS_V2	H Hallmark gene sets	MSigDB	A subgroup of genes regulated by MYC - version 2 (v2).	TCGA	no
HALLMARK_NOTCH_SIGNALING	H Hallmark gene sets	MSigDB	Genes up-regulated by activation of Notch signaling.	cohort 1, TCGA	yes
HALLMARK_OXIDATIVE_PHOSPHORYLATION	H Hallmark gene sets	MSigDB	Genes encoding proteins involved in oxidative phosphorylation.	cohort 1, ProxTub, TCGA	no
HALLMARK_P53_PATHWAY	H Hallmark gene sets	MSigDB	Genes involved in p53 pathways and networks.	cohort 1, TCGA	yes
HALLMARK_PEROXISOME	H Hallmark gene sets	MSigDB	Genes encoding components of peroxisome.	TCGA	no
HALLMARK_PI3K_AKT_MTOR_SIGNALING	H Hallmark gene sets	MSigDB	Genes up-regulated by activation of the PI3K/AKT/mTOR pathway.	cohort 1, TCGA	yes
HALLMARK_PROTEIN_SECRETION	H Hallmark gene sets	MSigDB	Genes involved in protein secretion pathway.	cohort 1, TCGA	yes
HALLMARK_REACTIVE_OXIGEN_SPECIES_PATHWAY	H Hallmark gene sets	MSigDB	Genes up-regulated by reactive oxigen species (ROS).	cohort 1, ProxTub, TCGA	no
HALLMARK_TGF_BETA_SIGNALING	H Hallmark gene sets	MSigDB	Genes up-regulated in response to TGFB1 [GeneID=7040].	cohort 1, TCGA	yes
HALLMARK_TNFA_SIGNALING_VIA_NFKB	H Hallmark gene sets	MSigDB	Genes regulated by NF-kB in response to TNF [GeneID=7124].	cohort 1, TCGA	yes
HALLMARK_UNFOLDED_PROTEIN_RESPONSE	H Hallmark gene sets	MSigDB	Genes up-regulated during unfolded protein response, a cellular stress response related to the endoplasmic reticulum.	cohort 1, TCGA	yes
HALLMARK_UV_RESPONSE_DN	H Hallmark gene sets	MSigDB	Genes down-regulated in response to ultraviolet (UV) radiation.	cohort 1, TCGA	yes
HALLMARK_UV_RESPONSE_UP	H Hallmark gene sets	MSigDB	Genes up-regulated in response to ultraviolet (UV) radiation.	TCGA	no
BIOCARTA_CDC42RAC_PATHWAY	C2 Biocarta	MSigDB	Role of PI3K subunit p85 in regulation of Actin Organization and Cell Migration	TCGA	no
BIOCARTA_EIF_PATHWAY	C2 Biocarta	MSigDB	Eukaryotic protein translation	TCGA	no
BIOCARTA_ETC_PATHWAY	C2 Biocarta	MSigDB	Electron Transport Reaction in Mitochondria	TCGA	no
BIOCARTA_GLYCOLYSIS_PATHWAY	C2 Biocarta	MSigDB	Glycolysis Pathway	TCGA	no
BIOCARTA_KREB_PATHWAY	C2 Biocarta	MSigDB	The Citric Acid Cycle	TCGA	no
BIOCARTA_PROTEASOME_PATHWAY	C2 Biocarta	MSigDB	Proteasome Complex	TCGA	no

BIOCARTA_SALMONELLA_PATHWAY	C2 Biocarta	MSigDB	How does salmonella hijack a cell	cohort 1, TCGA	yes
BIOCARTA_VDR_PATHWAY	C2 Biocarta	MSigDB	Control of Gene Expression by Vitamin D Receptor	cohort 1	no
REACTOME_3_UTR_MEDIATED_TRANSLATIONAL_REGULATION	C2 Reactome	MSigDB	Genes involved in 3' -UTR-mediated translational regulation	TCGA	no
REACTOME_ACTIVATION_OF_THE_MRNA_UPON_BINDING_OF_THE_CAP_BINDING_COMPLEX_AND_EIFS_AND_SUBSEQUENT_BINDING_TO_43S	C2 Reactome	MSigDB	Genes involved in Activation of the mRNA upon binding of the cap-binding complex and eIFs, and subsequent binding to 43S	TCGA	no
REACTOME_COPI_MEDIATED_TRANSPORT	C2 Reactome	MSigDB	Genes involved in COPI Mediated Transport	cohort 1, TCGA	yes
REACTOME_ENDOSOMAL_VACUOLAR_PATHWAY	C2 Reactome	MSigDB	Genes involved in Endosomal/Vacuolar pathway	TCGA	no
REACTOME_FORMATION_OF_ATP_BY_CHEMIOSMOTIC_COUPLING	C2 Reactome	MSigDB	Genes involved in Formation of ATP by chemiosmotic coupling	TCGA	no
REACTOME_FORMATION_OF_THE_TERNARY_COMPLEX_AND_SUBSEQUENTLY_THE_43S_COMPLEX	C2 Reactome	MSigDB	Genes involved in Formation of the ternary complex, and subsequently, the 43S complex	TCGA	no
REACTOME_INFLUENZA_VIRAL_RNA_TRANSCRIPTION_AND_REPLICATION	C2 Reactome	MSigDB	Genes involved in Influenza Viral RNA Transcription and Replication	TCGA	no
REACTOME_NONSENSE_MEDIATED_DECAY_ENHANCED_BY_THE_EXON_JUNCTION_COMPLEX	C2 Reactome	MSigDB	Genes involved in Nonsense Mediated Decay Enhanced by the Exon Junction Complex	TCGA	no
REACTOME_PEPTIDE_CHAIN_ELONGATION	C2 Reactome	MSigDB	Genes involved in Peptide chain elongation	ProxTub, TCGA	no
REACTOME_SRP_DEPENDENT_COTRANSLATIONAL_PROTEIN_TARGETING_TO_MEMBRANE	C2 Reactome	MSigDB	Genes involved in SRP-dependent cotranslational protein targeting to membrane	TCGA	no
REACTOME_TRANSLATION	C2 Reactome	MSigDB	Genes involved in Translation	TCGA	no
KEGG_RIBOSOME	C2 KEGG	MSigDB	Ribosome	ProxTub, TCGA	no
GCM_ACTG1	C4 Computational gene sets	MSigDB	Neighborhood of ACTG1	TCGA	no
GCM_ANP32B	C4 Computational gene sets	MSigDB	Neighborhood of ANP32B	TCGA	no
GCM_APEX1	C4 Computational gene sets	MSigDB	Neighborhood of APEX1	TCGA	no
GCM_CBFB	C4 Computational gene sets	MSigDB	Neighborhood of CBFB	TCGA	no

GCM_CSNK2B	C4 Computational gene sets	MSigDB	Neighborhood of CSNK2B	cohort 1, TCGA	yes
GCM_DDX5	C4 Computational gene sets	MSigDB	Neighborhood of DDX5	TCGA	no
GCM_HDAC1	C4 Computational gene sets	MSigDB	Neighborhood of HDAC1	cohort 1, TCGA	yes
GCM_MSN	C4 Computational gene sets	MSigDB	Neighborhood of MSN	cohort 1, TCGA	yes
GCM_NPM1	C4 Computational gene sets	MSigDB	Neighborhood of NPM1	TCGA	no
GCM_PFN1	C4 Computational gene sets	MSigDB	Neighborhood of PFN1	cohort 1, TCGA	yes
GCM_PPP1CC	C4 Computational gene sets	MSigDB	Neighborhood of PPP1CC	TCGA	no
GCM_PSME1	C4 Computational gene sets	MSigDB	Neighborhood of PSME1	TCGA	no
GCM_RAD21	C4 Computational gene sets	MSigDB	Neighborhood of RAD21	cohort 1, TCGA	yes
GCM_RAF1	C4 Computational gene sets	MSigDB	Neighborhood of RAF1	cohort 1	no
GCM_TPT1	C4 Computational gene sets	MSigDB	Neighborhood of TPT1	cohort 1, TCGA	yes
GNF2_DAP3	C4 Computational gene sets	MSigDB	Neighborhood of DAP3	TCGA	no
GNF2_EIF3S6	C4 Computational gene sets	MSigDB	Neighborhood of EIF3S6	TCGA	no
GNF2_FBL	C4 Computational gene sets	MSigDB	Neighborhood of FBL	TCGA	no
GNF2_GLTSCR2	C4 Computational gene sets	MSigDB	Neighborhood of GLTSCR2	TCGA	no
GNF2_MBD4	C4 Computational gene sets	MSigDB	Neighborhood of MBD4	TCGA	no
GNF2_MSN	C4 Computational gene sets	MSigDB	Neighborhood of MSN	cohort 1	no
GNF2_ST13	C4 Computational gene sets	MSigDB	Neighborhood of ST13	TCGA	no
GNF2_TPT1	C4 Computational gene sets	MSigDB	Neighborhood of TPT1	ProxTub, TCGA	no
GNF2_UBE2I	C4 Computational gene sets	MSigDB	Neighborhood of UBE2I	TCGA	no
MODULE_103	C4 Computational gene sets	MSigDB	Genes in the cancer module 103.	TCGA	no
MODULE_114	C4 Computational gene sets	MSigDB	Protein biosynthesis and ribosomes.	TCGA	no
MODULE_115	C4 Computational gene sets	MSigDB	Genes in the cancer module 115.	TCGA	no
MODULE_116	C4 Computational gene sets	MSigDB	Genes in the cancer module 116.	TCGA	no
MODULE_143	C4 Computational gene sets	MSigDB	Genes in the cancer module 143.	TCGA	no
MODULE_149	C4 Computational gene sets	MSigDB	Translation factors.	TCGA	no
MODULE_150	C4 Computational gene sets	MSigDB	Translation elongation.	cohort 1	no
MODULE_151	C4 Computational gene sets	MSigDB	Genes in the cancer module 151.	TCGA	no
MODULE_155	C4 Computational gene sets	MSigDB	Genes in the cancer module 155.	TCGA	no
MODULE_22	C4 Computational gene sets	MSigDB	Genes in the cancer module 22.	TCGA	no
MODULE_245	C4 Computational gene sets	MSigDB	Genes in the cancer module 245.	TCGA	no
MODULE_29	C4 Computational gene sets	MSigDB	Genes in the cancer module 29.	cohort 1, ProxTub, TCGA	no
MODULE_293	C4 Computational gene sets	MSigDB	Genes in the cancer module 293.	cohort 1, TCGA	yes
MODULE_299	C4 Computational gene sets	MSigDB	Genes in the cancer module 299.	TCGA	no
MODULE_306	C4 Computational gene sets	MSigDB	Glycolysis and TCA cycle.	TCGA	no
MODULE_307	C4 Computational gene sets	MSigDB	Oxidative phosphorylation (COX and ATPases).	TCGA	no
MODULE_355	C4 Computational gene sets	MSigDB	Protein folding.	TCGA	no
MODULE_414	C4 Computational gene sets	MSigDB	Genes in the cancer module 414.	TCGA	no

MODULE_429	C4 Computational gene sets	MSigDB	Genes in the cancer module 429.	TCGA	no
MODULE_50	C4 Computational gene sets	MSigDB	Genes in the cancer module 50.	TCGA	no
MODULE_62	C4 Computational gene sets	MSigDB	Genes in the cancer module 62.	TCGA	no
MODULE_81	C4 Computational gene sets	MSigDB	Genes in the cancer module 81.	TCGA	no
MODULE_83	C4 Computational gene sets	MSigDB	Genes in the cancer module 83.	TCGA	no
MORF_ACP1	C4 Computational gene sets	MSigDB	Neighborhood of ACP1	cohort 1, TCGA	yes
MORF_ACTG1	C4 Computational gene sets	MSigDB	Neighborhood of ACTG1	cohort 1, TCGA	yes
MORF_ANP32B	C4 Computational gene sets	MSigDB	Neighborhood of ANP32B	TCGA	no
MORF_AP2M1	C4 Computational gene sets	MSigDB	Neighborhood of AP2M1	TCGA	no
MORF_AP3D1	C4 Computational gene sets	MSigDB	Neighborhood of AP3D1	TCGA	no
MORF_ATOX1	C4 Computational gene sets	MSigDB	Neighborhood of ATOX1	TCGA	no
MORF_BAG5	C4 Computational gene sets	MSigDB	Neighborhood of BAG5	TCGA	no
MORF_CCNI	C4 Computational gene sets	MSigDB	Neighborhood of CCNI	cohort 1, TCGA	yes
MORF_CSNK2B	C4 Computational gene sets	MSigDB	Neighborhood of CSNK2B	TCGA	no
MORF_CTBP1	C4 Computational gene sets	MSigDB	Neighborhood of CTBP1	TCGA	no
MORF_DAP	C4 Computational gene sets	MSigDB	Neighborhood of DAP	TCGA	no
MORF_DAP3	C4 Computational gene sets	MSigDB	Neighborhood of DAP3	TCGA	no
MORF_DEK	C4 Computational gene sets	MSigDB	Neighborhood of DEK	TCGA	no
MORF_EIF3S6	C4 Computational gene sets	MSigDB	Neighborhood of EIF3S6	TCGA	no
MORF_EIF4A2	C4 Computational gene sets	MSigDB	Neighborhood of EIF4A2	TCGA	no
MORF_ERH	C4 Computational gene sets	MSigDB	Neighborhood of ERH	TCGA	no
MORF_FBL	C4 Computational gene sets	MSigDB	Neighborhood of FBL	TCGA	no
MORF_G22P1	C4 Computational gene sets	MSigDB	Neighborhood of G22P1	TCGA	no
MORF_GPX4	C4 Computational gene sets	MSigDB	Neighborhood of GPX4	TCGA	no
MORF_HDAC1	C4 Computational gene sets	MSigDB	Neighborhood of HDAC1	TCGA	no
MORF_JUND	C4 Computational gene sets	MSigDB	Neighborhood of JUND	TCGA	no
MORF_MAP2K2	C4 Computational gene sets	MSigDB	Neighborhood of MAP2K2	TCGA	no
MORF_NME2	C4 Computational gene sets	MSigDB	Neighborhood of NME2	cohort 1, TCGA	yes
MORF_NPM1	C4 Computational gene sets	MSigDB	Neighborhood of NPM1	cohort 1, TCGA	yes
MORF_PAPSS1	C4 Computational gene sets	MSigDB	Neighborhood of PAPSS1	TCGA	no
MORF_PPP1CA	C4 Computational gene sets	MSigDB	Neighborhood of PPP1CA	TCGA	no
MORF_PPP2R4	C4 Computational gene sets	MSigDB	Neighborhood of PPP2R4	TCGA	no
MORF_PRDX3	C4 Computational gene sets	MSigDB	Neighborhood of PRDX3	TCGA	no
MORF_PRKAR1A	C4 Computational gene sets	MSigDB	Neighborhood of PRKAR1A	TCGA	no
MORF_RAN	C4 Computational gene sets	MSigDB	Neighborhood of RAN	TCGA	no
MORF_SART1	C4 Computational gene sets	MSigDB	Neighborhood of SART1	cohort 1, TCGA	yes
MORF_SKP1A	C4 Computational gene sets	MSigDB	Neighborhood of SKP1A	TCGA	no
MORF_SNRP70	C4 Computational gene sets	MSigDB	Neighborhood of SNRP70	cohort 1	no
MORF_TPT1	C4 Computational gene sets	MSigDB	Neighborhood of TPT1	cohort 1, TCGA	yes

MORF_UBE2I	C4 Computational gene sets	MSigDB	Neighborhood of UBE2I	cohort 1, TCGA	yes
GIMA	Immune modules	Nath et al.	General immune module A (immune system process, defense response, regulation of response to stimulus)	cohort 1	no
GIMB	Immune modules	Nath et al.	General immune module B (immune response-activating signal transduction, positive regulation of immune response, activation of immune response)	cohort 1	no

Tab. S4: Metabolites and metabolite classes included in the targeted metabolomics analysis of cohort 1.

Code	Analyte	Group
Ala	Alanine	Amino Acid
Arg	Arginine	Amino Acid
Asn	Asparagine	Amino Acid
Asp	Aspartate	Amino Acid
Cit	Citrulline	Amino Acid
Gln	Glutamine	Amino Acid
Glu	Glutamate	Amino Acid
Gly	Glycine	Amino Acid
His	Histidine	Amino Acid
Ile	Isoleucine	Amino Acid
Leu	Leucine	Amino Acid
Lys	Lysine	Amino Acid
Met	Methionine	Amino Acid
Orn	Ornithine	Amino Acid
Phe	Phenylalanine	Amino Acid
Pro	Proline	Amino Acid
Ser	Serine	Amino Acid
Thr	Threonine	Amino Acid
Trp	Tryptophan	Amino Acid
Tyr	Tyrosine	Amino Acid
Val	Valine	Amino Acid
Ac-Orn	Acetylornithine	Biogenic Amine
ADMA	Asymmetric dimethylarginine	Biogenic Amine
alpha-AAA	alpha-Aminoadipic acid	Biogenic Amine
c4-OH-Pro	cis-4-Hydroxyproline	Biogenic Amine
Carnosine	Carnosine	Biogenic Amine
Creatinine	Creatinine	Biogenic Amine
DOPA	Dihydroxyphenylalanine	Biogenic Amine
Dopamin	Dopamin	Biogenic Amine
Histamine	Histamine	Biogenic Amine
Kyn	Kynurenine	Biogenic Amine
Met-SO	Methionine-Sulfoxide	Biogenic Amine
Nitro-Tyr	Nitrotyrosine	Biogenic Amine
PEA	Phenylethylamine	Biogenic Amine
Putrescine	Putrescine	Biogenic Amine
Sarcosine	Sarcosine	Biogenic Amine
SDMA	Symmetric dimethylarginine	Biogenic Amine
Serotonin	Serotonin	Biogenic Amine
Spermidine	Spermidine	Biogenic Amine
Spermine	Spermine	Biogenic Amine
t4-OH-Pro	trans-4-Hydroxyproline	Biogenic Amine
Taurine	Taurine	Biogenic Amine
C0	Carnitine (free)	Acylcarnitine
C2	Acetylcarnitine	Acylcarnitine
C3	Propionylcarnitine	Acylcarnitine
C3-DC (C4-OH)	Glutarylcarnitine	Acylcarnitine
C3-OH	Hydroxypropionylcarnitine	Acylcarnitine
C3:1	Propenoylcarnitine	Acylcarnitine
C4	Butyrylcarnitine / Isobutyrylcarnitine	Acylcarnitine
C4:1	Butenoylcarnitine	Acylcarnitine
C5	Isovalerylcarnitine / 2-Methylbutyrylcarnitine / Valerylcarnitine	Acylcarnitine
C5-DC (C6-OH)	Glutarylcarnitine (Hydroxyhexanoylcarnitine [= Hydroxycaproylcarnitine])	Acylcarnitine
C5-M-DC	Methylglutarylcarnitine	Acylcarnitine
C5-OH (C3-DC-M)	Hydroxyisovalerylcarnitine / Hydroxy-2-methylbutyryl / Hydroxyvalerylcarnitine (Methylmalonylcarnitine)	Acylcarnitine

C5:1	Tiglylcarnitine / 3-Methyl-crotonylcarnitine	Acylcarnitine
C5:1-DC	Glutaconylcarnitine / Mesaconylcarnitine	Acylcarnitine
C6 (C4:1-DC)	Hexanoylcarnitine [= Caproylcarnitine] (Fumarylcarnitine)	Acylcarnitine
C6:1	Hexenoylcarnitine	Acylcarnitine
C7-DC	Pimelylcarnitine	Acylcarnitine
C8	Octanoylcarnitine [= Caprylylcarnitine]	Acylcarnitine
C8:1	Octanoylcarnitine	Acylcarnitine
C9	Nonanoylcarnitine [= Pelargonylcarnitine]	Acylcarnitine
C10	Decanoylcarnitine [= Caprylcarnitine]	Acylcarnitine
C10:1	Decenoylcarnitine	Acylcarnitine
C10:2	Decadienoylcarnitine	Acylcarnitine
C12	Dodecanoylcarnitine [= Laurylcarnitine]	Acylcarnitine
C12-DC	Dodecanedioylcarnitine	Acylcarnitine
C12:1	Dodecenoylcarnitine	Acylcarnitine
C14	Tetradecanoylcarnitine [= Myristylcarnitine]	Acylcarnitine
C14:1	Tetradecanoylcarnitine [= Myristoleylcarnitine]	Acylcarnitine
C14:1-OH	Hydroxytetradecenoylcarnitine [= Hydroxymyristoleylcarnitine]	Acylcarnitine
C14:2	Tetradecadienoylcarnitine	Acylcarnitine
C14:2-OH	Hydroxytetradecadienoylcarnitine	Acylcarnitine
C16	Hexadecanoylcarnitine [= Palmitoylcarnitine]	Acylcarnitine
C16-OH	Hydroxyhexadecanolycarnitine [= Hydroxypalmitoylcarnitine]	Acylcarnitine
C16:1	Hexadecenoylcarnitine [= Palmitoleylcarnitine]	Acylcarnitine
C16:1-OH	Hydroxyhexadecenoylcarnitine [= Hydroxypalmitoleylcarnitine]	Acylcarnitine
C16:2	Hexadecadienoylcarnitine	Acylcarnitine
C16:2-OH	Hydroxyhexadecadienoylcarnitine	Acylcarnitine
C18	Octadecanoylcarnitine [= Stearylcarnitine]	Acylcarnitine
C18:1	Octadecenoylcarnitine [= Oleylcarnitine]	Acylcarnitine
C18:1-OH	Hydroxyoctadecenoylcarnitine [= Hydroxyoleylcarnitine]	Acylcarnitine
C18:2	Octadecadienoylcarnitine [= Linoleylcarnitine]	Acylcarnitine
lysoPC a C6:0	Lysophosphatidylcholine with acyl residue C6:0	Lyso-PC
lysoPC a C14:0	Lysophosphatidylcholine with acyl residue C14:0	Lyso-PC
lysoPC a C16:0	Lysophosphatidylcholine with acyl residue C16:0	Lyso-PC
lysoPC a C16:1	Lysophosphatidylcholine with acyl residue C16:1	Lyso-PC
lysoPC a C17:0	Lysophosphatidylcholine with acyl residue C17:0	Lyso-PC
lysoPC a C18:0	Lysophosphatidylcholine with acyl residue C18:0	Lyso-PC
lysoPC a C18:1	Lysophosphatidylcholine with acyl residue C18:1	Lyso-PC
lysoPC a C18:2	Lysophosphatidylcholine with acyl residue C18:2	Lyso-PC
lysoPC a C20:3	Lysophosphatidylcholine with acyl residue C20:3	Lyso-PC
lysoPC a C20:4	Lysophosphatidylcholine with acyl residue C20:4	Lyso-PC
lysoPC a C24:0	Lysophosphatidylcholine with acyl residue C24:0	Lyso-PC
lysoPC a C26:0	Lysophosphatidylcholine with acyl residue C26:0	Lyso-PC
lysoPC a C26:1	Lysophosphatidylcholine with acyl residue C26:1	Lyso-PC
lysoPC a C28:0	Lysophosphatidylcholine with acyl residue C28:0	Lyso-PC
lysoPC a C28:1	Lysophosphatidylcholine with acyl residue C28:1	Lyso-PC
PC aa C24:0	Phosphatidylcholine with diacyl residue sum C24:0	Diacyl-PC
PC aa C26:0	Phosphatidylcholine with diacyl residue sum C26:0	Diacyl-PC
PC aa C28:1	Phosphatidylcholine with diacyl residue sum C28:1	Diacyl-PC
PC aa C30:0	Phosphatidylcholine with diacyl residue sum C30:0	Diacyl-PC
PC aa C30:2	Phosphatidylcholine with diacyl residue sum C30:2	Diacyl-PC
PC aa C32:0	Phosphatidylcholine with diacyl residue sum C32:0	Diacyl-PC
PC aa C32:1	Phosphatidylcholine with diacyl residue sum C32:1	Diacyl-PC
PC aa C32:2	Phosphatidylcholine with diacyl residue sum C32:2	Diacyl-PC
PC aa C32:3	Phosphatidylcholine with diacyl residue sum C32:3	Diacyl-PC
PC aa C34:1	Phosphatidylcholine with diacyl residue sum C34:1	Diacyl-PC
PC aa C34:2	Phosphatidylcholine with diacyl residue sum C34:2	Diacyl-PC
PC aa C34:3	Phosphatidylcholine with diacyl residue sum C34:3	Diacyl-PC
PC aa C34:4	Phosphatidylcholine with diacyl residue sum C34:4	Diacyl-PC
PC aa C36:0	Phosphatidylcholine with diacyl residue sum C36:0	Diacyl-PC

PC aa C36:1	Phosphatidylcholine with diacyl residue sum C36:1	Diacyl-PC
PC aa C36:2	Phosphatidylcholine with diacyl residue sum C36:2	Diacyl-PC
PC aa C36:3	Phosphatidylcholine with diacyl residue sum C36:3	Diacyl-PC
PC aa C36:4	Phosphatidylcholine with diacyl residue sum C36:4	Diacyl-PC
PC aa C36:5	Phosphatidylcholine with diacyl residue sum C36:5	Diacyl-PC
PC aa C36:6	Phosphatidylcholine with diacyl residue sum C36:6	Diacyl-PC
PC aa C38:0	Phosphatidylcholine with diacyl residue sum C38:0	Diacyl-PC
PC aa C38:1	Phosphatidylcholine with diacyl residue sum C38:1	Diacyl-PC
PC aa C38:3	Phosphatidylcholine with diacyl residue sum C38:3	Diacyl-PC
PC aa C38:4	Phosphatidylcholine with diacyl residue sum C38:4	Diacyl-PC
PC aa C38:5	Phosphatidylcholine with diacyl residue sum C38:5	Diacyl-PC
PC aa C38:6	Phosphatidylcholine with diacyl residue sum C38:6	Diacyl-PC
PC aa C40:1	Phosphatidylcholine with diacyl residue sum C40:1	Diacyl-PC
PC aa C40:2	Phosphatidylcholine with diacyl residue sum C40:2	Diacyl-PC
PC aa C40:3	Phosphatidylcholine with diacyl residue sum C40:3	Diacyl-PC
PC aa C40:4	Phosphatidylcholine with diacyl residue sum C40:4	Diacyl-PC
PC aa C40:5	Phosphatidylcholine with diacyl residue sum C40:5	Diacyl-PC
PC aa C40:6	Phosphatidylcholine with diacyl residue sum C40:6	Diacyl-PC
PC aa C42:0	Phosphatidylcholine with diacyl residue sum C42:0	Diacyl-PC
PC aa C42:1	Phosphatidylcholine with diacyl residue sum C42:1	Diacyl-PC
PC aa C42:2	Phosphatidylcholine with diacyl residue sum C42:2	Diacyl-PC
PC aa C42:4	Phosphatidylcholine with diacyl residue sum C42:4	Diacyl-PC
PC aa C42:5	Phosphatidylcholine with diacyl residue sum C42:5	Diacyl-PC
PC aa C42:6	Phosphatidylcholine with diacyl residue sum C42:6	Diacyl-PC
PC ae C30:0	Phosphatidylcholine with acyl-alkyl residue sum C30:0	Acyl/Alkyl-PC
PC ae C30:1	Phosphatidylcholine with acyl-alkyl residue sum C30:1	Acyl/Alkyl-PC
PC ae C30:2	Phosphatidylcholine with acyl-alkyl residue sum C30:2	Acyl/Alkyl-PC
PC ae C32:1	Phosphatidylcholine with acyl-alkyl residue sum C32:1	Acyl/Alkyl-PC
PC ae C32:2	Phosphatidylcholine with acyl-alkyl residue sum C32:2	Acyl/Alkyl-PC
PC ae C34:0	Phosphatidylcholine with acyl-alkyl residue sum C34:0	Acyl/Alkyl-PC
PC ae C34:1	Phosphatidylcholine with acyl-alkyl residue sum C34:1	Acyl/Alkyl-PC
PC ae C34:2	Phosphatidylcholine with acyl-alkyl residue sum C34:2	Acyl/Alkyl-PC
PC ae C34:3	Phosphatidylcholine with acyl-alkyl residue sum C34:3	Acyl/Alkyl-PC
PC ae C36:0	Phosphatidylcholine with acyl-alkyl residue sum C36:0	Acyl/Alkyl-PC
PC ae C36:1	Phosphatidylcholine with acyl-alkyl residue sum C36:1	Acyl/Alkyl-PC
PC ae C36:2	Phosphatidylcholine with acyl-alkyl residue sum C36:2	Acyl/Alkyl-PC
PC ae C36:3	Phosphatidylcholine with acyl-alkyl residue sum C36:3	Acyl/Alkyl-PC
PC ae C36:4	Phosphatidylcholine with acyl-alkyl residue sum C36:4	Acyl/Alkyl-PC
PC ae C36:5	Phosphatidylcholine with acyl-alkyl residue sum C36:5	Acyl/Alkyl-PC
PC ae C38:0	Phosphatidylcholine with acyl-alkyl residue sum C38:0	Acyl/Alkyl-PC
PC ae C38:1	Phosphatidylcholine with acyl-alkyl residue sum C38:1	Acyl/Alkyl-PC
PC ae C38:2	Phosphatidylcholine with acyl-alkyl residue sum C38:2	Acyl/Alkyl-PC
PC ae C38:3	Phosphatidylcholine with acyl-alkyl residue sum C38:3	Acyl/Alkyl-PC
PC ae C38:4	Phosphatidylcholine with acyl-alkyl residue sum C38:4	Acyl/Alkyl-PC
PC ae C38:5	Phosphatidylcholine with acyl-alkyl residue sum C38:5	Acyl/Alkyl-PC
PC ae C38:6	Phosphatidylcholine with acyl-alkyl residue sum C38:6	Acyl/Alkyl-PC
PC ae C40:0	Phosphatidylcholine with acyl-alkyl residue sum C40:0	Acyl/Alkyl-PC
PC ae C40:1	Phosphatidylcholine with acyl-alkyl residue sum C40:1	Acyl/Alkyl-PC
PC ae C40:2	Phosphatidylcholine with acyl-alkyl residue sum C40:2	Acyl/Alkyl-PC
PC ae C40:3	Phosphatidylcholine with acyl-alkyl residue sum C40:3	Acyl/Alkyl-PC
PC ae C40:4	Phosphatidylcholine with acyl-alkyl residue sum C40:4	Acyl/Alkyl-PC
PC ae C40:5	Phosphatidylcholine with acyl-alkyl residue sum C40:5	Acyl/Alkyl-PC
PC ae C40:6	Phosphatidylcholine with acyl-alkyl residue sum C40:6	Acyl/Alkyl-PC
PC ae C42:0	Phosphatidylcholine with acyl-alkyl residue sum C42:0	Acyl/Alkyl-PC
PC ae C42:1	Phosphatidylcholine with acyl-alkyl residue sum C42:1	Acyl/Alkyl-PC
PC ae C42:2	Phosphatidylcholine with acyl-alkyl residue sum C42:2	Acyl/Alkyl-PC
PC ae C42:3	Phosphatidylcholine with acyl-alkyl residue sum C42:3	Acyl/Alkyl-PC
PC ae C42:4	Phosphatidylcholine with acyl-alkyl residue sum C42:4	Acyl/Alkyl-PC
PC ae C42:5	Phosphatidylcholine with acyl-alkyl residue sum C42:5	Acyl/Alkyl-PC
PC ae C44:3	Phosphatidylcholine with acyl-alkyl residue sum C44:3	Acyl/Alkyl-PC
PC ae C44:4	Phosphatidylcholine with acyl-alkyl residue sum C44:4	Acyl/Alkyl-PC

PC ae C44:5	Phosphatidylcholine with acyl-alkyl residue sum C44:5	Acyl/Alkyl-PC
PC ae C44:6	Phosphatidylcholine with acyl-alkyl residue sum C44:6	Acyl/Alkyl-PC
SM (OH) C14:1	Hydroxysphingomyelin with acyl residue sum C14:1	OH-Sphingomyelin
SM (OH) C16:1	Hydroxysphingomyelin with acyl residue sum C16:1	OH-Sphingomyelin
SM (OH) C22:1	Hydroxysphingomyelin with acyl residue sum C22:1	OH-Sphingomyelin
SM (OH) C22:2	Hydroxysphingomyelin with acyl residue sum C22:2	OH-Sphingomyelin
SM (OH) C24:1	Hydroxysphingomyelin with acyl residue sum C24:1	OH-Sphingomyelin
SM C16:0	Sphingomyelin with acyl residue sum C16:0	Sphingomyelin
SM C16:1	Sphingomyelin with acyl residue sum C16:1	Sphingomyelin
SM C18:0	Sphingomyelin with acyl residue sum C18:0	Sphingomyelin
SM C18:1	Sphingomyelin with acyl residue sum C18:1	Sphingomyelin
SM C20:2	Sphingomyelin with acyl residue sum C20:2	Sphingomyelin
SM C22:3	Sphingomyelin with acyl residue sum C22:3	Sphingomyelin
SM C24:0	Sphingomyelin with acyl residue sum C24:0	Sphingomyelin
SM C24:1	Sphingomyelin with acyl residue sum C24:1	Sphingomyelin
SM C26:0	Sphingomyelin with acyl residue sum C26:0	Sphingomyelin
SM C26:1	Sphingomyelin with acyl residue sum C26:1	Sphingomyelin
H1	Glucose and other Hexoses	Energy Metabolism
Glc	Glucose	Energy Metabolism
Ribose	Ribose	Energy Metabolism
2-HG	2-hydroxyglutaric acid	Energy Metabolism
alpha-KGA	alpha-ketoglutaric acid (=2-Oxoglutaric acid)	Energy Metabolism
Fum	Fumaric acid	Energy Metabolism
Mal	Malic acid	Energy Metabolism
Suc	Succinic acid	Energy Metabolism
2-HBA	2-hydroxybutyric acid	Energy Metabolism
3-HBA	3-2ydroxybutyric acid	Energy Metabolism
Lac	Lactic acid	Energy Metabolism
Pyr + OAA	Sum of Pyruvate + Oxalacetic acid	Energy Metabolism
CitA	Citric acid	Energy Metabolism
Isocit	Isocitric acid	Energy Metabolism

Tab. S5: Immuno-oncological marker proteins and associated biological processes.

Marker	Process 1	Process 2	Process 3	Process 4
ADA	Metabolism and Autophagy			
ADGRG1	Vascular and Tissue Remodeling			
ANG-1	Vascular and Tissue Remodeling			
ANGPT2	Vascular and Tissue Remodeling			
ARG1	Metabolism and Autophagy			
CAIX	Metabolism and Autophagy			
CASP-8	Apoptosis and Cell Killing			
CCL17	Chemotaxis	Suppression of Tumor Immunity		
CCL19	Chemotaxis	Suppression of Tumor Immunity		
CCL20	Chemotaxis	Suppression of Tumor Immunity		
CCL23	Chemotaxis	Vascular and Tissue Remodeling		
CCL3	Chemotaxis			
CCL4	Chemotaxis			
CD244	Promotion of Tumor Immunity			
CD27	Promotion of Tumor Immunity			
CD28	Promotion of Tumor Immunity			
CD4	Promotion of Tumor Immunity	Suppression of Tumor Immunity		
CD40	Promotion of Tumor Immunity			
CD40-L	Apoptosis and Cell Killing	Promotion of Tumor Immunity		
CD5	Promotion of Tumor Immunity	Suppression of Tumor Immunity		
CD70	Promotion of Tumor Immunity			
CD83	Promotion of Tumor Immunity			
CD8A	Promotion of Tumor Immunity			
CRTAM	Promotion of Tumor Immunity			
CSF-1	Suppression of Tumor Immunity			
CX3CL1	Promotion of Tumor Immunity	Chemotaxis		
CXCL1	Chemotaxis	Suppression of Tumor Immunity	Vascular and Tissue Remodeling	
CXCL10	Chemotaxis	Promotion of Tumor Immunity	Vascular and Tissue Remodeling	
CXCL11	Chemotaxis	Promotion of Tumor Immunity	Suppression of Tumor Immunity	Vascular and Tissue Remodeling
CXCL12	Suppression of Tumor Immunity	Vascular and Tissue Remodeling	Chemotaxis	
CXCL13	Promotion of	Suppression of	Chemotaxis	

	Tumor Immunity	Tumor Immunity		
CXCL5	Chemotaxis	Suppression of Tumor Immunity	Vascular and Tissue Remodeling	
CXCL9	Chemotaxis	Promotion of Tumor Immunity	Vascular and Tissue Remodeling	
DCN	Vascular and Tissue Remodeling			
EGF	Vascular and Tissue Remodeling			
FASLG	Apoptosis and Cell Killing			
FGF2	Vascular and Tissue Remodeling			
Gal-1	Suppression of Tumor Immunity	Vascular and Tissue Remodeling		
Gal-9	Apoptosis and Cell Killing	Suppression of Tumor Immunity	Vascular and Tissue Remodeling	
GZMA	Apoptosis and Cell Killing			
GZMB	Apoptosis and Cell Killing			
GZMH	Apoptosis and Cell Killing			
HGF	Vascular and Tissue Remodeling			
HO-1	Metabolism and Autophagy			
ICOSLG	Promotion of Tumor Immunity			
IFN-beta	Promotion of Tumor Immunity			
IFN-gamma	Promotion of Tumor Immunity			
IL-1 alpha	Promotion of Tumor Immunity	Suppression of Tumor Immunity	Vascular and Tissue Remodeling	
IL10	Suppression of Tumor Immunity			
IL12	Promotion of Tumor Immunity			
IL12RB1	Promotion of Tumor Immunity			
IL13	Suppression of Tumor Immunity			
IL18	Promotion of Tumor Immunity	Suppression of Tumor Immunity		
IL2	Promotion of Tumor Immunity			
IL-21	Promotion of Tumor Immunity			
IL33	Suppression of Tumor Immunity			
IL-35	Suppression of Tumor Immunity			
IL4	Suppression of Tumor Immunity			
IL5	Suppression of Tumor Immunity			
IL6	Promotion of Tumor Immunity	Suppression of Tumor Immunity		
IL7	Promotion of Tumor Immunity			
IL8	Suppression of	Vascular and Tissue	Chemotaxis	

	Tumor Immunity	Remodeling		
KLRD1	Promotion of Tumor Immunity			
LAMP3	Suppression of Tumor Immunity			
LAP TGF-beta-1	Suppression of Tumor Immunity			
MCP-1	Chemotaxis	Vascular and Tissue Remodeling		
MCP-2	Chemotaxis			
MCP-3	Chemotaxis			
MCP-4	Chemotaxis	Suppression of Tumor Immunity	Vascular and Tissue Remodeling	
MIC-A/B	Suppression of Tumor Immunity			
MMP12	Suppression of Tumor Immunity	Vascular and Tissue Remodeling		
MMP7	Apoptosis and Cell Killing	Suppression of Tumor Immunity		
NCR1	Promotion of Tumor Immunity			
NOS3	Vascular and Tissue Remodeling			
PDCD1	Suppression of Tumor Immunity			
PDGF subunit B	Vascular and Tissue Remodeling			
PD-L1	Suppression of Tumor Immunity			
PD-L2	Suppression of Tumor Immunity			
PGF	Vascular and Tissue Remodeling			
PTN	Vascular and Tissue Remodeling			
TIE2	Vascular and Tissue Remodeling			
TNF	Promotion of Tumor Immunity	Suppression of Tumor Immunity	Vascular and Tissue Remodeling	
TNFRSF12A	Apoptosis and Cell Killing	Vascular and Tissue Remodeling		
TNFRSF21	Apoptosis and Cell Killing			
TNFRSF4	Promotion of Tumor Immunity			
TNFRSF9	Promotion of Tumor Immunity			
TNFSF14	Promotion of Tumor Immunity			
TRAIL	Apoptosis and Cell Killing			
TWEAK	Apoptosis and Cell Killing	Vascular and Tissue Remodeling		
VEGFA	Vascular and Tissue Remodeling			
VEGFC	Vascular and Tissue Remodeling			
VEGFR-2	Vascular and Tissue Remodeling			

Tab. S6: Differentially methylated CpG sites in candidate gene regions (TCGA KIRC cohort). Shown are those positions with significantly decreased methylation in tumor tissues (difference in β-values (tumor-normal) ≤ -0.1; p-value < 0.05), which were negatively correlated with gene expression (Rs < -0.3; p-value < 0.05).

Gene Symbol	CpG site	Difference in β-values (tumor-normal) unpaired	p-value unpaired	Difference in β-values (tumor-normal) paired	p-value paired	R (Spearman)	p-value (Spearman)
ADM	cg03246478	-0.28	1.13E-59	-0.28	3.73E-25	-0.34	7.87E-09
ALDOA	cg00583733	-0.14	1.38E-45	-0.14	2.19E-22	-0.44	1.64E-14
	cg04416734	-0.16	1.79E-45	-0.16	5.33E-22	-0.47	< 1.00E-16
ALDOC	cg15835620	-0.25	3.15E-33	-0.19	1.13E-19	-0.48	< 1.00E-16
	cg14668747	-0.20	2.31E-31	-0.13	1.36E-12	-0.47	4.44E-16
	cg27183188	-0.26	1.87E-30	-0.17	4.16E-14	-0.52	< 1.00E-16
ANXA2	cg13313836	-0.22	2.30E-49	-0.25	1.21E-24	-0.49	< 1.00E-16
	cg06738887	-0.38	6.52E-58	-0.37	4.90E-25	-0.46	1.33E-15
ANXA4	cg12518775	-0.15	9.42E-41	-0.15	3.55E-21	-0.31	1.96E-07
	cg25099065	-0.12	2.19E-20	-0.12	9.25E-14	-0.31	1.14E-07
ANXA5	cg19130824	-0.16	1.47E-44	-0.19	1.34E-22	-0.34	4.99E-09
	cg10728351	-0.30	1.74E-43	-0.30	2.91E-23	-0.42	4.38E-13
B2M	cg18696027	-0.19	4.83E-39	-0.22	1.07E-21	-0.38	1.46E-10
BNIP3	cg02365648	-0.23	1.44E-32	-0.21	6.62E-22	-0.44	2.98E-14
C3	cg14279361	-0.21	2.55E-19	-0.16	2.11E-15	-0.36	7.24E-10
CCND1	cg09637363	-0.14	3.19E-45	-0.13	4.35E-23	-0.34	1.04E-08
	cg13608094	-0.13	3.63E-37	-0.14	5.36E-21	-0.34	1.07E-08
	cg12266049	-0.14	3.90E-28	-0.16	8.82E-20	-0.38	5.83E-11
	cg19047670	-0.18	6.71E-44	-0.18	6.12E-23	-0.31	2.45E-07
EDIL3	cg19722082	-0.25	1.41E-35	-0.25	6.12E-22	-0.33	3.39E-08
EGLN3	cg06816517	-0.23	1.71E-60	-0.24	3.57E-25	-0.43	1.17E-13
	cg24170040	-0.56	7.06E-63	-0.54	3.28E-25	-0.42	4.13E-13
	cg02000275	-0.28	4.94E-52	-0.28	6.85E-25	-0.41	1.20E-12
	cg13728308	-0.31	7.75E-58	-0.29	5.33E-25	-0.49	< 1.00E-16
GAPDH	cg26826729	-0.36	5.76E-56	-0.32	9.00E-25	-0.42	3.31E-13
GLRX	cg02354658	-0.24	3.43E-36	-0.25	4.83E-22	-0.40	5.18E-12
	cg22689909	-0.32	2.08E-45	-0.29	9.40E-24	-0.35	4.63E-09
	cg16677191	-0.39	3.17E-56	-0.38	4.32E-25	-0.33	2.55E-08
HLA-B	cg13031097	-0.34	5.15E-58	-0.32	4.05E-25	-0.32	6.55E-08
	cg23923934	-0.20	7.84E-53	-0.25	1.34E-24	-0.42	7.62E-13
	cg27529346	-0.15	2.95E-45	-0.19	4.61E-21	-0.40	8.34E-11
	cg03154077	-0.16	7.61E-54	-0.16	3.97E-25	-0.37	1.62E-10
	cg11187245	-0.28	3.03E-54	-0.29	8.63E-25	-0.41	9.08E-13
HLA-C	cg18511546	-0.21	7.46E-56	-0.23	6.57E-25	-0.39	2.53E-11
	cg26392102	-0.14	5.17E-49	-0.15	2.25E-24	-0.38	6.43E-11
	cg17096289	-0.13	1.97E-13	-0.18	8.26E-20	-0.40	9.08E-12
	cg14931776	-0.16	1.23E-18	-0.13	1.01E-18	-0.33	2.78E-08
HLA-DMA	cg03531211	-0.13	1.88E-41	-0.14	1.77E-23	-0.52	< 1.00E-16
	cg24421410	-0.22	1.99E-38	-0.23	6.50E-23	-0.37	1.75E-10
	cg02806715	-0.23	6.50E-33	-0.28	3.01E-22	-0.41	1.20E-12
	cg08735211	-0.10	3.51E-21	-0.13	2.59E-19	-0.52	< 1.00E-16
	cg24129356	-0.29	8.13E-44	-0.31	1.19E-23	-0.45	8.88E-15
	cg03520342	-0.26	7.44E-50	-0.24	1.79E-23	-0.41	9.76E-13
	cg07151443	-0.28	7.26E-54	-0.25	1.37E-24	-0.37	1.86E-10
HLA-E	cg21366673	-0.12	4.07E-38	-0.12	1.19E-20	-0.38	7.19E-11
	cg23235965	-0.29	3.20E-59	-0.31	3.81E-25	-0.32	9.73E-08
HTRA1	cg00701951	-0.43	7.11E-60	-0.42	3.57E-25	-0.37	2.06E-10
IFI30	cg04610450	-0.17	3.46E-46	-0.15	1.60E-24	-0.31	2.25E-07
ITGB2	cg07274406	-0.16	1.03E-36	-0.18	1.78E-22	-0.51	< 1.00E-16
	cg06654697	-0.21	1.56E-37	-0.20	8.81E-21	-0.57	< 1.00E-16
	cg18663307	-0.11	3.19E-40	-0.12	7.44E-22	-0.60	< 1.00E-16

	cg14112356	-0.12	1.08E-13	-0.11	1.54E-11	-0.40	1.22E-11
	cg21006727	-0.10	1.10E-21	-0.17	2.44E-21	-0.34	7.45E-09
KCNMA1	cg16055185	-0.17	3.14E-26	-0.15	1.85E-17	-0.41	3.34E-12
	cg26353166	-0.27	7.74E-50	-0.24	1.07E-24	-0.42	6.11E-13
	cg02172819	-0.14	2.31E-45	-0.12	1.27E-19	-0.35	2.58E-09
	cg18738985	-0.20	4.94E-24	-0.15	6.12E-16	-0.31	1.16E-07
	cg03299991	-0.21	1.19E-46	-0.18	3.31E-23	-0.30	3.56E-07
KLF7	cg02577773	-0.18	4.52E-41	-0.18	2.33E-22	-0.34	1.21E-08
KRT8	cg17781464	-0.11	1.64E-20	-0.11	1.30E-12	-0.40	5.26E-12
	cg21173150	-0.25	3.01E-57	-0.26	5.33E-25	-0.41	1.05E-12
LOXL2	cg24531955	-0.49	3.28E-61	-0.45	3.43E-25	-0.46	2.22E-15
MET	cg24258705	-0.13	2.00E-37	-0.19	9.90E-23	-0.38	5.42E-11
NDRG1	cg04227278	-0.14	9.28E-37	-0.14	6.99E-20	-0.36	1.24E-09
NNMT	cg14520913	-0.39	6.77E-61	-0.37	3.73E-25	-0.37	2.33E-10
P4HA2	cg09830083	-0.28	3.51E-52	-0.27	9.19E-25	-0.39	2.29E-11
PEA15	cg26835683	-0.14	2.68E-17	-0.13	1.89E-12	-0.32	6.44E-08
PFKP	cg24882709	-0.38	6.41E-59	-0.37	3.43E-25	-0.42	2.15E-13
	cg08194405	-0.18	5.74E-57	-0.21	3.77E-25	-0.36	7.48E-10
	cg26693553	-0.26	6.13E-24	-0.18	9.09E-15	-0.32	1.03E-07
PGF	cg15890469	-0.16	1.31E-49	-0.17	3.20E-24	-0.43	1.65E-13
PKM2	cg22234930	-0.26	5.22E-55	-0.26	7.45E-25	-0.32	7.00E-08
PLIN2	cg03885527	-0.28	2.88E-48	-0.29	1.21E-24	-0.52	< 1.00E-16
	cg14334810	-0.20	3.93E-42	-0.22	1.91E-22	-0.39	1.76E-11
PLOD2	cg24291309	-0.19	2.48E-50	-0.21	2.07E-24	-0.35	2.70E-09
RBPMS	cg02723975	-0.16	2.48E-17	-0.15	5.98E-11	-0.35	1.94E-09
	cg24296569	-0.17	9.99E-24	-0.16	2.23E-14	-0.35	3.20E-09
	cg14895559	-0.20	8.87E-22	-0.17	7.17E-13	-0.34	7.65E-09
	cg03186436	-0.17	4.45E-33	-0.18	9.95E-20	-0.39	4.04E-11
	cg00997969	-0.30	1.77E-60	-0.30	3.73E-25	-0.39	1.88E-11
SCARB1	cg22775642	-0.28	8.65E-59	-0.29	4.70E-25	-0.43	1.69E-13
	cg13075279	-0.22	1.13E-49	-0.22	3.38E-23	-0.35	3.73E-09
	cg14846380	-0.24	7.38E-51	-0.21	8.23E-24	-0.35	1.67E-09
	cg23460943	-0.37	8.46E-51	-0.32	1.79E-24	-0.42	4.25E-13
	cg15282973	-0.42	1.32E-58	-0.37	6.44E-25	-0.47	< 1.00E-16
SCD	cg06400428	-0.17	2.03E-42	-0.17	2.95E-19	-0.30	4.15E-07
	cg03440556	-0.33	3.48E-47	-0.30	8.96E-23	-0.36	8.34E-10
	cg02237755	-0.12	5.63E-27	-0.12	8.52E-17	-0.42	4.08E-13
	cg16744911	-0.21	2.33E-48	-0.24	4.09E-24	-0.50	< 1.00E-16
SHMT2	cg11851129	-0.10	3.92E-48	-0.15	2.10E-23	-0.42	2.51E-13
	cg19535267	-0.22	6.74E-49	-0.23	1.90E-23	-0.52	< 1.00E-16
	cg12639933	-0.27	2.29E-59	-0.26	4.05E-25	-0.45	3.33E-15
	cg08163918	-0.40	2.89E-62	-0.38	3.28E-25	-0.56	< 1.00E-16
SLAMF7	cg04244970	-0.15	1.12E-51	-0.19	2.82E-24	-0.47	2.22E-16
	cg07837085	-0.21	8.48E-55	-0.25	4.41E-25	-0.31	2.26E-07
SPARC	cg10505630	-0.13	2.50E-52	-0.14	2.25E-24	-0.32	4.46E-08
TRIB3	cg15799353	-0.22	1.40E-47	-0.24	5.13E-24	-0.59	< 1.00E-16
	cg24966406	-0.16	2.66E-34	-0.15	1.80E-18	-0.54	< 1.00E-16
VCAM1	cg24593324	-0.39	4.77E-58	-0.35	4.50E-25	-0.31	1.25E-07
VEGFA	cg13320558	-0.23	3.25E-49	-0.22	2.10E-23	-0.46	1.33E-15
	cg01298514	-0.35	2.84E-60	-0.33	4.90E-25	-0.43	1.25E-13
	cg18022921	-0.28	4.13E-58	-0.27	1.19E-24	-0.45	7.99E-15
	cg00539360	-0.32	4.69E-60	-0.32	4.23E-25	-0.51	< 1.00E-16
	cg25343661	-0.34	2.31E-58	-0.35	3.50E-25	-0.41	1.37E-12
	cg25373579	-0.26	1.30E-58	-0.24	6.44E-25	-0.45	2.89E-15
	cg01353538	-0.17	1.23E-57	-0.16	4.70E-25	-0.32	7.66E-08
VIM	cg10790685	-0.36	2.47E-46	-0.32	2.60E-24	-0.30	3.51E-07
	cg14260889	-0.31	1.35E-61	-0.33	3.50E-25	-0.41	1.47E-12

Tab. S7: Somatic mutations of candidate genes in the TCGA patient cohort.

Hugo Symbol	NCBI Build	Chromo-some	Start Position	End Position	Strand	Variant Classific.	Variant Type	Reference Allele	Tumor Seq Allele1	Tumor Seq Allele2	Tumor Sample Barcode
ABCA1	37	9	107651383	107651383	+	Missense Mutation	SNV	A	A	T	TCGA-B0-4823-01A-02D-1421-08
ABCA1	37	9	107618219	107618219	+	Missense Mutation	SNV	C	C	G	TCGA-B0-5088-01A-01D-1462-08
ABCA1	37	9	107558370	107558370	+	Missense Mutation	SNV	G	G	C	TCGA-B0-5109-01A-02D-1421-08
ABCA1	37	9	107589428	107589428	+	Missense Mutation	SNV	C	C	T	TCGA-BP-4803-01
ABCA1	37	9	107558674	107558674	+	Missense Mutation	SNV	A	A	C	TCGA-BP-4992-01
ABCA1	37	9	107564349	107564349	+	Missense Mutation	SNV	G	G	T	TCGA-BP-5008-01
ABCA1	37	9	107573139	107573139	+	Missense Mutation	SNV	T	T	C	TCGA-CJ-4873-01A-01D-1373-10
ABCA1	37	9	107573110	107573110	+	Missense Mutation	SNV	C	C	A	TCGA-CZ-5460-01A-01D-1501-10
ABI3BP	37	3	100595388	100595388	+	Missense Mutation	SNV	C	C	G	TCGA-B0-5080-01A-01D-1501-10
ACLY	37	17	40049295	40049295	+	Missense Mutation	SNV	T	T	G	TCGA-A3-3380-01A-01D-0966-08
ACLY	37	17	40028034	40028034	+	Missense Mutation	SNV	C	C	T	TCGA-A3-3383-01A-01D-0966-08
ACLY	37	17	40039402	40039402	+	Silent	SNV	C	C	T	TCGA-B0-4833-01
ACLY	37	17	40054059	40054059	+	Nonsense Mutation	SNV	C	C	A	TCGA-B0-5702-01A-11D-1534-10
ACLY	37	17	40061042	40061042	+	Silent	SNV	G	G	T	TCGA-BP-4968-01A-01D-1462-08
AGRN	37	1	985094	985094	+	Frame Shift Del	DEL	C	C	-	TCGA-B0-5110-01
AGRN	37	1	981443	981443	+	Missense Mutation	SNV	C	C	G	TCGA-BP-4162-01
AGRN	37	1	970662	970662	+	Missense Mutation	SNV	C	C	A	TCGA-BP-4982-01A-01D-1462-08
AGRN	37	1	979247	979251	+	Frame Shift Del	DEL	GCAGG	GCAGG	-	TCGA-CJ-4902-01A-01D-1429-08

AGRN	37	1	985343	985343	+	Missense Mutation	SNV	C	C	A	TCGA-CW-6093-01A-11D-1669-08
ALDOA	37	16	30080933	30080933	+	Missense Mutation	SNV	T	T	G	TCGA-B0-4714-01
ALDOA	37	16	30078964	30078964	+	Frame Shift Del	DEL	C	C	-	TCGA-BP-5004-01A-01D-1462-08
ANGPTL4	37	19	8429515	8429515	+	Missense Mutation	SNV	A	A	T	TCGA-A3-3378-01A-01D-0966-08
ANGPTL4	37	19	8438683	8438683	+	Missense Mutation	SNV	C	C	G	TCGA-B0-4691-01A-01D-1361-10
ANXA2	37	15	60641324	60641324	+	Silent	SNV	T	T	G	TCGA-B8-5549-01A-01D-1534-10
ANXA4	37	2	70045736	70045736	+	Missense Mutation	SNV	G	G	A	TCGA-A3-3346-01A-01D-0966-08
ANXA4	37	2	70008703	70008703	+	Silent	SNV	C	C	A	TCGA-CJ-4635-01A-02D-1373-10
ARCN1	37	11	118454621	118454621	+	Missense Mutation	SNV	G	G	T	TCGA-BP-4787-01A-01D-1373-10
ARCN1	37	11	118454666	118454666	+	Missense Mutation	SNV	C	C	T	TCGA-BP-5201-01A-01D-1429-08
ARCN1	37	11	118473069	118473069	+	RNA	DEL	G	G	-	TCGA-CJ-5678-01A-11D-1534-10
B2M	37	15	45007705	45007705	+	Missense Mutation	SNV	A	A	C	TCGA-B8-4620-01
B2M	37	15	45007701	45007701	+	Frame Shift Del	DEL	T	T	-	TCGA-B8-4620-01
BGN	37	X	152773750	152773750	+	Silent	SNV	C	C	T	TCGA-DV-5569-01A-01D-1534-10
C3	37	19	6686809	6686809	+	Missense Mutation	SNV	C	C	G	TCGA-B0-5107-01
C3	37	19	6694615	6694615	+	Missense Mutation	SNV	G	G	C	TCGA-B0-5108-01A-01D-1421-08
C3	37	19	6714022	6714022	+	Nonsense Mutation	SNV	C	C	A	TCGA-B0-5696-01A-11D-1534-10
C3	37	19	6681963	6681963	+	Missense Mutation	SNV	A	A	G	TCGA-BP-4998-01A-01D-1462-08
C3	37	19	6702221	6702221	+	Missense Mutation	SNV	A	A	T	TCGA-CJ-4887-01A-01D-1373-10
C3	37	19	6712358	6712358	+	Silent	SNV	G	G	A	TCGA-CZ-5452-01A-01D-1501-10

C3	37	19	6697533	6697533	+	Missense Mutation	SNV	C	C	T	TCGA-CZ-5459-01A-01D-1501-10
CANX	37	5	179149818	179149818	+	Missense Mutation	SNV	C	C	A	TCGA-CJ-4920-01A-01D-1429-08
CEBPA	37	19	33792421	33792421	+	Silent	SNV	G	G	A	TCGA-B0-4714-01
CEBPA	37	19	33792387	33792387	+	Nonsense Mutation	SNV	G	G	A	TCGA-BP-4173-01A-02D-1366-10
CEBPA	37	19	33792412	33792412	+	Silent	SNV	G	G	T	TCGA-CJ-4889-01A-01D-1373-10
COL1A1	37	17	48272628	48272628	+	Missense Mutation	SNV	C	C	T	TCGA-B0-5088-01A-01D-1462-08
COL1A1	37	17	48266574	48266574	+	Silent	SNV	A	A	G	TCGA-B0-5692-01A-11D-1534-10
COL1A1	37	17	48267076	48267076	+	Silent	SNV	A	A	T	TCGA-CJ-4905-01A-02D-1429-08
COL1A2	37	7	94038888	94038888	+	Missense Mutation	SNV	G	G	T	TCGA-B2-3924-01
COL1A2	37	7	94040381	94040381	+	Missense Mutation	SNV	G	G	C	TCGA-BP-4174-01A-02D-1366-10
COL1A2	37	7	94059617	94059617	+	Missense Mutation	SNV	G	G	A	TCGA-BP-4960-01A-01D-1462-08
COL1A2	37	7	94050324	94050324	+	Missense Mutation	SNV	C	C	A	TCGA-CJ-4912-01A-01D-1429-08
COL1A2	37	7	94051214	94051214	+	Missense Mutation	SNV	A	A	G	TCGA-CZ-5456-01A-01D-1501-10
COL4A2	37	13	111156500	111156500	+	Missense Mutation	SNV	C	C	T	TCGA-B0-4691-01A-01D-1361-10
COL4A2	37	13	111102707	111102707	+	Silent	SNV	C	C	A	TCGA-B0-5085-01A-01D-1462-08
COL4A2	37	13	111125466	111125466	+	Silent	SNV	T	T	A	TCGA-CW-5585-01A-01D-1534-10
COL4A2	37	13	111144511	111144511	+	Silent	SNV	T	T	C	TCGA-CZ-5456-01A-01D-1501-10
COL6A2	37	21	47545941	47545941	+	Missense Mutation	SNV	C	C	T	TCGA-B0-5701-01A-11D-1534-10
COL6A2	37	21	47533975	47533975	+	Nonsense Mutation	SNV	C	C	A	TCGA-B4-5377-01A-01D-1501-10
COL6A2	37	21	47542435	47542435	+	Missense Mutation	SNV	G	G	A	TCGA-BP-4998-01A-01D-1462-08

COL6A2	37	21	47531392	47531392	+	Missense Mutation	SNV	T	T	C	TCGA-BP-5000-01A-01D-1462-08
COL6A2	37	21	47532722	47532722	+	Missense Mutation	SNV	A	A	T	TCGA-CJ-4881-01A-01D-1373-10
EDIL3	37	5	83402558	83402558	+	Missense Mutation	SNV	T	T	C	TCGA-B0-4814-01A-01D-1361-10
EDIL3	37	5	83356228	83356228	+	Missense Mutation	SNV	G	G	A	TCGA-BP-4989-01A-01D-1462-08
EDIL3	37	5	83402565	83402565	+	Nonsense Mutation	SNV	C	C	A	TCGA-CZ-5461-01A-01D-1501-10
EEF1A1	37	6	74227570	74227570	+	Missense Mutation	SNV	A	A	T	TCGA-A3-3387-01A-01D-1534-10
EEF1A1	37	6	74228564	74228564	+	Nonsense Mutation	SNV	C	C	T	TCGA-BP-4759-01
EEF1A1	37	6	74228230	74228230	+	Silent	SNV	G	G	T	TCGA-CJ-5686-01A-11D-1669-08
FCGR1A	37	1	149763191	149763191	+	RNA	SNV	G	G	A	TCGA-CJ-4643-01A-02D-1386-10
FCGR1A	37	1	149763191	149763191	+	RNA	SNV	G	G	A	TCGA-CJ-5675-01
FCGR1A	37	1	149763191	149763191	+	RNA	SNV	G	G	A	TCGA-CW-5589-01A-01D-1534-10
FN1	37	2	216298131	216298131	+	Missense Mutation	SNV	T	T	G	TCGA-A3-3308-01A-01D-0966-08
FN1	37	2	216274775	216274775	+	Missense Mutation	SNV	G	G	C	TCGA-A3-3358-01A-01D-1534-10
FN1	37	2	216236992	216236992	+	Silent	SNV	C	C	A	TCGA-A3-3365-01
FN1	37	2	216295547	216295547	+	Silent	SNV	C	C	T	TCGA-BP-4787-01A-01D-1373-10
FN1	37	2	216262451	216262451	+	Missense Mutation	SNV	T	T	C	TCGA-BP-4989-01A-01D-1462-08
FN1	37	2	216245654	216245654	+	Missense Mutation	SNV	C	C	G	TCGA-BP-5009-01A-01D-1462-08
FSCN1	37	7	5632985	5632985	+	Missense Mutation	SNV	C	C	T	TCGA-BP-4967-01A-01D-1462-08
FSCN1	37	7	5632742	5632742	+	Missense Mutation	SNV	G	G	C	TCGA-BP-5185-01A-01D-1429-08
FSCN1	37	7	5643128	5643128	+	Missense Mutation	SNV	A	A	T	TCGA-CW-5588-01A-01D-1534-10

FUS	37	16	31193930	31193930	+	Silent	SNV	G	G	A	TCGA-B2-5641-01A-01D-1534-10
FUS	37	16	31193931	31193931	+	Missense Mutation	SNV	G	G	C	TCGA-B2-5641-01A-01D-1534-10
FUS	37	16	31202109	31202109	+	Missense Mutation	SNV	T	T	G	TCGA-B8-5553-01A-01D-1534-10
FUS	37	16	31202110	31202110	+	Missense Mutation	SNV	G	G	T	TCGA-B8-5553-01A-01D-1534-10
FUS	37	16	31202112	31202112	+	Nonsense Mutation	SNV	A	A	T	TCGA-B8-5553-01A-01D-1534-10
FUS	37	16	31202116	31202116	+	Frame Shift Del	DEL	C	C	-	TCGA-B8-5553-01A-01D-1534-10
FUS	37	16	31201624	31201624	+	Silent	SNV	T	T	C	TCGA-EU-5905-01A-11D-1669-08
GAA	37	17	78090863	78090863	+	Missense Mutation	SNV	A	A	T	TCGA-BP-5187-01
GAA	37	17	78090845	78090845	+	Silent	SNV	C	C	T	TCGA-CJ-6027-01A-11D-1669-08
GAL3ST1	37	22	30952062	30952062	+	Frame Shift Del	DEL	C	C	-	TCGA-BP-5185-01A-01D-1429-08
GAL3ST1	37	22	30951966	30951966	+	Missense Mutation	SNV	C	C	A	TCGA-CW-6087-01A-11D-1669-08
HLA-A	37	6	29910607	29910607	+	Silent	SNV	G	G	C	TCGA-CJ-4640-01A-02D-1386-10
HLA-A	37	6	29912029	29912029	+	Frame Shift Del	DEL	G	G	-	TCGA-CW-6087-01A-11D-1669-08
HLA-C	37	6	31237817	31237817	+	Missense Mutation	SNV	C	C	A	TCGA-BP-4759-01
HLA-C	37	6	31237140	31237140	+	Missense Mutation	SNV	A	A	T	TCGA-CZ-5469-01A-01D-1501-10
HLA-DOA	37	6	32974563	32974563	+	Missense Mutation	SNV	C	C	A	TCGA-BP-4801-01A-02D-1421-08
HLA-DRA	37	6	32410977	32410978	+	Frame Shift Del	DEL	CT	CT	-	TCGA-BP-5192-01A-01D-1429-08
HLA-DRB1	37	6	32551957	32551957	+	Missense Mutation	SNV	G	G	C	TCGA-B0-5701-01A-11D-1534-10
HLA-DRB5	37	6	32497905	32497905	+	Nonsense Mutation	SNV	G	G	A	TCGA-BP-4176-01A-02D-1366-10
HLA-DRB5	37	6	32497905	32497905	+	Nonsense Mutation	SNV	G	G	A	TCGA-BP-4759-01

HLA-E	37	6	30457316	30457316	+	Missense Mutation	SNV	A	A	G	TCGA-B0-4842-01A-02D-1421-08
HLA-E	37	6	30457321	30457321	+	Missense Mutation	SNV	A	A	G	TCGA-BP-5187-01
HLA-E	37	6	30459116	30459116	+	Silent	SNV	T	T	A	TCGA-CJ-5682-01A-11D-1534-10
HMOX1	37	22	35783139	35783139	+	Missense Mutation	SNV	G	G	T	TCGA-BP-5173-01A-01D-1429-08
HMOX1	37	22	35785885	35785885	+	Missense Mutation	SNV	C	C	A	TCGA-CJ-6028-01A-11D-1669-08
IFI27	37	14	94581236	94581236	+	Missense Mutation	SNV	T	T	G	TCGA-BP-4761-01A-01D-1366-10
IFI30	37	19	18286174	18286174	+	Silent	SNV	G	G	A	TCGA-B0-4710-01
IFI30	37	19	18286140	18286140	+	Missense Mutation	SNV	G	G	C	TCGA-BP-4163-01
IGFBP3	37	7	45957039	45957039	+	Splice Site	SNV	C	C	G	TCGA-B0-4837-01A-01D-1373-10
ITGB2	37	21	46321408	46321408	+	Missense Mutation	SNV	G	G	A	TCGA-B0-5088-01A-01D-1462-08
KCNMA1	37	10	78647067	78647067	+	Frame Shift Del	DEL	T	T	-	TCGA-AK-3450-01
KCNMA1	37	10	78669819	78669819	+	Missense Mutation	SNV	C	C	T	TCGA-B0-4813-01A-01D-1361-10
KCNMA1	37	10	78872137	78872137	+	Silent	SNV	C	C	A	TCGA-B8-4146-01B-11D-1669-08
KCNMA1	37	10	78709126	78709126	+	Splice Site	SNV	T	T	C	TCGA-CZ-5451-01A-01D-1501-10
KLF7	37	2	207988751	207988751	+	Silent	SNV	G	G	A	TCGA-B0-4822-01
LAMC1	37	1	183086460	183086460	+	Missense Mutation	SNV	G	G	T	TCGA-B0-5120-01A-01D-1421-08
LAMC1	37	1	183102612	183102612	+	Missense Mutation	SNV	G	G	A	TCGA-B8-4153-01B-11D-1669-08
LAMC1	37	1	183094629	183094629	+	Frame Shift Del	DEL	C	C	-	TCGA-DV-5565-01A-01D-1534-10
LOX	37	5	121411209	121411209	+	Missense Mutation	SNV	A	A	C	TCGA-BP-4331-01A-01D-1366-10
LOXL2	37	8	23217701	23217701	+	Missense Mutation	SNV	C	C	T	TCGA-B0-5077-01A-01D-1462-08
LOXL2	37	8	23167334	23167334	+	Missense Mutation	SNV	T	T	G	TCGA-BP-4964-01A-01D-1462-08

MARCKS	37	6	114181301	114181302	+	In Frame Ins	INS	-	-	TGAGGC	TCGA-A3-3319-01A-01D-0966-08
MARCKS	37	6	114178975	114178975	+	Missense Mutation	SNV	G	G	C	TCGA-B0-5713-01A-11D-1669-08
MARCKS	37	6	114181210	114181210	+	Frame Shift Del	DEL	A	A	-	TCGA-CZ-5459-01A-01D-1501-10
MARCKS	37	6	114181210	114181210	+	Frame Shift Del	DEL	A	A	-	TCGA-CZ-5465-01A-01D-1806-10
MET	37	7	116339927	116339927	+	Silent	SNV	G	G	T	TCGA-CJ-4916-01A-01D-1429-08
MET	37	7	116415115	116415115	+	Missense Mutation	SNV	T	T	A	TCGA-CW-6087-01A-11D-1669-08
MET	37	7	116411638	116411638	+	Silent	SNV	C	C	G	TCGA-CZ-5465-01A-01D-1806-10
MET	37	7	116380121	116380121	+	Missense Mutation	SNV	G	G	C	TCGA-EU-5907-01A-11D-1669-08
MVP	37	16	29853112	29853112	+	Missense Mutation	SNV	C	C	A	TCGA-B0-4842-01A-02D-1421-08
MVP	37	16	29841797	29841798	+	RNA	INS	-	-	C	TCGA-B0-5699-01
MVP	37	16	29858602	29858602	+	Missense Mutation	SNV	G	G	A	TCGA-CJ-5680-01A-11D-1534-10
MVP	37	16	29858549	29858549	+	Missense Mutation	SNV	G	G	A	TCGA-CZ-4853-01A-01D-1429-08
NDRG1	37	8	134270635	134270635	+	Splice Site	SNV	C	C	T	TCGA-B0-4815-01A-01D-1501-10
NDRG1	37	8	134296522	134296522	+	Silent	SNV	A	A	T	TCGA-BP-4355-01
NNMT	37	11	114183068	114183068	+	Missense Mutation	SNV	G	G	C	TCGA-B0-4712-01A-01D-1501-10
P4HA2	37	5	131543565	131543565	+	Nonsense Mutation	SNV	G	G	A	TCGA-B0-5099-01A-01D-1421-08
PGF	37	14	75416059	75416059	+	Splice Site	SNV	C	C	T	TCGA-A3-3367-01A-02D-1421-08
PKM	37	15	72495427	72495427	+	Missense Mutation	SNV	C	C	G	TCGA-B0-4841-01A-01D-1361-10
PLIN2	37	9	19116637	19116637	+	Missense Mutation	SNV	G	G	A	TCGA-AK-3458-01A-01D-1501-10
PLOD1	37	1	12010478	12010478	+	Nonsense Mutation	SNV	C	C	T	TCGA-CJ-4884-01

PLOD2	37	3	145820619	145820619	+	Missense Mutation	SNV	C	C	G	TCGA-B0-5119-01A-02D-1421-08
PLOD2	37	3	145788846	145788846	+	Missense Mutation	SNV	C	C	T	TCGA-BP-5198-01A-01D-1429-08
PLOD2	37	3	145804638	145804638	+	Missense Mutation	SNV	T	T	C	TCGA-CJ-4887-01A-01D-1373-10
PLOD2	37	3	145799589	145799589	+	Missense Mutation	SNV	A	A	T	TCGA-CJ-6033-01A-11D-1669-08
POSTN	37	13	38137418	38137418	+	RNA	SNV	T	T	A	TCGA-B0-5104-01
POSTN	37	13	38162097	38162097	+	Silent	SNV	G	G	A	TCGA-BP-4164-01
POSTN	37	13	38154095	38154095	+	Missense Mutation	SNV	C	C	A	TCGA-BP-5190-01A-01D-1429-08
POSTN	37	13	38143504	38143504	+	Silent	SNV	G	G	T	TCGA-CJ-4892-01
PTRF	37	17	40556892	40556892	+	Missense Mutation	SNV	C	C	A	TCGA-CZ-5455-01
RB1	37	13	49030485	49030485	+	Missense Mutation	SNV	G	G	A	TCGA-BP-4770-01A-01D-1501-10
RBPMS	37	8	30361883	30361883	+	Silent	SNV	A	A	G	TCGA-AK-3454-01A-02D-1361-10
RBPMS	37	8	30402150	30402150	+	Silent	SNV	T	T	C	TCGA-B0-5077-01A-01D-1462-08
RBPMS	37	8	30402171	30402171	+	Missense Mutation	SNV	C	C	G	TCGA-B0-5077-01A-01D-1462-08
RBPMS	37	8	30332299	30332299	+	Missense Mutation	SNV	G	G	T	TCGA-BP-4759-01
RPLP0	37	12	120636946	120636946	+	Missense Mutation	SNV	A	A	C	TCGA-BP-4998-01A-01D-1462-08
RPS11	37	19	50001200	50001200	+	Missense Mutation	SNV	A	A	G	TCGA-B0-5705-01A-11D-1534-10
RPS27A	37	2	55462091	55462091	+	Missense Mutation	SNV	A	A	T	TCGA-B0-4844-01A-01D-1361-10
RRAD	37	16	66956063	66956063	+	Missense Mutation	SNV	G	G	T	TCGA-BP-4165-01
RRAD	37	16	66956073	66956073	+	Missense Mutation	SNV	G	G	T	TCGA-CJ-4637-01A-02D-1386-10
RRAD	37	16	66956073	66956073	+	Missense Mutation	SNV	G	G	T	TCGA-CZ-4863-01A-01D-1501-10
SAP30	37	4	174292632	174292632	+	Missense Mutation	SNV	T	T	C	TCGA-B0-4817-01A-01D-1361-10

SCARB1	37	12	125267275	125267275	+	Missense Mutation	SNV	A	A	G	TCGA-A3-3385-01
SCARB1	37	12	125279747	125279747	+	Missense Mutation	SNV	C	C	A	TCGA-CJ-4881-01A-01D-1373-10
SHMT2	37	12	57627609	57627609	+	Missense Mutation	SNV	G	G	A	TCGA-A3-3308-01A-01D-0966-08
SHMT2	37	12	57625269	57625269	+	Missense Mutation	SNV	C	C	A	TCGA-B0-4817-01A-01D-1361-10
SHMT2	37	12	57626066	57626066	+	Frame Shift Del	DEL	T	T	-	TCGA-BP-4976-01A-01D-1462-08
SLAMF7	37	1	160719769	160719769	+	Missense Mutation	SNV	A	A	G	TCGA-B0-5095-01A-01D-1421-08
SLAMF7	37	1	160718025	160718025	+	Missense Mutation	SNV	G	G	A	TCGA-BP-4963-01A-01D-1462-08
SLC16A3	37	17	80195666	80195666	+	Missense Mutation	SNV	C	C	A	TCGA-BP-4775-01A-01D-1366-10
SLC16A3	37	17	80194627	80194627	+	Silent	SNV	G	G	C	TCGA-CZ-5459-01A-01D-1501-10
SLC6A8	37	X	152957540	152957540	+	Missense Mutation	SNV	A	A	G	TCGA-B0-4828-01A-01D-1361-10
SLC6A8	37	X	152954167	152954167	+	Silent	SNV	C	C	A	TCGA-B0-5110-01
SLC6A8	37	X	152956789	152956789	+	Missense Mutation	SNV	T	T	A	TCGA-B8-4151-01A-01D-1806-10
SPARC	37	5	151049279	151049279	+	Frame Shift Del	DEL	C	C	-	TCGA-B8-5550-01
STAT6	37	12	57499020	57499021	+	Frame Shift Ins	INS	-	-	G	TCGA-A3-3323-01A-01D-0966-08
STAT6	37	12	57499020	57499021	+	Frame Shift Ins	INS	-	-	G	TCGA-B0-5099-01A-01D-1421-08
STAT6	37	12	57493192	57493192	+	Missense Mutation	SNV	G	G	T	TCGA-B8-5163-01A-01D-1421-08
STAT6	37	12	57501489	57501489	+	Missense Mutation	SNV	A	A	C	TCGA-BP-4351-01A-01D-1366-10
STC1	37	8	23702339	23702339	+	Silent	SNV	G	G	T	TCGA-B0-4839-01A-01D-1373-10
TNS1	37	2	218713396	218713396	+	Missense Mutation	SNV	C	C	G	TCGA-B2-5633-01A-01D-1534-10
TNS1	37	2	218673368	218673368	+	Missense Mutation	SNV	A	A	T	TCGA-BP-4329-01A-02D-1366-10

TNS1	37	2	218686541	218686541	+	Missense Mutation	SNV	A	A	G	TCGA-BP-4775-01A-01D-1366-10
TNS1	37	2	218713105	218713105	+	Missense Mutation	SNV	C	C	A	TCGA-BP-5004-01A-01D-1462-08
TRIB3	37	20	377225	377225	+	Frame Shift Del	DEL	C	C	-	TCGA-CJ-4636-01
TSC2	37	16	2132493	2132493	+	Missense Mutation	SNV	G	G	A	TCGA-A3-3322-01
TSC2	37	16	2112006	2112006	+	Missense Mutation	SNV	G	G	T	TCGA-B0-4712-01A-01D-1501-10
TSC2	37	16	2131631	2131631	+	Missense Mutation	SNV	C	C	A	TCGA-B0-5709-01A-11D-1534-10
TSC2	37	16	2126068	2126068	+	Splice Site	SNV	G	G	A	TCGA-BP-4801-01A-02D-1421-08
TSC2	37	16	2126070	2126070	+	Missense Mutation	SNV	T	T	A	TCGA-BP-4801-01A-02D-1421-08
TSC2	37	16	2134519	2134519	+	Silent	SNV	C	C	G	TCGA-CZ-4859-01A-02D-1429-08
UBC	37	12	125397153	125397153	+	Missense Mutation	SNV	T	T	C	TCGA-A3-3387-01A-01D-1534-10
UBC	37	12	125397187	125397187	+	Silent	SNV	G	G	A	TCGA-AS-3778-01A-01D-0966-08
UBC	37	12	125398050	125398052	+	In_Frame Del	DEL	TGA	TGA	-	TCGA-B0-5710-01A-11D-1669-08
VCAM1	37	1	101200121	101200121	+	Missense Mutation	SNV	T	T	G	TCGA-B0-5102-01A-01D-1421-08
VEGFA	37	6	43742101	43742101	+	Silent	SNV	A	A	G	TCGA-B0-4818-01A-01D-1501-10

Tab. S8: Shared somatic variants in the TCGA patient cohort.

Gene	Number of shared variants	Variant	Patients	Number of patients
STAT6	1	57499020	TCGA-A3-3323,TCGA-B0-5099	2
MARCKS	1	114181210	TCGA-CZ-5459,TCGA-CZ-5465	2
RRAD	1	66956073	TCGA-CJ-4637,TCGA-CZ-4863	2
FCGR1A	1	149763191	TCGA-CJ-4643,TCGA-CJ-5675,TCGA-CW-5589	3
HLA-DRB5	1	32497905	TCGA-BP-4176,TCGA-BP-4759	2

Tab. S9: Coding variants in the candidate genes TSC2, RB1, and MET in patient cohort 1.

Gene	Chr	Coordinate	Variant	Type	Median alt. variant freq. (range)	Number of samples (percent)	Protein pos.	Amino acid subst.	Codon	rs number
TSC2	16	2103409	C>C/T	snv	2	1 (1.9)	98	R/W	Cgg/Tgg	rs372321790
		2104327	T>T/A	snv	7.3	1 (1.9)	123	F/I	Ttc/Atc	
		2104344	G>G/T	snv	3.4	1 (1.9)	128	K/N	aaG/aaT	rs748095129
		2104408	G>G/T	snv	4.9	1 (1.9)	150	G/W	Ggg/Tgg	
		2105442	C>C/T	snv	48.4	1 (1.9)	174	S/L	tCg/tTg	rs747538587
		2106729	CG>CG/C	del	6.7	1 (1.9)	245	R/X	cGc/cc	
		2106733	C>C/T	snv	12.8	1 (1.9)	246	T/I	aCc/aTc	
		2106745	A>A/T	snv	5.1	1 (1.9)	250	K/M	aAg/aTg	
		2106757	A>A/T	snv	4.1	1 (1.9)	254	E/V	gAg/gTg	
		2107149	T>T/C	snv	2.5	1 (1.9)	273	I/T	aTc/aCc	
		2110795	G>G/A	snv	46.9 (44.3-49.4)	2 (3.8)	367	R/Q	cGg/cAg	rs1800725
		2121512	C>C/A	snv	4.7	1 (1.9)	614	A/D	gCc/gAc	rs45454398
		2121614	A>A/T	snv	8.7	1 (1.9)	648	Y/F	tAc/tTc	
		2122248	G>G/T	snv	2.7	1 (1.9)	702	D/Y	Gac/Tac	
		2125856	G>G/T	snv	7.5	1 (1.9)	868	V/L	Gtg/Ttg	
		2126540	G>G/T	snv	2.5	1 (1.9)	931	D/Y	Gac/Tac	
		2129181	A>A/G	snv	10.2	1 (1.9)	1039	T/A	Acg/Gcg	
		2130243	CG>CG/C	del	3.4	1 (1.9)	1159	R/X	cGg/cg	
		2130250	C>C/T	snv	2.6	1 (1.9)	1161	A/V	gCa/gTa	rs397514901
		2131791	C>C/A	snv	2.1	1 (1.9)	1269	S/Y	tCc/tAc	
		2132438	G>G/T	snv	3.1	1 (1.9)	1272	V	gtG/gtT	
		2134229	T>T/G	snv	2.6	1 (1.9)	1336	S/A	Tcg/Gcg	
		2134248	A>A/T	snv	2.8	1 (1.9)	1342	Q/L	cAg/cTg	
		2134377	C>C/A	snv	3.9	1 (1.9)	1385	S/Y	tCc/tAc	
		2134432	C>C/CA	ins	4.9	1 (1.9)	0	-/X	-/A	
		2134442	G>G/A	snv	3.5	1 (1.9)	1407	V/M	Gtg/Atg	rs45517332
		2134569	C>C/T	snv	5.2	1 (1.9)	1449	S/F	tCc/tTc	rs759004251
		2134581	C>C/T	snv	6.5	1 (1.9)	1453	P/L	cCc/cTc	rs748182471
		2134616	G>G/T	snv	4.1	1 (1.9)	1465	D/Y	Gac/Tac	
		2135288	C>C/T	snv	2.5	1 (1.9)	1543	H/Y	Cac/Tac	
		2135300	G>G/T	snv	3.1	1 (1.9)	1547	V/F	Gtc/Ttc	
		2136332	G>G/A	snv	2.3	1 (1.9)	1601	G/S	Ggt/Agt	
		2136837	G>G/T	snv	3.1	1 (1.9)	1652	D/Y	Gac/Tac	
		2138508	G>G/C	snv	47.3	1 (1.9)	1774	S/T	aGc/aCc	rs9209
		2138662	G>G/T	snv	2.6	1 (1.9)	3' UTR			
		2138670	TAA>TAA/T	del	49.5 (35.2-97.5)	6 (11.3)	3' UTR			rs36032671; rs71744655
RB1	13	49027168	C>C/T	snv	3.5	1 (1.9)	579	R/*	Cga/Tga	rs121913305
MET	7	116340086	A>A/G	snv	65.9	1 (1.9)	316	I/M	atA/atG	rs35225896
		116340156	G>G/T	snv	5.3	1 (1.9)	340	D/Y	Gac/Tac	
		116340209	G>G/T	snv	3.8	1 (1.9)	357	M/I	atG/atT	

		116340262	A>A/G	snv	50.4 (41.3-53.9)	3 (5.7)	375	N/S	aAc/aGc	rs33917957
		116340266	G>G/T	snv	2.9	1 (1.9)	376	K/N	aaG/aaT	
		116371891	C>C/A	snv	2.5	1 (1.9)	457	T/K	aCa/aAa	
		116399419	G>G/T	snv	2.2	1 (1.9)	765	V/F	Gtc/Ttc	
		116411923	C>C/T	snv	50.7 (48.0-53.4)	2 (3.8)	988	R/C	Cgc/Tgc	rs34589476
		116411990	C>C/T	snv	37.7 (36.1-39.3)	2 (3.8)	1010	T/I	aCt/aTt	rs56391007
		116414949	T>T/G	snv	25.6	1 (1.9)	1033	S/A	Tca/Gca	rs539345989

Tab. S10: Overview of candidate genes.

Gene expression is given in log_2 FPKM-UQ.

Entrez ID	Gene Symbol	Median expr. tumor	Median expr. normal	FC tumor/ normal [log_2]	p-value tumor/ normal (Wilcoxon rank-sum)	FC paired tumor/ normal [log_2]	p-value paired tumor/ normal (Wilcoxon signed-rank)	CV [%]	Somatic mut. (TCGA)	MHC class I peptides	MHC class II peptides	Nr. of patients with class I peptide	Nr. of patients with class II peptide	Immunogenic peptides	Patients with variants (cohort 1)	Number diff. variants (cohort 1)
19	ABCA1	17.85	16.19	1.67	8.90E-36	1.66	1.31E-12	4.02	8	1	0	3	0			
25890	ABI3BP	17.30	16.65	0.65	2.75E-05	0.61	7.52E-04	7.75	1	1	0	3	0			
47	ACLY	20.99	19.46	1.53	2.26E-37	1.61	1.15E-12	2.53	5	1	0	3	0			
60	ACTB	24.70	24.21	0.48	5.87E-10	0.31	8.80E-04	2.04	0	0	1	0	3			
133	ADM	20.76	17.61	3.16	1.04E-37	2.89	4.19E-12	4.10	0	1	0	3	0			
375790	AGRN	20.07	19.86	0.22	3.86E-05	0.18	7.16E-03	3.13	5	2	3	9	11			
226	ALDOA	22.87	21.66	1.22	7.84E-33	1.30	3.20E-11	2.52	2	4	0	15	0			
230	ALDOC	19.54	16.57	2.97	9.77E-30	2.41	3.10E-10	6.57	0	2	0	7	0			
51129	ANGPTL4	22.87	16.56	6.31	1.53E-39	6.10	1.15E-12	4.33	2	2	0	5	0	2		
302	ANXA2	20.18	19.48	0.70	6.06E-15	0.90	1.63E-09	3.35	1	1	2	5	7			
307	ANXA4	20.76	18.85	1.91	2.74E-38	2.20	1.20E-12	3.30	2	0	2	0	10			
308	ANXA5	21.62	21.11	0.51	1.07E-18	0.63	1.08E-10	1.81	0	0	1	0	3			
372	ARCN1	19.99	19.89	0.09	2.67E-03	0.20	1.23E-06	1.71	3	1	0	6	0			
567	B2M	24.56	23.01	1.55	1.26E-33	1.45	2.25E-12	2.44	1	0	1	0	3			
633	BGN	23.11	21.88	1.23	2.49E-16	0.56	3.74E-04	5.19	1	0	1	0	5			
664	BNIP3	20.32	18.65	1.67	7.86E-35	1.68	2.69E-12	3.66	0	1	0	3	0			
689	BTF3	21.70	21.18	0.52	1.25E-23	0.58	2.18E-11	2.05	0	1	0	3	0			
718	C3	21.94	16.57	5.37	4.38E-35	5.11	1.44E-12	6.51	7	6	3	18	10			
821	CANX	22.16	21.60	0.56	2.82E-15	0.79	5.70E-12	2.62	1	0	2	0	8			
284119	CAVIN1	21.35	20.40	0.96	6.31E-25	0.87	7.46E-10	2.74	1	1	0	3	0			
595	CCND1	21.94	19.62	2.32	1.18E-37	2.29	1.15E-12	3.92	0	2	0	11	0			
1050	CEBPA	16.28	15.04	1.24	6.68E-22	1.61	4.63E-10	6.60	3	1	0	3	0			
1277	COL1A1	20.44	17.24	3.20	3.96E-24	2.47	7.17E-10	8.36	3	1	0	3	0			
1278	COL1A2	20.22	18.32	1.90	1.19E-17	1.52	3.79E-08	6.73	5	0	1	0	4			
1284	COL4A2	21.62	19.85	1.77	5.10E-31	1.66	1.30E-11	3.77	4	1	2	4	4			
1292	COL6A2	20.75	18.51	2.23	4.47E-31	1.74	2.54E-10	5.00	5	1	1	3	3			
1509	CTSD	23.18	22.91	0.27	1.21E-03	0.35	1.72E-04	3.47	0	1	4	6	9			
54541	DDIT4	21.56	19.00	2.56	8.34E-34	2.23	1.97E-12	4.12	0	1	0	3	0			

10085	EDIL3	18.63	17.60	1.03	4.95E-12	1.18	9.26E-07	6.84	3	0	2	0	4			
1915	EEF1A1	24.63	24.41	0.23	1.61E-02	0.22	2.74E-03	2.38	3	0	4	0	5			
112399	EGLN3	20.85	16.17	4.68	1.51E-39	4.43	1.20E-12	3.45	0	4	0	19	0	5		
1975	EIF4B	20.93	20.60	0.32	2.28E-07	0.44	2.60E-07	2.81	0	1	0	3	0			
2209	FCGR1A	15.81	12.46	3.35	6.62E-37	3.47	1.15E-12	7.23	3	0	2	0	4			
2335	FN1	20.87	19.14	1.73	4.95E-26	2.03	7.10E-12	5.54	6	0	8	0	17			
6624	FSCN1	19.14	17.66	1.47	3.74E-30	1.27	1.27E-10	4.77	3	1	0	3	0			
2521	FUS	19.30	18.79	0.51	1.30E-15	0.32	6.51E-07	2.29	3	1	0	3	0			
2548	GAA	19.49	19.21	0.28	5.51E-07	0.37	4.13E-07	3.14	2	0	1	0	4			
9514	GAL3ST1	20.21	17.19	3.02	3.10E-38	3.17	1.80E-12	5.30	2	1	0	3	0			
2597	GAPDH	24.94	23.45	1.49	1.15E-37	1.59	2.25E-12	2.69	0	0	1	0	3			
2745	GLRX	18.53	17.94	0.59	1.12E-05	0.55	1.55E-04	4.16	0	0	1	0	3			
3105	HLA-A	24.18	22.41	1.77	6.12E-38	1.86	1.25E-12	2.60	2	0	1	0	3			
3106	HLA-B	24.60	22.39	2.21	3.10E-38	2.14	1.20E-12	2.54	0	1	2	3	8			
3107	HLA-C	23.85	22.49	1.35	1.49E-34	1.47	1.44E-12	2.56	2	0	1	0	5			
3108	HLA-DMA	20.35	18.86	1.49	4.10E-35	1.55	1.64E-12	3.48	0	1	0	3	0			
3111	HLA-DOA	18.71	17.52	1.19	7.01E-19	1.55	9.90E-11	6.03	1	1	0	4	0			
3122	HLA-DRA	24.59	22.75	1.83	1.83E-32	2.02	1.57E-12	3.53	1	0	1	0	3			
3123	HLA-DRB1	23.74	22.09	1.65	1.52E-32	1.84	1.31E-12	3.36	1	0	2	0	4			
3127	HLA-DRB5	21.94	20.53	1.41	1.14E-18	1.74	2.25E-12	4.84	2	0	1	0	4			
3133	HLA-E	23.50	22.31	1.19	3.69E-36	1.24	1.31E-12	1.91	3	0	1	0	5			
3162	HMOX1	21.44	19.02	2.42	2.21E-37	3.11	1.44E-12	3.73	2	2	0	8	0			
3178	HNRNPA1	21.32	20.88	0.44	2.75E-22	0.43	1.15E-09	1.71	0	1	0	3	0			
26353	HSPB8	20.86	18.50	2.36	9.77E-37	2.79	1.15E-12	3.51	0	1	0	3	0			
5654	HTRA1	21.40	20.67	0.73	7.14E-12	0.96	2.51E-07	3.81	0	1	1	4	4			
3429	IFI27	19.24	17.92	1.33	2.45E-23	1.39	1.69E-10	5.39	1	1	0	3	0			
10437	IFI30	13.92	12.01	1.90	2.21E-27	1.31	1.91E-10	9.65	2	0	1	0	5			
3486	IGFBP3	23.50	19.53	3.98	4.83E-39	3.74	1.25E-12	4.31	1	1	6	3	8			
3689	ITGB2	18.96	16.21	2.75	1.36E-37	2.82	1.80E-12	4.24	1	0	1	0	3			
3778	KCNMA1	16.06	13.26	2.80	4.71E-28	2.52	9.12E-11	8.47	4	1	0	3	0			
8609	KLF7	17.20	16.65	0.55	4.11E-12	0.55	5.72E-07	3.75	1	1	0	3	0			
3875	KRT18	21.30	20.57	0.73	1.41E-11	0.79	4.71E-07	4.07	0	8	0	17	0			
3856	KRT8	20.85	20.35	0.50	6.08E-05	0.47	3.74E-04	4.25	0	2	0	7	0			
3915	LAMC1	19.86	19.16	0.70	1.55E-15	0.84	4.45E-10	3.06	3	0	1	0	3			
3959	LGALS3BP	21.47	21.25	0.22	2.43E-03	0.49	1.09E-04	4.31	0	0	1	0	5			
4015	LOX	19.89	16.23	3.66	2.80E-35	4.23	1.80E-12	8.15	1	1	0	3	0			
4017	LOXL2	18.56	15.35	3.21	1.41E-35	3.20	2.25E-12	5.65	2	1	0	3	0			

4082	MARCKS	20.09	18.93	1.16	4.13E-30	1.24	1.62E-11	2.89	4	0	1	0	3			
4233	MET	19.82	18.69	1.13	4.07E-26	1.48	1.97E-12	3.78	4	1	0	4	0		13	10
9961	MVP	20.40	19.62	0.78	6.25E-25	1.01	4.49E-11	2.62	4	1	0	3	0			
10135	NAMPT	18.05	17.72	0.33	7.33E-05	0.45	9.58E-05	4.32	0	1	0	4	0			
10397	NDRG1	22.45	21.04	1.40	3.27E-33	1.51	3.07E-11	3.13	2	3	0	10	0			
4837	NNMT	22.40	17.23	5.17	5.99E-38	4.93	1.15E-12	5.24	1	3	0	10	0	2		
4869	NPM1	21.90	20.98	0.92	5.65E-31	0.96	3.51E-12	2.59	0	0	1	0	5			
8974	P4HA2	18.31	16.57	1.74	3.70E-28	1.75	2.09E-11	4.03	1	1	0	5	0	0		
5036	PA2G4	19.42	18.99	0.43	1.08E-23	0.44	2.09E-11	1.90	0	1	0	3	0			
5066	PAM	19.80	19.33	0.46	9.75E-15	0.63	1.17E-10	2.66	0	1	0	3	0			
8682	PEA15	21.18	20.47	0.72	8.83E-27	0.73	1.30E-11	1.93	0	0	1	0	3			
5214	PFKP	21.35	18.98	2.38	1.44E-37	2.25	2.15E-12	2.60	0	2	0	7	0	0		
5228	PGF	18.43	14.36	4.07	4.72E-38	3.84	1.15E-12	9.51	1	1	0	5	0			
5315	PKM	22.47	21.59	0.88	1.80E-13	0.89	1.29E-08	2.21	1	2	0	6	0			
123	PLIN2	22.15	18.57	3.58	7.82E-37	3.17	2.69E-12	4.84	1	12	1	27	3			
5351	PLOD1	20.20	18.81	1.39	1.03E-35	1.52	7.74E-12	3.41	1	1	0	3	0			
5352	PLOD2	19.58	18.04	1.54	6.42E-32	1.92	2.46E-12	4.99	4	2	0	8	0			
10631	POSTN	18.28	16.89	1.39	2.35E-13	2.11	6.02E-06	8.56	4	0	1	0	4			
5925	RB1	18.63	18.16	0.46	8.69E-13	0.56	2.79E-09	2.62	1	1	0	3	0		1	1
11030	RBPMS	18.80	18.43	0.37	9.23E-09	0.15	2.51E-02	3.00	3	1	0	3	0			
6175	RPLP0	22.79	21.76	1.04	1.54E-30	1.07	2.18E-11	2.85	1	1	0	3	0			
6205	RPS11	24.10	23.24	0.85	1.01E-27	0.79	5.77E-11	2.40	1	0	1	0	3			
6223	RPS19	21.96	20.55	1.42	1.53E-34	1.37	2.00E-11	3.56	0	1	0	3	0			
6224	RPS20	22.43	21.44	0.99	4.87E-27	0.89	1.42E-11	3.28	0	0	1	0	3			
6233	RPS27A	21.68	21.16	0.52	1.86E-21	0.57	2.82E-11	2.15	1	0	1	0	3			
6236	RRAD	19.32	17.50	1.82	3.06E-20	1.35	6.66E-08	7.01	3	2	0	9	0			
10670	RRAGA	20.70	20.44	0.25	9.87E-09	0.25	1.12E-04	2.11	0	1	0	6	0			
8819	SAP30	18.38	15.73	2.66	8.97E-40	2.69	1.15E-12	2.82	1	1	0	5	0			
949	SCARB1	19.56	15.18	4.38	6.95E-40	4.25	1.15E-12	4.96	2	1	0	3	0			
6319	SCD	20.87	17.14	3.73	7.85E-38	3.73	1.15E-12	4.34	0	2	0	7	0			
6472	SHMT2	20.08	17.92	2.16	2.69E-39	2.28	1.15E-12	2.93	3	1	0	3	0			
57823	SLAMF7	16.07	13.11	2.96	2.74E-32	3.23	5.22E-12	7.93	2	0	3	0	6			
9123	SLC16A3	19.48	15.97	3.51	1.79E-39	3.48	1.64E-12	3.64	2	2	0	5	0			
6535	SLC6A8	20.63	19.06	1.57	2.48E-26	1.73	1.38E-10	5.45	3	1	0	7	0			
6629	SNRPB2	18.71	18.55	0.17	5.80E-06	0.26	2.45E-05	1.96	0	1	0	3	0			
6678	SPARC	23.62	21.50	2.12	6.00E-35	2.06	1.31E-12	3.10	1	0	1	0	3			
6778	STAT6	19.75	19.19	0.57	1.64E-26	0.45	9.82E-10	1.84	4	1	0	3	0			
8987	STBD1	14.86	13.79	1.07	3.66E-27	1.29	1.25E-12	4.33	0	1	0	3	0			
6781	STC1	20.17	19.69	0.48	9.22E-04	0.38	3.34E-02	5.11	1	1	0	3	0			

54732	TMED9	20.92	20.19	0.73	8.47E-26	0.92	1.15E-11	2.64	0	1	0	3	0			
7145	TNS1	20.40	20.05	0.34	1.33E-06	0.35	1.01E-03	3.43	4	2	0	6	0			
57761	TRIB3	18.14	15.20	2.94	1.52E-36	2.99	2.06E-12	7.49	1	1	0	4	0			
7249	TSC2	17.27	17.05	0.22	9.70E-11	0.05	3.90E-02	2.56	5	1	0	4	0		30	37
7311	UBA52	20.96	20.41	0.55	1.90E-24	0.63	6.27E-11	2.32	0	0	1	0	3			
7316	UBC	22.48	21.53	0.95	5.97E-32	0.93	3.07E-12	1.93	3	0	1	0	3			
7412	VCAM1	21.11	19.08	2.03	1.48E-28	2.26	7.41E-12	4.72	1	1	4	3	9			
7422	VEGFA	21.40	17.78	3.63	2.85E-39	3.30	1.37E-12	4.23	1	1	0	9	0			
7431	VIM	23.83	20.88	2.95	1.60E-39	2.85	1.15E-12	2.34	0	1	0	4	0			

Abbreviations: FC, fold-change; CV, coefficient of variation; NA, not available

Fig. S1:

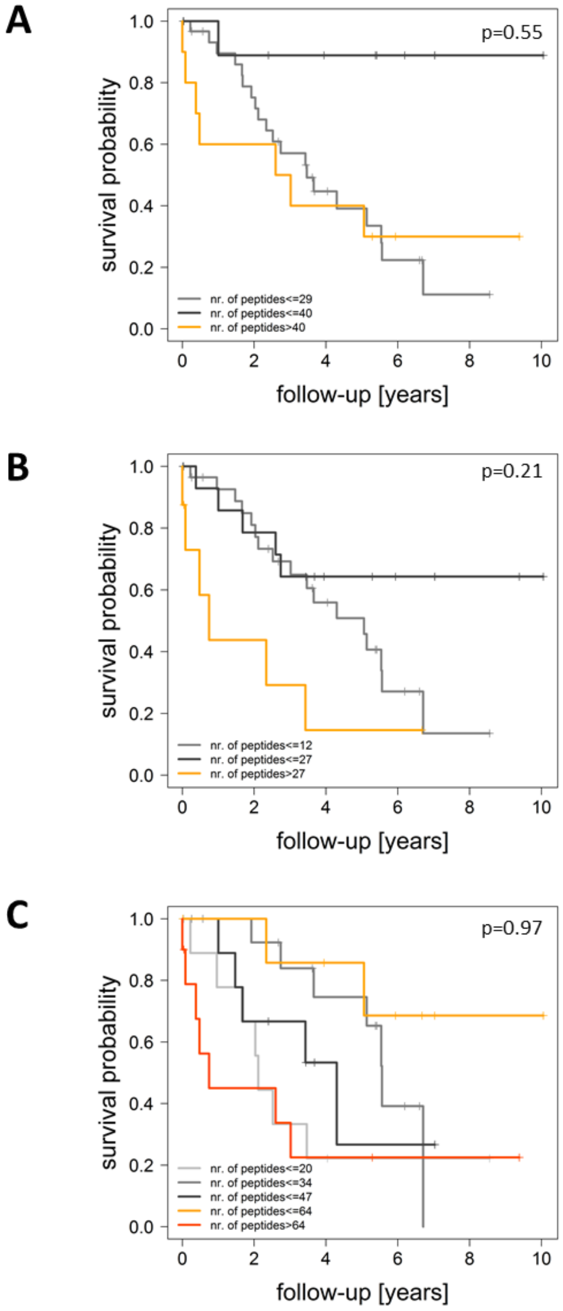

Fig. S1: Peptide presentation and overall patient survival in patient cohort 1.

Overall survival of patients and the presence of **A** frequent ccRCC-associated HLA class I presented peptides; **B** frequent ccRCC-associated HLA class II presented peptides; and **C** combined frequent ccRCC-associated HLA class I and class II presented peptides**.** The conditional interference tree model was used to determine the optimal cutoffs with respect to overall survival using the R packages survival [1] and party [2]. P-values were computed from absolute peptide numbers by Wald test.

Fig. S2:

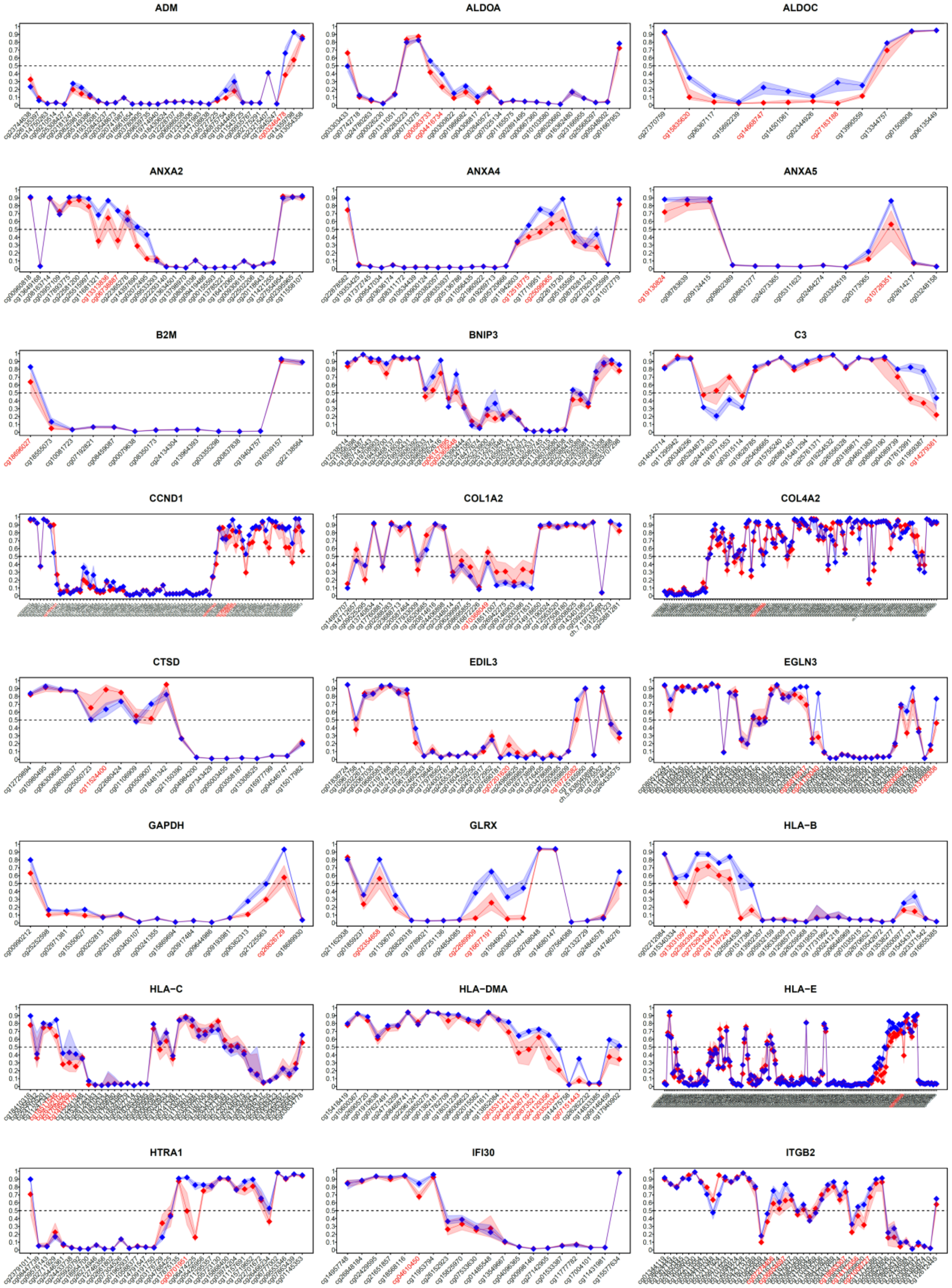
ADM
ALDOA
ALDOC
ANXA2
ANXA4
ANXA5
B2M
BNIP3
C3
CCND1
COL1A2
COL4A2
CTSD
EDIL3
EGLN3
GAPDH
GLRX
HLA−B
HLA−C
HLA−DMA
HLA−E
HTRA1
IFI30
ITGB2

Fig. S2 (continued):

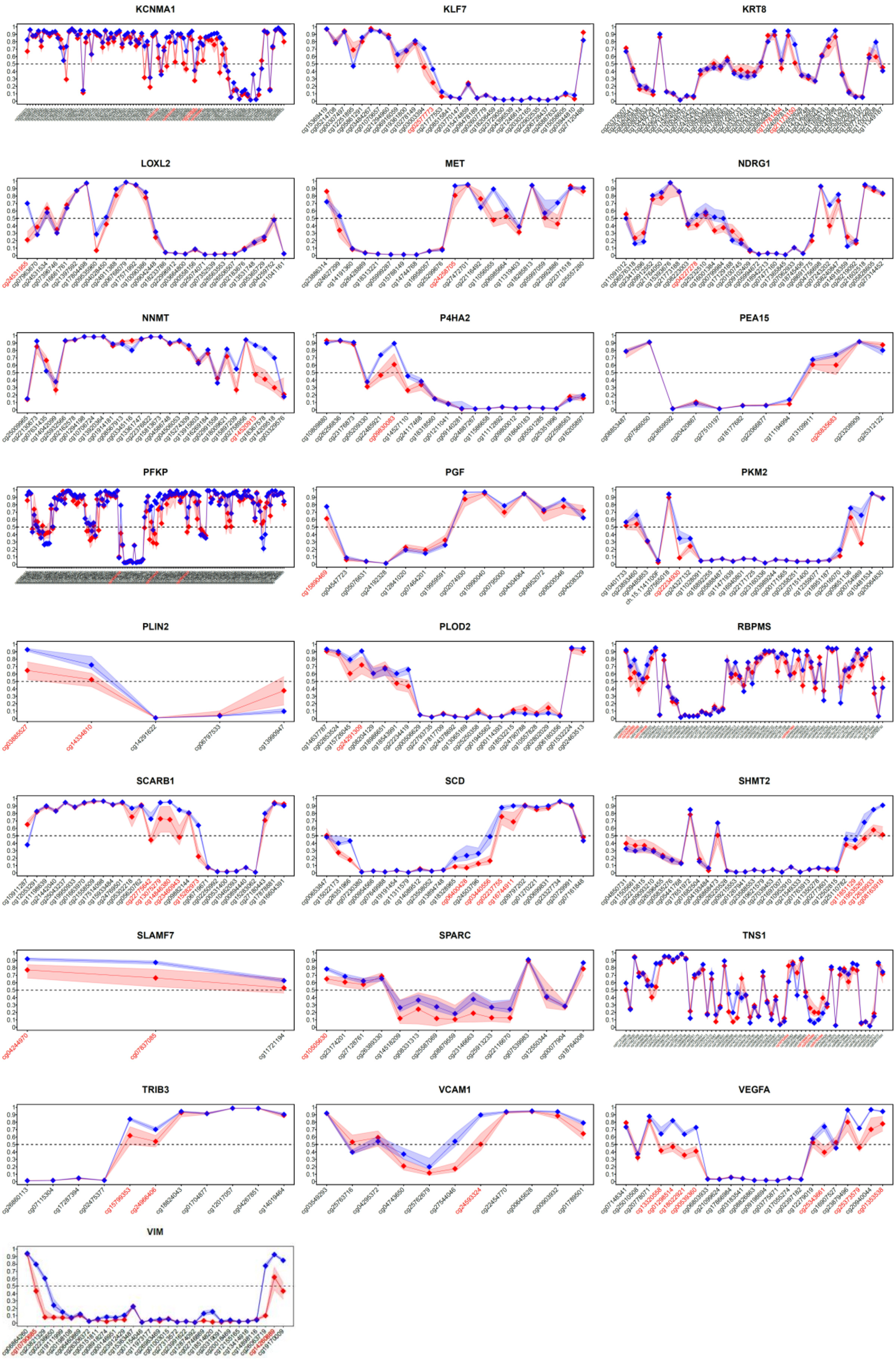

Fig. S2: DNA methylation profiles of differentially methylated genes in the TCGA patient cohort. The plots show the median methylation levels (curves) in tumors (red) and non-tumor tissues (blue) together with the 25 % and 75 % quantile ranges (shaded areas). The sites are ordered according to their genomic position in 5'->3' direction. Differentially methylated sites that correlate significantly negative ($R_s \leq -0.3$, $p < 0.05$) with gene expression of the associated gene are printed in red.

Fig. S3:

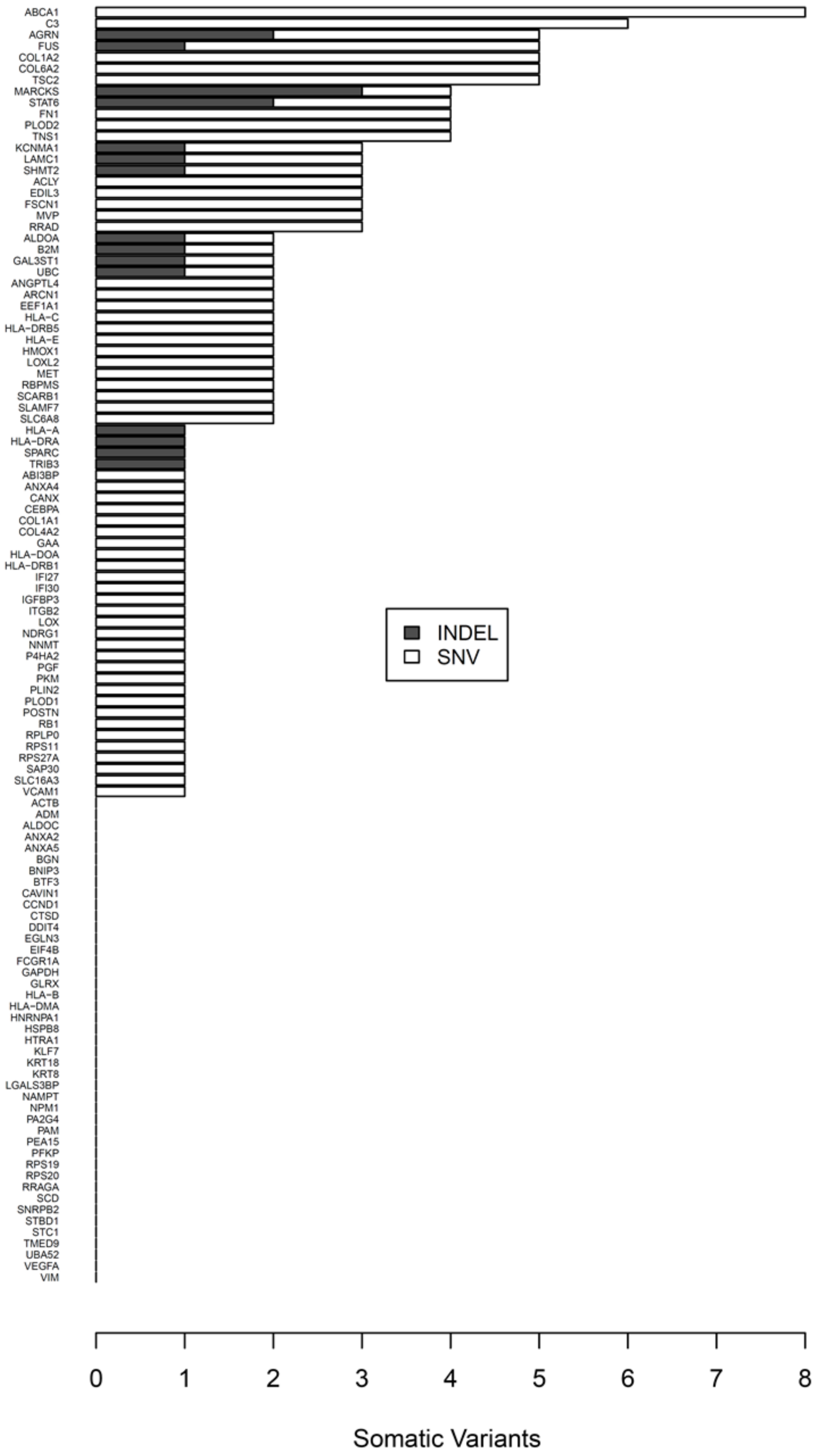

Fig. S3: Somatic variants in candidate genes in 392 samples of the TCGA patient cohort. The bars indicate the number of tumors with somatic point mutations or indels resulting in protein alterations of the corresponding genes.

Fig. S4:

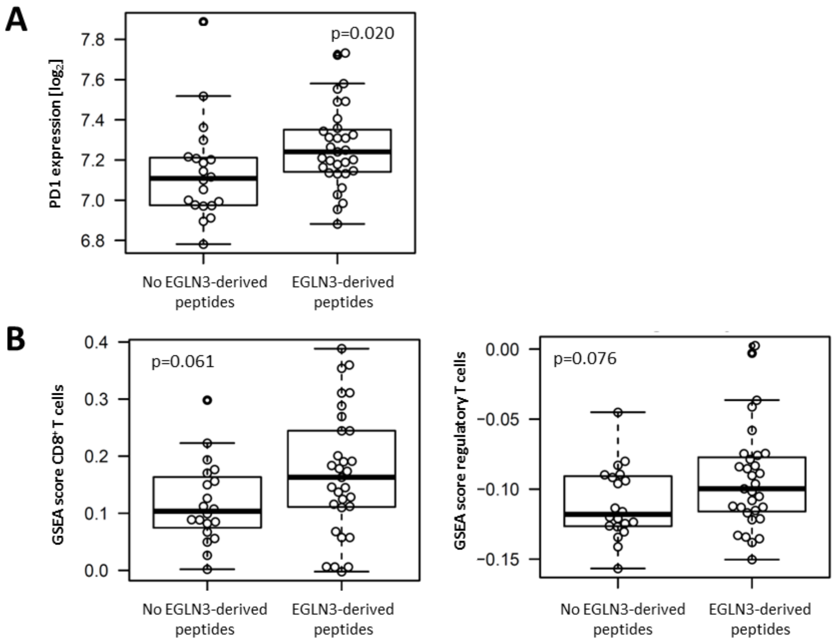

Fig. S4: EGLN3-derived peptides and tumor immune infiltrate in patient cohort 1. A PD1 expression in tumor tissues of patients without EGLN3-derived peptides and with at least one EGLN3-derived peptide. **B** GSEA scores for CD8+ T cell infiltrate (left) and regulatory T cell infiltrate (right) in patients without and with at least one EGLN3-derived peptide. Immune signatures were taken from Rooney *et al.* 2015 [3]. Other signatures that were assessed are: B cells, macrophages, neutrophils, NK cells, pDCs, MHC class I, co-stimulation APC, co-stimulation T cell, co-inhibition APC, co-inhibition T cell, type I interferon response, type II interferon response, and cytolytic activity [3]. None of those correlated significantly with the presence of EGLN3-derived peptides. Wilcoxon rank-sum test p-values are shown in the plots.

Fig. S5:

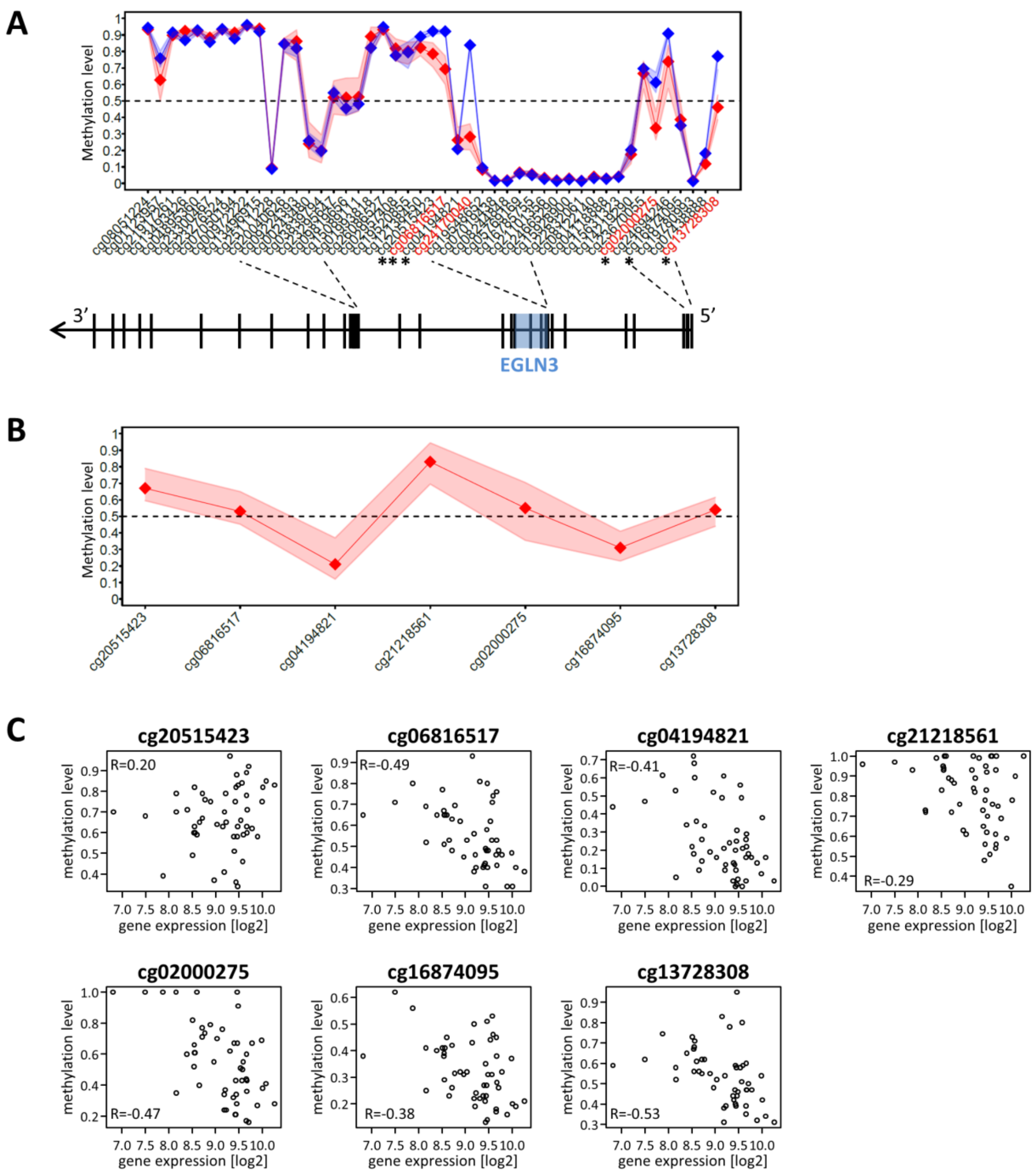

Fig. S5: DNA methylation of the EGLN3 gene region. A Methylation profile in tumor (red) and non-tumor tissue (blue) in the TCGA patient cohort (enlarged from Fig. S2). The scheme below depicts the *EGLN3* gene region. Sites that were chosen for further analysis in patient cohort 1 are marked by an asterisk. **B** Methylation profiles in patient cohort 1 analyzed by MALDI-TOF MS of bisulfite treated DNA as described previously [4]. The site cg21218561 was chosen for analysis in the cohort, in addition to the sites marked above. **C** Correlation plots of *EGLN3* gene expression and DNA methylation at the indicated CpG sites in patient cohort 1. Spearman correlation coefficients are printed in the graphs.

Fig. S6:

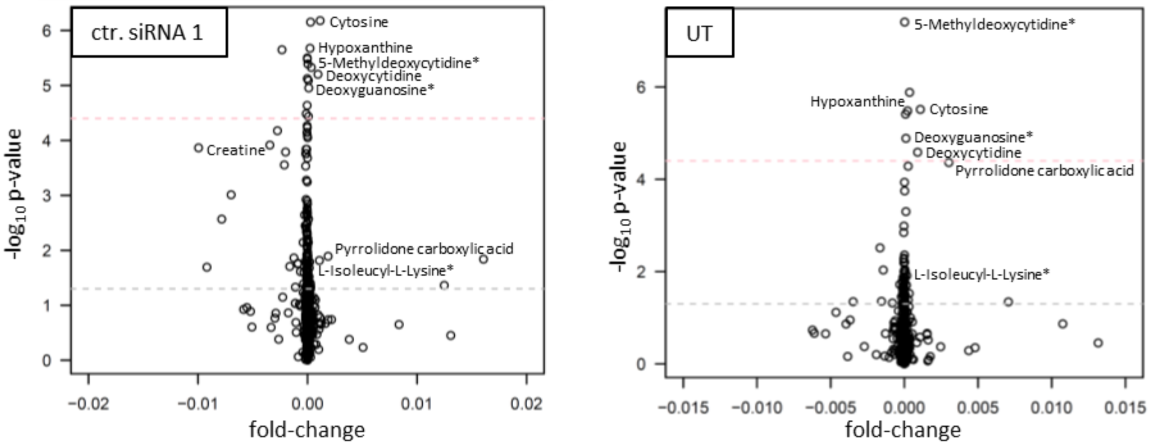

Fig. S6: Differentially regulated metabolites in EGLN3 knockdown 786-O cells. Features were measured in positive ionization modes and assigned to specific metabolites if possible, using the in-house metabolite library [5]. Metabolites marked by an asterisk are assigned by their molecular formula using the PubChem database [6]. The plot on the left shows fold-changes between *EGLN3* knockdown cells and cells transfected with a control non-targeting siRNA pool. The plot on the right shows fold-changes between *EGLN3* knockdown cells and untreated cells. The intersected lines indicate the significance thresholds of unadjusted (grey) and adjusted (pink) p-values.

Fig. S7:

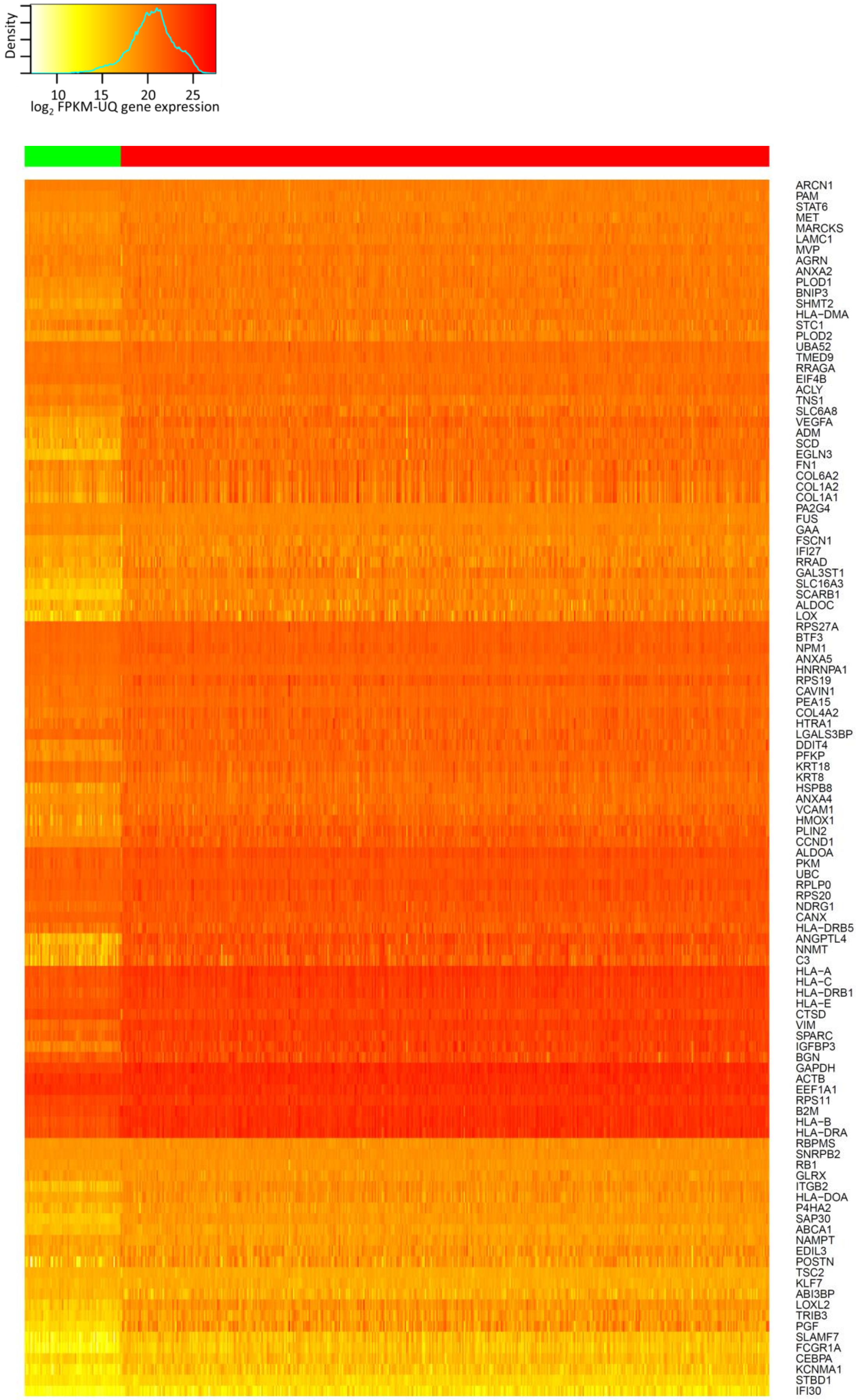
Density
10
15
20
25
log2 FPKM-UQ gene expression
ARCN1
PAM
STAT6
MET
MARCKS
LAMC1
MVP
AGRN
ANXA2
PLOD1
BNIP3
SHMT2
HLA−DMA
STC1
PLOD2
UBA52
TMED9
RRAGA
EIF4B
ACLY
TNS1
SLC6A8
VEGFA
ADM
SCD
EGLN3
FN1
COL6A2
COL1A2
COL1A1
PA2G4
FUS
GAA
FSCN1
IFI27
RRAD
GAL3ST1
SLC16A3
SCARB1
ALDOC
LOX
RPS27A
BTF3
NPM1
ANXA5
HNRNPA1
RPS19
CAVIN1
PEA15
COL4A2
HTRA1
LGALS3BP
DDIT4
PFKP
KRT18
KRT8
HSPB8
ANXA4
VCAM1
HMOX1
PLIN2
CCND1
ALDOA
PKM
UBC
RPLP0
RPS20
NDRG1
CANX
HLA−DRB5
ANGPTL4
NNMT
C3
HLA−A
HLA−C
HLA−DRB1
HLA−E
CTSD
VIM
SPARC
IGFBP3
BGN
GAPDH
ACTB
EEF1A1
RPS11
B2M
HLA−B
HLA−DRA
RBPMS
SNRPB2
RB1
GLRX
ITGB2
HLA−DOA
P4HA2
SAP30
ABCA1
NAMPT
EDIL3
POSTN
TSC2
KLF7
ABI3BP
LOXL2
TRIB3
PGF
SLAMF7
FCGR1A
CEBPA
KCNMA1
STBD1
IFI30

Fig. S7: Heatmap of gene expression of the 113 candidates in non-tumor and ccRCC tissue of the TCGA patient cohort. Yellow indicates low expression, red indicates high expression. Expression fold-changes between tumor and non-tumor tissue are summarized in Tab. S10. Non-tumor tissues are marked by the green bar above the heatmap, tumor tissues are marked by the red bar.

References

1. Therneau TM, Lumley T. survival: Survival Analysis. R package version 2.44-1.1. 2019. https://CRAN.R-project.org/package=survival.
2. Hothorn T, Hornik K, Zeileis A. Unbiased Recursive Partitioning: A Conditional Inference Framework. J Comput Graph Stat. 2006;15:651–74. doi:10.1198/106186006X133933.
3. Rooney MS, Shukla SA, Wu CJ, Getz G, Hacohen N. Molecular and genetic properties of tumors associated with local immune cytolytic activity. Cell. 2015;160:48–61. doi:10.1016/j.cell.2014.12.033.
4. Fisel P, Kruck S, Winter S, Bedke J, Hennenlotter J, Nies AT, et al. DNA methylation of the SLC16A3 promoter regulates expression of the human lactate transporter MCT4 in renal cancer with consequences for clinical outcome. Clin Cancer Res. 2013;19:5170–81. doi:10.1158/1078-0432.CCR-13-1180.
5. Leuthold P, Schaeffeler E, Winter S, Buttner F, Hofmann U, Murdter TE, et al. Comprehensive Metabolomic and Lipidomic Profiling of Human Kidney Tissue: A Platform Comparison. J Proteome Res. 2017;16:933–44. doi:10.1021/acs.jproteome.6b00875.
6. PubChem. National Center for Biotechnology Information (NCBI), Berthesda, USA. 2017. https://pubchem.ncbi.nlm.nih.gov/. Accessed 29 Aug 2017.
